# Ropivacaine as a novel AKT1 specific inhibitor regulates the stemness of breast cancer

**DOI:** 10.1186/s13046-024-03016-9

**Published:** 2024-03-25

**Authors:** Lin Ding, Hui Jiang, Qiangwei Li, Qiushuang Li, Tian-Tian Zhang, Limeng Shang, Bin Xie, Yaling Zhu, Keshuo Ding, Xuanming Shi, Tao Zhu, Yong Zhu

**Affiliations:** 1 Department of Pathophysiology, School of Basic Medical Sciences, Anhui Medical University, Hefei, 230032 China; 2 Department of Anesthesiology, the First Affiliated Hospital of Anhui Medical University, Hefei, 230032 China; 3 School of Basic Medical Sciences, Anhui Medical University, Hefei, Anhui China; 4 Department of Oncology, The First Affiliated Hospital of USTC, Center for Advanced Interdisciplinary Science and Biomedicine of IHM, Division of Life Sciences and Medicine, University of Science and Technology of China, Hefei, Anhui 230027 China; 5 Department of Pathology, School of Basic Medicine, Anhui Medical University, Hefei, China; 6 Key Laboratory of Immune Response and Immunotherapy, Division of Life Sciences and Medicine, University of Science and Technology of China, Hefei, Anhui 230027 China; 7 Shenzhen Bay Laboratory, Shenzhen, 518055 China

**Keywords:** Cancer stem cell, Breast cancer, Ropivacaine, GGT1, NF-κB signaling pathway

## Abstract

**Background:**

Ropivacaine, a local anesthetic, exhibits anti-tumor effects in various cancer types. However, its specific functions and the molecular mechanisms involved in breast cancer cell stemness remain elusive.

**Methods:**

The effects of ropivacaine on breast cancer stemness were investigated by in vitro and in vivo assays (i.e., FACs, MTT assay, mammosphere formation assay, transwell assays, western blot, and xenograft model). RNA-seq, bioinformatics analysis, Western blot, Luciferase reporter assay, and CHIP assay were used to explore the mechanistic roles of ropivacaine subsequently.

**Results:**

Our study showed that ropivacaine remarkably suppressed stem cells-like properties of breast cancer cells both in vitro and in vivo. RNA-seq analysis identified GGT1 as the downstream target gene responding to ropivacaine. High GGT1 levels are positively associated with a poor prognosis in breast cancer. Ropivacaine inhibited GGT1 expression by interacting with the catalytic domain of AKT1 directly to impair its kinase activity with resultant inactivation of NF-κB. Interestingly, NF-κB can bind to the promoter region of GGT1. KEGG and GSEA analysis indicated silence of GGT1 inhibited activation of NF-κB signaling pathway. Depletion of GGT1 diminished stem phenotypes of breast cancer cells, indicating the formation of NF-κB /AKT1/GGT1/NF-κB positive feedback loop in the regulation of ropivacaine-repressed stemness in breast cancer cells.

**Conclusion:**

Our finding revealed that local anesthetic ropivacaine attenuated breast cancer stemness through AKT1/GGT1/NF-κB signaling pathway, suggesting the potential clinical value of ropivacaine in breast cancer treatment.

**Supplementary Information:**

The online version contains supplementary material available at 10.1186/s13046-024-03016-9.

## Introduction

Breast cancer is a leading cause of cancer-related mortality in women globally, posing a significant threat to their health and well-being. Cancer relapse and metastasis are considered as the primary drivers of death [1, 2]. Cancer stem cells (CSCs) represent a small subpopulation of tumor cells sharing similar properties with normal stem or progenitor cells, which have been intricately associated with cancer metastasis [3]. Several key developmental or signaling pathways, specifically, the Janus-activated kinase/signal transducer and activator of transcription, Hedgehog, Wnt, Notch, phosphatidylinositol 3-kinase/phosphatase and tensin homolog, and nuclear factor-κB signaling pathways have all been shown to mediate various CSC properties [4–6]. The subpopulation of breast cancer cells with high level of stem cell markers (CD44, ALDH1, CD133, OCT4, SOX2 and Nanog) exhibit the capacities of self-renewal and multi-directional differentiation, playing critical roles in tumor initiation, therapeutic resistance, relapse, and metastasis [7–9]. Thus, a comprehensive exploration of the molecular mechanisms underlying the role of CSCs in breast cancer progression is essential for the development of new diagnostic and therapeutic strategies.

Surgical resection is still the most effective strategy for cancer therapy. An increasing body of evidence suggests that perioperative care and anesthetic management are closely linked to the prognosis of cancer patients [10, 11]. Interestingly, ropivacaine, one of the most commonly used local anesthetics in surgical anesthesia [12], exhibits substantial anti-tumor effects in various solid tumors [7, 13, 14]. Furthermore, it was also reported that ropivacaine served as a tumor-suppressive agent in breast cancer progression [15–17]. Nevertheless, whether ropivacaine could regulate the stem cell-like properties of breast cancer cells remain poorly understood.

Gamma-glutamyltransferase 1 (GGT1), is a type I gamma-glutamyltransferase that catalyzes the transfer of the glutamyl moiety of glutathione to a variety of amino acids and dipeptide acceptors [18, 19]. GGT1 has been implicated as an oncogenic factor in glioblastoma and clear cell renal cell carcinoma [20, 21]. Furthermore, Li et al. demonstrated that HLF promoted the ferroptosis resistance in triple-negative breast cancer cells through GGT1, facilitating malignant tumor progression [22]. However, the specific role of GGT1 in breast cancer progression remains largely unexplored.

This study aimed to uncover the effects of ropivacaine on the cancer stem cell-like phenotypes of breast cancer cells and elucidate the underlying molecular mechanisms. Here, we demonstrated that ropivacaine significantly suppressed the stemness and chemoresistance in breast cancer cells by serving as the AKT1 specific inhibitor and subsequent repressing the expression of GGT1. Our results emphasize the critical implications of selecting perioperative anesthetic drugs for breast cancer patients.

## Materials and methods

### Cell lines and reagents

Cell lines were cultured as previously [23, 24]. The doxorubicin-resistant MCF-7 cells (MCF-7/ADR) were established by a stepwise increase of doxorubicin concentrations in the culture over 6 months to achieve statistically significant degrees of resistance relative to parental MCF-7 cell line. The initial and final concentrations of doxorubicin were 2 μM and 100 μM in the medium. All cell lines were authenticated through STR profiling and tested monthly for mycoplasma contamination by PCR. Ropivacaine hydrochloride (HYB0563B) was purchased from MedChemExpress LLC (NJ, USA), 10 μM and 40 μmol/kg (intraperitoneal injection, once every other day) were used in in vitro assays and in vivo studies, respectively. Doxorubicin, BAY 117082, recombinant AKT1 and ATP agarose beads were acquired from Sigma-Aldrich (Merck KGaA).

### Patients and tissue samples

A total of 70 breast cancer tissues with their paired adjacent normal mammary tissues were collected from the First Affiliated Hospital of Anhui Medical University (Hefei, Anhui, People’s Republic of China). All patients enrolled signed their informed consent before surgery and did not suffer from other malignancy or received radiotherapy or chemotherapy. Tumor tissues and adjacent normal tissues were confirmed by a pathologist and frozen with liquid nitrogen to prevent protein degradation. The human breast cancer tissues used in this study have received consent from the Ethics Committee of the Anhui Medical University and conducted in conformity to the Declaration of Helsinki.

### Flow cytometry

To assess percentage of cancer stem cells, MCF-7 and MDA-MB-231 cells with different treatments were resuspended in PBS containing 0.5% FBS and stained with anti-CD24-PE and anti-CD44-FITC for 30 min at 4 °C in the dark. Cells were washed with PBS and then analyzed by a Cyto FLEX flow cytometry (Beckman, Germany). Cell apoptosis of MCF-7/ADR cells with different treatments was determined with the Annexin V staining kit (cat. no., KGA106; Nanjing KeyGen Biotech, Co., Ltd.) following the instruction manual. All antibodies used were list in Supplementary Table 1.

### Mammosphere formation assay

MCF-7 and MDA-MB-231 cells with different treatments were seeded in ultra-low attachment dishes (Corning) at the density of 5,000 cells per well and cultured in serum-free DMEM/F12 medium (Invitrogen) supplemented with factors as previously described [25]. About 2 weeks later, images were taken at 4 × magnification and counted.

### Transwell assays

Cell migration and invasion were carried out as we decrcibed previously [23].

### MTT assay

MCF-7/ADR cells with different treatments were seeded into 96-well plates at 1,000 cells per well, and subsequently cultured for 48 h with indicated dose of doxorubicin. Cell viability was determined by using the MTT reagent (Promega).

### Western blot

Protein extraction and Western blot were performed as decrcibed previously [23]. All antibodies used were list in Supplementary Table 1.

### Animal models

To measure lung metastasis of breast cancer cells, 3 × 10^5^ MDA-MB-231 cells with different treatments were injected into 6-week old female BALB/c nude mice (*n* = 4) via tail vein. A week later, metastatic lung lesions were monitored by bioluminescence imaging once a week. The mice were intraperitoneally injected with d-luciferin firefly (Gold BioTechnology) at a dose of 3 mg per mouse and the bioluminescence images were acquired 15 min after injection. Two months later, all mice were sacrificed and tissues were harvested for subsequent assays.

To examine tumor-initiating capacity of breast cancer cells, A series dilution of MDA-MB-231 cells (1 × 10^6^, 1 × 10^5^, 1 × 10^4^) with different treatments were mixed 1:1 with Matrigel (BD Biosciences) and injected into the second mammary fat pad of the nude mice (*n* = 5/6). The time and efficiency of tumor formation in each group were recorded. Seven weeks later, mice were sacrificed and tumors were resected.

All animal experiments were approved by the Animal Ethical Committee of Anhui Medical University.

### Histology and Immunohistochemistry (IHC)

H&E-staining and Ki-67 staining were performed as we decrcibed previously [25].

### RNA-seq analysis

MCF-7 cells treated with ropivacaine (10 μM) or negative control for 48 h and shCtrl- or shGGT1-MCF-7 cells were harvested, RNA sequencing were performed by Genergy BIO-TECHNOLOGY Company (Shanghai, China) after quality assessment of library preparation.

### Quantitative PCR analysis (qRT-PCR)

RNA extraction and qRT-PCR analysis were conducted as decrcibed previously [24]. All primers used were list in Supplementary Table 2.

### Cell transfection

GGT1 was stably knockdown in MCF-7 and MDA-MB-231 cells transfected with lentivirus containing shRNAs against GGT1. siRNAs against GGT1, GGT1 and p65 overexpression plasmid were synthesised in GenePharma (Shanghai, China) and transfected into cells by using Lipofectamine 2000 (Invitrogen) according to the manufacturer’s instructions. Oligonucleotides used were listed in Supplementary Table 2.

### Luciferase reporter assay

GGT1 promoter (2 kb) was amplified and inserted into pGL3-Basic luciferase reporters vectors. pRL-TK plasmid was provided as an internal transfection control. The detailed protocol of luciferase reporter assays was described in the previously study [26]. NF-κB reporter assay was conducted as recommeded [27]. All primers used were listed in Supplementary Table 2.

### ChIP assay

Chromatin immunoprecipitation was performed using the ChIP Assay kit (Beyotime) and carried out following the manufacturer’s instructions. DNA enrichment was assessed by PCR using PrimeStar HS DNA Polymerase (Takara). The primers used are listed in Supplementary Table 2.

### Molecular docking

To analyze the binding affinities and modes of interaction between the drug candidate and their targets, AutodockVina 1.2.2, a silico protein–ligand docking software was employed [28]. The molecular structures of Ropivacaine hydrochloride (PubChem CID: 175804) was retrieved from PubChem Compound (https://pubchem.ncbi.nlm.nih.gov/) [29]. The PDB format files of the AKT1(PDB ID: P31749) were generated from the AlphaFold Protein Structure Database (https://alphafold.ebi.ac.uk/) [30]. For docking analysis, all protein and molecular files were converted into PDBQT format with all water molecules excluded and polar hydrogen atoms were added. The grid box was centered to cover the domain of each protein and to accommodate free molecular movement. The grid box was set to 30 Å × 30 Å × 30 Å, and grid point distance was 0.05 nm. Molecular docking studies were performed by Autodock Vina 1.2.2 (http://autodock.scripps.edu/).

### Cellular thermal shift assay (CETSA)

Cell lysates of MCF-7 and MDA-MB-231 cells with different treatments were aliquoted and heated to the indicated temperatures (40–70 ◦C) for 3 min in a thermal cycler and then cooled for 3 min at 4 ◦C, followed by centrifugation. The supernatant was subjected to Western blot analysis.

### Kinase activity assay

The ADP-Glo™ Kinase Assay Kit (Promega) was used to assess AKT1 kinase activity. Briefly, active AKT1, DMSO, or the indicated concentrations of ropivacaine and 100 μM ATP was incubated in kinase buffer at 30 ◦C for 1 h, followed by the addition of ADP-Glo™ reagent and incubation at 25 ◦C for 40 min. Kinase activity was detected by measuring the luminescence with a microplate reader.

### ATP competition assay

Recombinant AKT1 and the indicated concentrations of ropivacaine were incubated at 4 ◦C for 1 h. ATP agarose beads were then added and incubated at 4 ◦C for another 2 h in a shaker. The beads were washed five times, and then subjected to Western blot analysis.

### Statistical analysis

Data processing and analyses were performed using GraphPad Prism (San Diego, CA). Data are presented as mean (from at least three independent experiments) ± standard deviation. Differences between groups were analyzed with Student’s t-test or two-way ANOVA. Kaplan–Meier analysis was employed for the statistical comparison of overall survival and tumor-free survival rates. *P* < 0.05 was considered statistically significant.

## Results

### Ropivacaine inhibits CSC-like properties of breast cancer cells

**Fig. 1:**
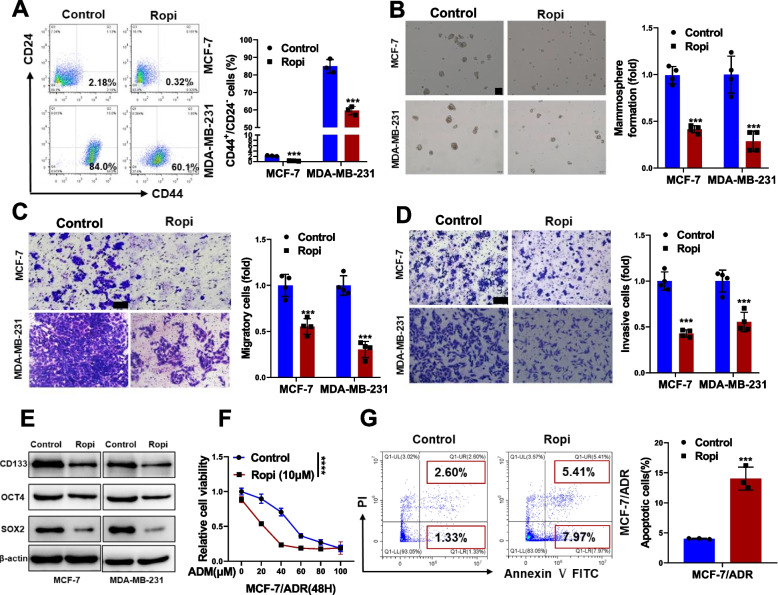
Effects of ropivacaine on CSC-like phenotypes of breast cancer cells in vitro.** A** CD44^+^/CD24.^−^ subpopulation in MCF-7 and MDA-MB-231 treated with ropivacaine (10 μM) or negative control for 48 h was measured by FACS analysis. **B** Mammosphere formation of MCF-7 and MDA-MB-231 treated with ropivacaine (10 μM) or negative control for 48 h (scale bar = 100 μm). **C-D** Migration and invasion of MCF-7 and MDA-MB-231 treated with ropivacaine (10 μM) or negative control for 48 h were examined by transwell assays (scale bar = 100 μm). **E** Stem cell markers (CD133, OCT4, SOX2) in MCF-7 and MDA-MB-231 treated with ropivacaine (10 μM) or negative control for 48 h were analyzed by western blot. **F** Cell viability of ropivacaine (10 μM)- or negative control-treated MCF-7/ADR cells exposed to the indicated concentration of ADR. **G** Cell apoptosis of ropivacaine (10 μM)- or negative control-treated MCF-7/ADR cells exposed to ADR (10 μM) were assessed by FACS analysis. Results are shown are shown as mean ± S.D from at least three independent experiments. *p < 0.05; **p < 0.01; ***p < 0.001 (Two-way ANOVA test in F, others unpaired two-tailed Student’s t test)

To determine the effects of ropivacaine on the CSC-like properties of breast cancer cells, MCF-7 and MDA-MB-231 cells were treated with ropivacaine and subjected to functional characterization. A significant decreased CD44^+^/CD24^−^ CSC subpopulations (Fig. 1A) and mammosphere formation (Fig. 1B) were observed in breast cancer cells upon ropivacaine treatment. We also observed ropivacaine significantly inhibited migration and invasion of breast cancer cells (Fig. 1C and D). Consistently, ropivacaine treatment resulted in reduced expression levels of stem cell markers, including CD133, OCT4 and SOX2 (Fig. 1E). Given that CSCs are associated with chemoresistance [31], whether ropivacaine could resensitize doxorubicin-resistant MCF-7 cells (MCF-7/ADR) to doxorubicin was examined. As anticipated, ropivacaine treatment decreased cell viability (Fig. 1F) and increased apoptosis (Fig. 1G) in MCF-7/ADR cells treated with doxorubicin. Taken together, our results suggest ropivacaine inhibits the stemness of breast cancer cells.

### Ropivacaine suppresses metastasis and tumorigenic capacity

**Fig. 2:**
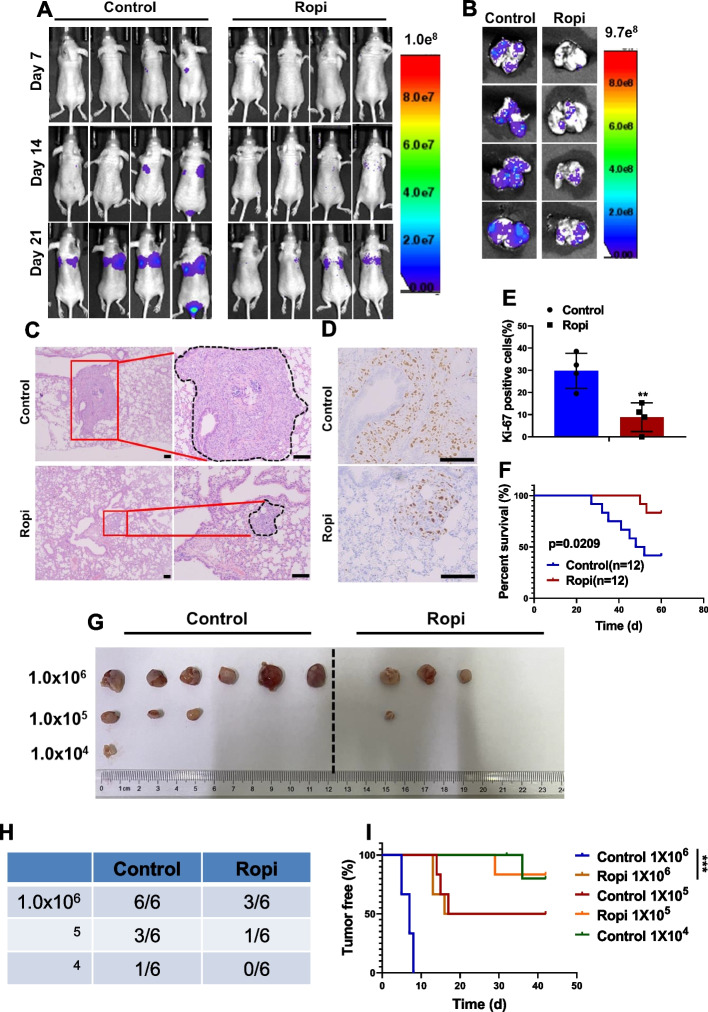
Effects of ropivacaine on CSC-like phenotypes of breast cancer cells in vivo. **A** Bioluminescence images of the metastatic burden of ropivacaine (40 μmol/kg, intraperitoneal injection, once every other day)- or negative control-treated mice at the indicated days after injection with MDA-MB-231 cells intravenously (*n* = 4). **B-C** Bioluminescence images (**B**) and H&E staining (**C**) of lungs from ropivacaine- or negative control-treated mice intravenously injected with MDA-MB-231 cells (scale bar = 100 μm) (*n* = 4). **D-E** Ki-67 staining of lung sections from ropivacaine- or negative control-treated mice intravenously injected with MDA-MB-231 cells (scale bar = 100 μm) (*n* = 4). **F** Survival curve of mice intravenously injected with MDA-MB-231 cells and treated with ropivacaine or negative control (*n* = 12). **G-H** The incidence of tumors in mice injected with different numbers of MDA-MB-231 cells and treated with ropivacaine or negative control (*n* = 6). **I** The tumor-free survival curves of the mice that were inoculated with different numbers of MDA-MB-231 cells with and without ropivacaine treatment (*n* = 6). *p < 0.05; **p < 0.01; ***p < 0.001 (log-rank test in **F** and **I**, others unpaired two-tailed Student’s t test)

To assess the effects of ropivacaine on the CSCs in vivo, a tail-vein injection model was employed to establish lung metastasis by using the MDA-MB-231 cells. Bioluminescence imaging revealed a significant reduction in pulmonary metastasis in mice treated with ropivacaine (Fig. 2A and B), which was confirmed by H&E staining of the lung sections from each group (Fig. 2C). Additionally, ropivacaine treatment led to decreased cell proliferation in the lung metastatic lesions (Fig. 2D and E), indicating its inhibitory effects on the colonization of metastatic cells. A higher survival rate for the tumor-bearing mice in ropivacaine-treated group was also observed as a result (Fig. 2F).

We also evaluated the effect of ropivacaine on the tumor-initiating capability of breast cancer cells by using limited dilution assay. 1 × 10^6^ MDA-MB-231 cells formed tumor xenografts with 100% efficiency in the control group, but the tumor formation efficiency of the ropivacaine-treated group decreased to 60%. When 1 × 10^5^ and 1 × 10^4^ cells per site were implanted, the tumor formation efficiency in ropivacaine-treated group decreased to 20% and 0%, whereas the control group retained 60% and 40%, respectively (Fig. 2G and H). Not surprisingly, the onset of tumor growth in the ropivacaine-treated group was delayed compared with its control counterparts (Fig. 2I). Collectively, our study demonstrates that ropivacaine exhibits anti-CSC effects in vivo.

### Ropivacaine represses GGT1 expression

**Fig. 3:**
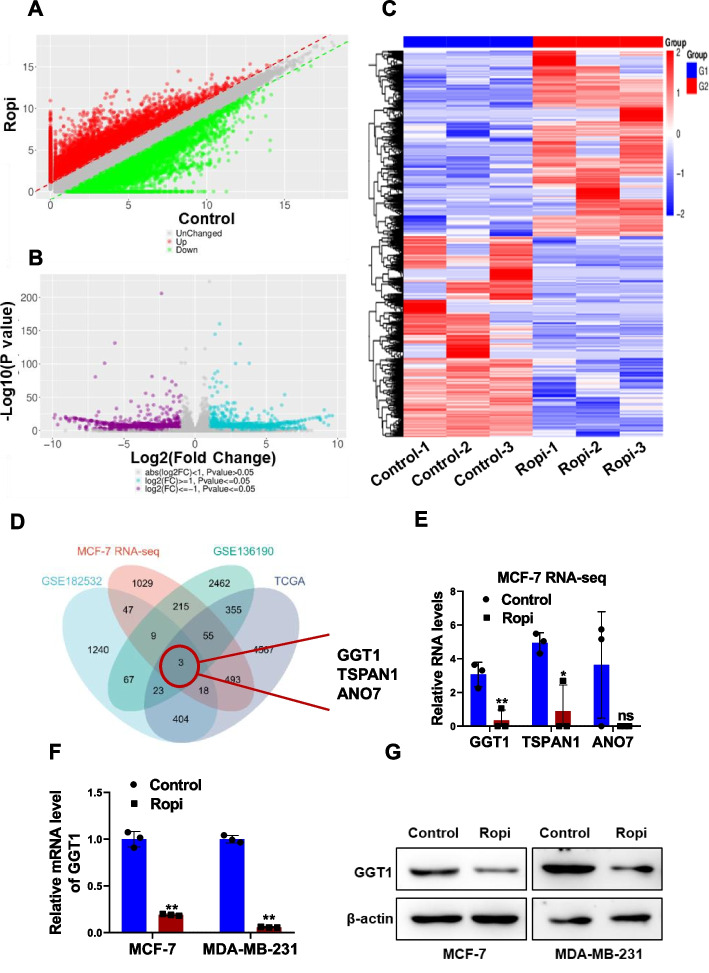
Identification of GGT1 as a ropivacaine-responding gene in breast cancer cells. **A-B** RNA-seq data of ropivacaine- or negative control-treated MCF-7 cells. The scatter plot (**A**) and volcano map (**B**) showing differentially-expressed genes. **C** Heatmap representing the top 50 up-regulated and down-regulated genes in MCF-7 cells treated with ropivacaine or negative control. **D** The intersection among four databases. **E** RNA levels of GGT1, TSPAN1 and ANO7 in MCF-7 cells treated with ropivacaine or negative control from RNA-seq data. **F-G** RNA (**F**) and protein (**G**) level of GGT1 in MCF-7 and MDA-MB-231 treated with ropivacaine (10 μM) or negative control for 48 h were was determined by qRT-PCR and western blot, respectively. Results are shown are shown as mean ± S.D from at least three independent experiments. *p < 0.05; **p < 0.01; ***p < 0.001 (unpaired two-tailed Student’s t test)

We subsequently conducted RNA-seq analysis of MCF-7 cells treated with or without ropivacaine for the mechanistic insight. The scatter plot, volcano plot and heat map displayed dysregulated genes in MCF-7 cells in response to ropivacaine treatment (Fig. 3A-C). The upregulated genes in mammosphere cells compared with their parental MCF-7 and MDA-MB-231 cells were screened out in silico (GSE182532 and GSE136190). Upregulated genes in breast cancer tissues compared to their normal counterparts from TCGA dataset were selected. By combining the data from all these four cohorts, we focused on three candidate genes: GGT1, TAPAN1 and ANO7 (Fig. 3D). Among the identified genes, GGT1 emerged as the most down-regulated target responding to ropivacaine treatment (Fig. 3E). We further validated the suppressive effects of ropivacaine on GGT1 at the RNA and protein levels in MCF-7 and MDA-MB-231 cells using qRT-PCR and Western blot analysis (Fig. 3F and G). These results suggest GGT1 is a downstream target gene of ropivacaine.

### NF-κB is an upstream transcriptional factor of GGT1

**Fig. 4:**
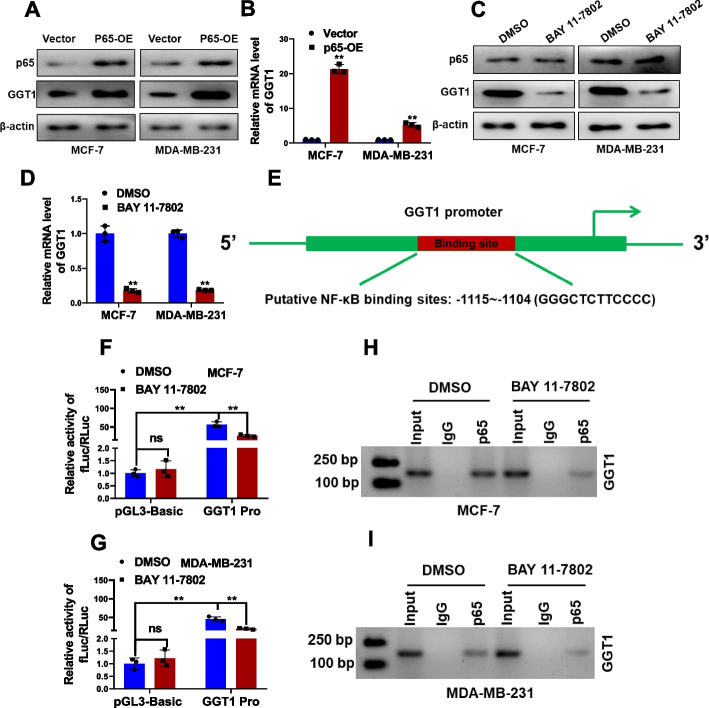
NF-κB binds to the promoter region of GGT1 to activate its transcription. **A** Protein levels of GGT1 and p65 in MCF-7 and MDA-MB-231 cells transfected with p65 overexpression plasmid (P65-OE) or empty vector were examined by western blot. **B** RNA level of GGT1 in MCF-7 and MDA-MB-231 cells transfected with p65 overexpression plasmid (P65-OE) or empty vector was examined by qRT-PCR. **C** Protein levels of GGT1 and p65 in MCF-7 and MDA-MB-231 cells treated with BAY 11–7802 (1 μM) or DMSO were examined by western blot. **D** RNA level of GGT1 in MCF-7 and MDA-MB-231 cells treated with BAY 11–7802 (1 μM) or DMSO was examined by qRT-PCR. **E** Schematic of predicted binding sites between GGT1 promoter and p65. **F-G** Construction of luciferase reporter vectors comprising p65 binding sites in the DNA promoter region of GGT1 (2 kb). Dual-luciferase reporter assays were performed by transfecting the promoter region of GGT1 (GGT1 Pro) or pGL3-basic plasmid in MCF-7 and MDA-MB-231 cells treated with BAY 11–7802 (1 μM) or DMSO. **H-I** The binding of p65 to the GGT1 promoter region in MCF-7 and MDA-MB-231 cells treated with BAY 11–7802 (1 μM) or DMSO was examined by ChIP assay. Results are shown are shown as mean ± S.D from at least three independent experiments. *p < 0.05; **p < 0.01; ***p < 0.001 (unpaired two-tailed Student’s t test)

For mechanistic insight of ropivacaine repressed GGT1 expression, we employed the PROMO (https://alggen.lsi.upc.es/cgi-bin/promo_v3/promo/promoinit.cgi?dirDB=TF_8.3) online prediction tool to identify potential upstream transcriptional factors of GGT1. NF-κB was predicted to potentially bind to the GGT1 promoter region. This prediction was supported as p65 overexpression significantly increased GGT1 expression (Fig. 4A and B), while the NF-κB inhibitor, BAY 11–7802, reduced GGT1 expression (Fig. 4C and D), at both protein and RNA levels in MCF-7 and MDA-MB-231 cells. Further, luciferase reporter assay was employed by inserting GGT1 promoter region containing NF-κB-binding sites into pGL3-basic reporter vector (Fig. 4E). BAY 11–7802 remarkably inhibited GGT1 promoter elicited luciferase activity (Fig. 4F and G). ChIP-PCR assays was preformed to have confirmed the reduced enrichment of p65 at the GGT1 promoter region upon NF-κB inhibition (Fig. 4H and I). These results collectively demonstrate that NF-κB is a bona fide upstream transcriptional factor of GGT1 in breast cancer cells.

### Ropivacaine interacts with AKT1 directly to repress NF-κB signaling

**Fig. 5:**
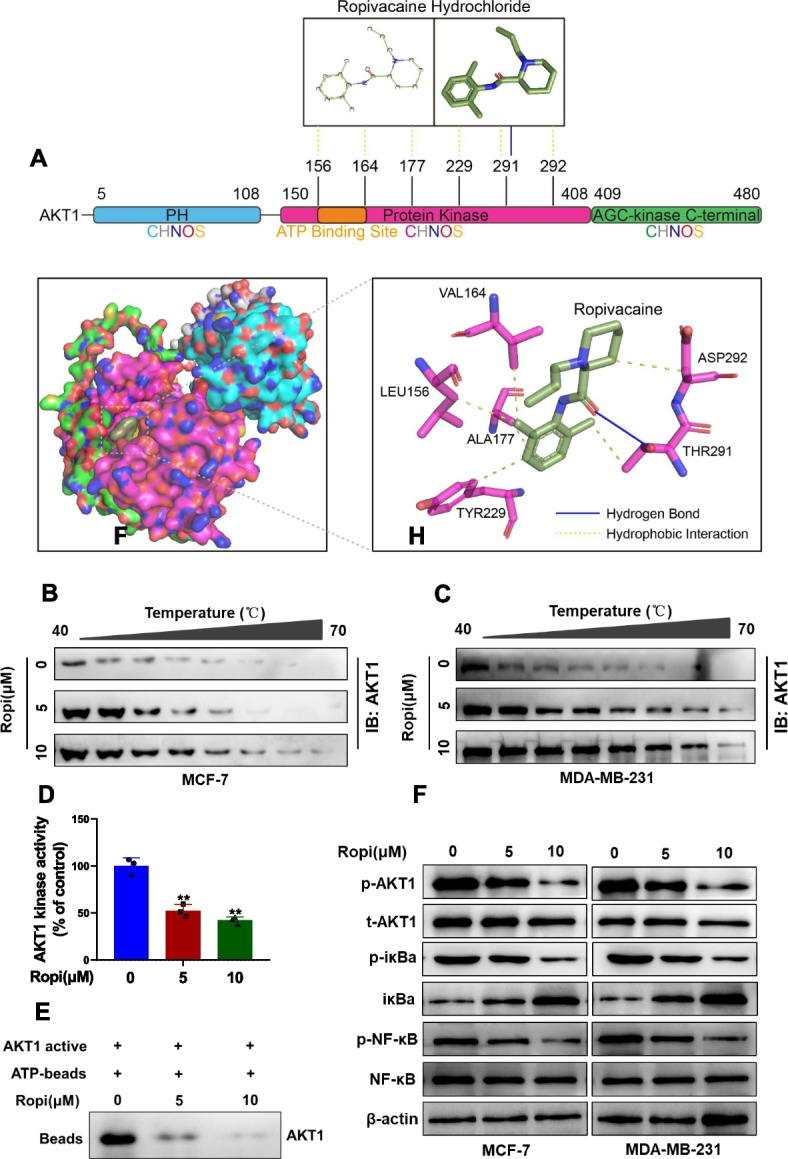
Ropivacaine interacts with AKT1 directly to repress NF-κB signaling. **A** The 2D and 3D structure of ropivacaine hydrochloride and the predicted binding modes of ropivacaine hydrochloride with AKT1 kinase domain. **B**-**C** The stabilizing effect of ropivacaine on AKT1 protein was assessed by western blot in cellular thermal shift assay using the indicated concentrations of ropivacaine-treated MCF-7 and MDA-MB-231 cells. **D** The effect of ropivacaine on the activity of AKT1 kinase has been estimated through kinase activity assay. **E** The competitive binding relationship between ropivacaine and ATP was confirmed by using a pull-down assay. **F** Protein levels of p-AKT1, t-AKT1, p-iκBa, iκBa, p-NF-κB and NF-κB in the indicated concentrations of ropivacaine-treated MCF-7 and MDA-MB-231 cells were determined by western blot. Results are shown are shown as mean ± S.D from at least three independent experiments. *p < 0.05; **p < 0.01; ***p < 0.001 (unpaired two-tailed Student’s t test)

To identify the direct target of ropivacaine, SwissTargetPrediction (http://www.swisstargetprediction.ch/) with further bioinformatics analysis were performed to have suggested AKT1 as a potential direct binding partner. It is intriguing to note that AKT1 is widely known to activate NF-κB signaling pathway via catalyzing phosphorylation of iκB and releasing its inhibitory effect on NF-κB [32–34]. To confirm the direct interactions between ropivacaine and AKT1, a docking analysis of ropivacaine (the 2D and 3D structures are shown in Fig. 5A) and human AKT1 was performed by using Auto Dock Vina software. The binding poses and interactions of ropivacaine hydrochloride with AKT1 were obtained with Autodock Vina v.1.2.2 and binding energy for interaction was generated (Fig. 5A). Results showed that ropivacaine hydrochloride bound to domain area of AKT1 kinase through a hydrogen bond (THR291) and six hydrophobic interactions (LEU156, VAL164, ALA177, TYR229, THR291, ASP292) (Fig. S1). Moreover, the predicted ropivacaine-binding region partially coincided with the ATP-binding region (from LEU156 to VAL164), suggesting that ropivacaine as a potential ATP-competitive inhibitor of AKT1. Ropivacaine hydrochloride and AKT1 exhibited low binding energy value of -6.567 kcal/mol, suggestinga highly stable binding. Further, CETSA showed that ropivacaine treatment effectively protected the AKT1 protein from temperature-dependent degradation (Fig. 5B and C). Consistently, ropivacaine significantly reduced AKT1 kinase activity, as determined by the ADP-Glo™ kinase assay (Fig. 5D) and led to a notably decrease in the capacity of AKT1 to bind to ATP (Fig. 5E). As expected, the interaction between ropivacaine and AKT1 resulted in markedly decreased expression of p-AKT1, p-iκBa and p-NF-κB (Fig. 5F). Taken together, these data indicate ropivacaine inhibits NF-κB activity and GGT1 expression via its direct binding to AKT1.

### Elevated GGT1 is associated with poor prognosis of breast cancer

**Fig. 6:**
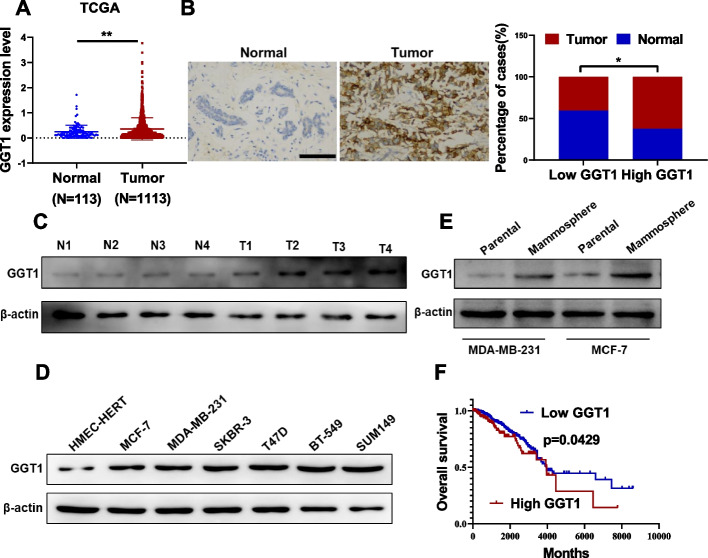
Higher GGT1 correlates to shorter overall survival of breast cancer patients. **A** mRNA level of GGT1 in 1113 breast cancer tissues and 113 benign normal tissues from TCGA database. **B** IHC analysis of GGT1 expression in breast cancer (Tumor) and peritumor tissues (Normal) from our cohort (*n* = 70), (scale bar = 100 μm). **C** Protein level of GGT1 in 4 breast cancer tissues and their adjacent normal tissues was determined by western blot. **D** Protein level of GGT1 in six breast cancer cell lines (MCF-7, MDA-MB-231, SKBR-3, T47D, BT-549 and SUM149) and a non-transformed mammary epithelial cell line (HMEC-hTERT) was determined by western blot. **E** Protein level of GGT1 in mammosphere derived from MCF-7 and MDA-MB-231 cells and their parental cells was evaluated by western blot. **F** Kaplan–Meier analysis of the relationship between GGT1 expression levels and overall survival of breast cancer patients from TCGA database. *p < 0.05; **p < 0.01; ***p < 0.001 (log-rank test in **F**, others unpaired two-tailed Student’s t test)

Analysis of GGT1 RNA levels in breast cancer tissue samples from the TCGA datasets revealed higher GGT1 expression compared to normal tissue (Fig. 6A). In consistence, IHC analysis from our own 70 patients' cohort also validated GGT1 overexpression compared with peritumor specimens (Fig. 6B). As showed in Supplementary Table 3, further analysis of correlation between GGT1 expression and clinicopathological features of these enrolled breast cancer patients indicated GGT1 expression was positively correlated with tumor stage (*p* = 0.0258) and lymph node metastasis (*p* = 0.0266). We also showed that GGT1 expression was up-regulated in the tumor group relative to the matched adjacent samples (Fig. 6C). Compared to HMEC-hTERT cells, GGT1 expression levels were higher in 6 breast cancer cell lines (Fig. 6D). Furthermore, GGT1 was up-regulated in mammospheres as compared with their parental cells (Fig. 6E). Kaplan–Meier survival analysis displayed that breast cancer patients with high level of GGT1 exhibited a shorter overall survival, compared with patients with lower levels of GGT1 (Fig. 6F). Collectively, these results suggest elevated GGT1 predicts poor prognosis of breast cancer patients.

### GGT1 depletion impairs stemness of breast cancer cells

**Fig. 7:**
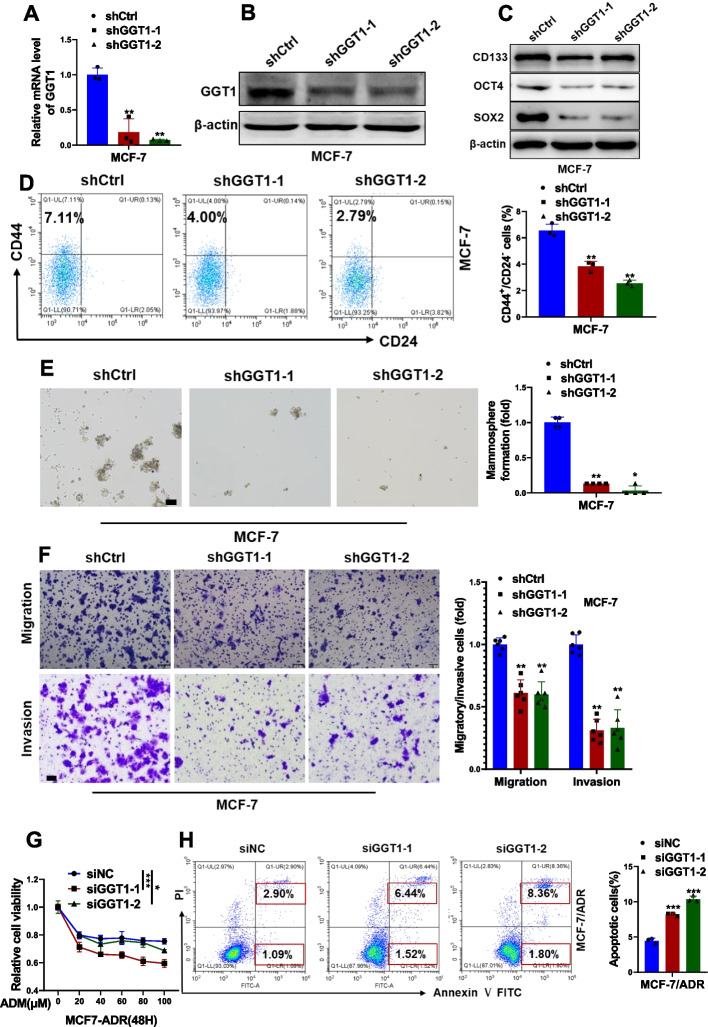
Effects of GGT1 on CSC-like phenotypes of MCF-7 cells in vitro. **A-B** RNA (**A**) and protein (**B**) level of GGT1 in MCF-7 cells stably transfected with shRNAs against GGT1 (shGGT1-1 and shGGT1-2) or negative control (shCtrl) were determined by qRT-PCR and western blot, respectively. **C** Stem cell markers (CD133, OCT4, SOX2) in GGT1-depleting MCF-7 cells were analyzed by western blot. **D** CD44^+^/CD24.^−^ subpopulation in GGT1-depleting MCF-7 cells was measured by FACS analysis. **E** Mammosphere formation of GGT1-depleting MCF-7 cells (scale bar = 100 μm). **F** Migration and invasion of GGT1-depleting MCF-7 cells were examined by transwell assays (scale bar = 100 μm). **G** Cell viability of GGT1-depleting MCF-7/ADR cells exposed to the indicated concentration of ADR. **H** Cell apoptosis of GGT1-depleting MCF-7/ADR cells exposed to ADR (10 μM) were assessed by FACS analysis. Results are shown are shown as mean ± S.D from at least three independent experiments. *p < 0.05; **p < 0.01; ***p < 0.001 (Two-way ANOVA test in **G**, others unpaired two-tailed Student’s t test)

To investigate the functional role of GGT1 in maintaining stemness in breast cancer cells, MCF-7 and MDA-MB-231 cells were stably transfected with shRNAs against GGT1 (Fig. 7A and B, Fig. S2A and B). GGT1 depletion led to significantly reduced expression of stem cell markers (Fig. 7C and Fig. S2C), decreased percentage of CD44^+^/CD24^−^ CSC subpopulations (Fig. 7D and Fig. S2D), impaired mammosphere formation (Fig. 7E and Fig. S2E), as well as reduced migratory and invasive capability (Fig. 7F and Fig. S2F) in MCF-7 and MDA-MB-231 cells. Endogenous GGT1 in MCF-7/ADR cells was obviously silenced by transfection with specific siRNAs targetting GGT1 (Fig. S3A and B). An attenuated cell viability (Fig. 7G) while increased cell apoptosis (Fig. 7H) were observed in GGT1-depleted MCF-7/ADR cells exposed to doxorubicin, suggesting that ablation of GGT1 restored the sensitivity of MCF-7/ADR cells to doxorubicin.

### GGT1 depletion dampens lung metastasis and tumor-initiating ability

**Fig. 8:**
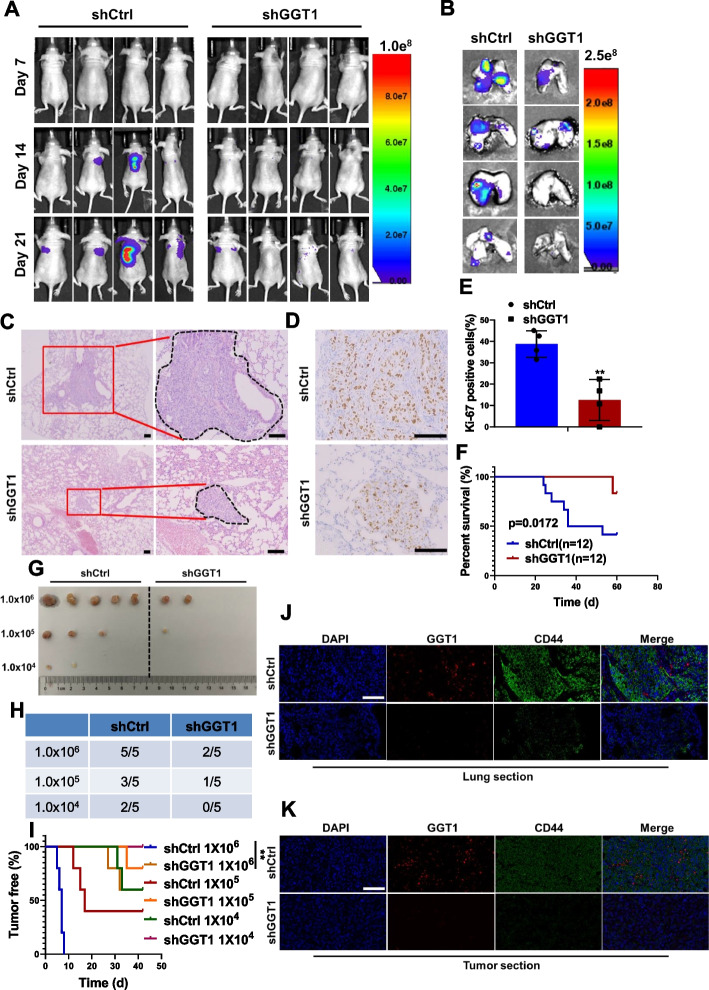
Effects of GGT1 on CSC-like phenotypes of breast cancer cells in vivo. **A** Bioluminescence images of the metastatic burden of mice at the indicated days after injection with MDA-MB-231-shCtrl or -shGGT1 cells intravenously (*n* = 4). **B-C** Bioluminescence images (**B**) and H&E staining (**C**) of lungs from mice intravenously injected with MDA-MB-231-shCtrl or -shGGT1 cells (scale bar = 100 μm) (*n* = 4). **D-E** Ki-67 staining of lung sections from mice intravenously injected with MDA-MB-231-shCtrl or -shGGT1 cells (scale bar = 100 μm) (*n* = 4). **F** Survival curve of mice intravenously injected with MDA-MB-231-shCtrl or -shGGT1 cells (*n* = 12). **G-H** The incidence of tumors in mice injected with different numbers of MDA-MB-231-shCtrl or -shGGT1 cells (*n* = 5). **I** The tumor-free survival curves of the mice that were inoculated with different numbers of MDA-MB-231-shCtrl or -shGGT1 cells (*n* = 5). **J-K** IF examination of GGT1 and CD44 expression in tumor sections from both lung tissues (**J**) and primary tumor tissues (**K**) derived from MDA-MB-231-shCtrl or -shGGT1 cells (scale bar = 100 μm) (*n* = 4).*p < 0.05; **p < 0.01; ***p < 0.001 (log-rank test in **F** and **I**, others unpaired two-tailed Student’s t test)

To further determine the roles of GGT1 in regulating CSC-like properties in vivo, 3 × 10^4^ MDA-MB-231 cells stably transfected with shRNAs against GGT1 or negative control were injected into Balb/c nude mice via tail vein. Bioluminescence imaging displayed that GGT1 depletion significantly inhibited lung metastasis derived from the MDA-MB-231 cells (Fig. 8A and B). H&E staining revealed smaller metastatic lung lesions and less incidence of lung metastasis in mice injected with GGT1-silenced MDA-MB-231 cells (Fig. 8C). Consistently, decreased cell proliferation in the lung metastatic lesions of mice derived from GGT1-depleted MDA-MB-231 cells was observed (Fig. 8D and E), indicating the role of GGT1 in promoting the colonization of metastatic cells. Moreover, mice injected with GGT1-depleted MDA-MB-231 cells exhibited a higher survival rate (Fig. 8F).

Limited dilution analysis was also conducted to determine whether GGT1 mediated tumorigenesis of breast cancer cells. 1 × 10^6^ MDA-MB-231/shCtrl cells formed tumor xenografts with 100% efficiency, whereas the tumor formation efficiency of MDA-MB-231/shGGT1 cells decreased to 40%. When cells were implanted at a density of 1 × 10^5^ and 1 × 10^4^ cells per site, the tumor formation efficiency of MDA-MB-231/shGGT1 cells decreased to 20% and 0%, but the MDA-MB-231/shCtrl cells retained 60% and 40% (Fig. 8G and H). Furthermore, the onset of tumor growth of MDA-MB-231/shGGT1 cells was much delayed compared with its control counterparts (Fig. 8I). Additionally, IF examination of tumor sections from both lung tissues and primary tumor tissues indicated GGT1 depletion accompanied by low level of CD44 (Fig. 8J and K). Collectively, these results highlight the role of GGT1 in maintaining stem-like phenotypes of breast cancer.

### GGT1 activates NF-κB signaling

**Fig. 9:**
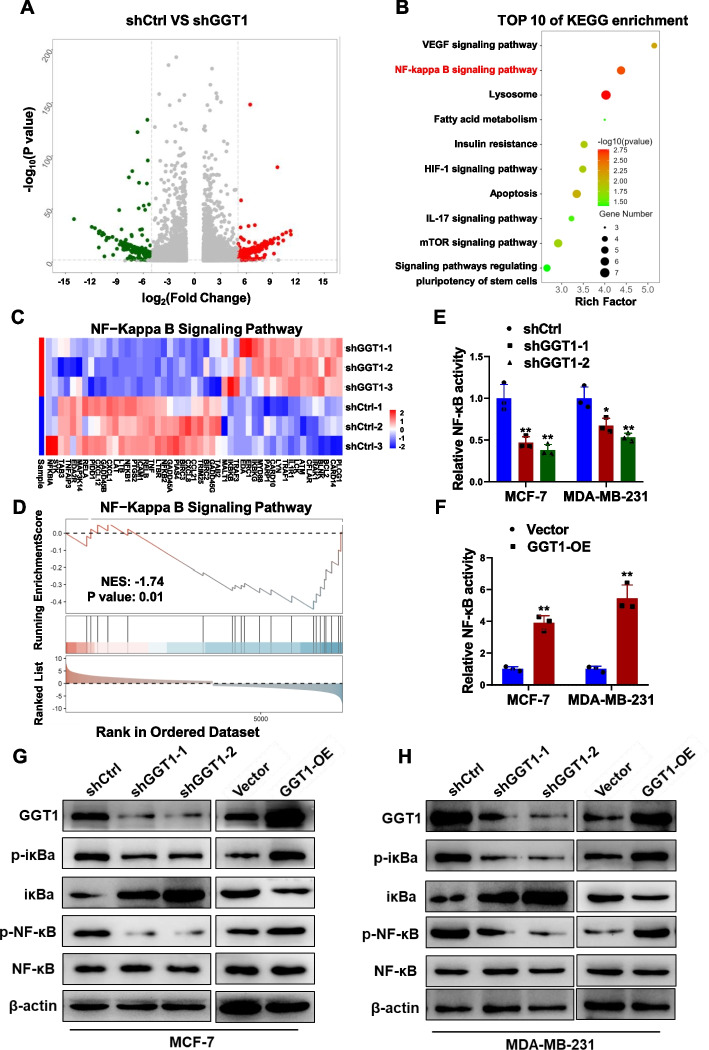
GGT1 enhances activation of NF-κB signaling pathway. **A** RNA-seq data of MCF-7-shCtrl or -shGGT1 cells. The volcano map showing differentially-expressed genes. **B** KEGG analysis of the top 10 enriched pathways in MCF-7-shCtrl or -shGGT1 cells from RNA-seq data. **C** Heatmap showing differentially expressed genes in NF-κB signaling pathway in MCF-7-shCtrl or -shGGT1 cells. **D** GSEA analysis of the enrichment of NF-κB signaling pathway in MCF-7-shCtrl or -shGGT1 cells from RNA-seq data. **E** NF-κB transcriptional activity in MCF-7 and MDA-MB-231 cells stably transfected with shRNAs against GGT1 (shGGT1-1 and shGGT1-2) or negative control (shCtrl) was determined by NF-κB activation reporter assay. **F** NF-κB transcriptional activity in MCF-7 and MDA-MB-231 cells transfected with GGT1 overexpression plasmid (GGT1-OE) or empty plasmid (Vector) was determined by NF-κB activation reporter assay. **G** Protein levels of GGT1, p-iκBa, iκBa, p-NF-κB and NF-κB in GGT1-depleting and GGT1-overexpressing MCF-7 and MDA-MB-231 cells were determined by western blot. Results are shown are shown as mean ± S.D from at least three independent experiments. *p < 0.05; **p < 0.01; ***p < 0.001 (unpaired two-tailed Student’s t test)

To elicit the signaling pathways affected by GGT1 in the regulation of breast cancer stem cell-like phenotypes, we conducted RNA-seq analysis in GGT1-depleted MCF-7 cells (shGGT1) and control cells. The volcano plot displayed differentially expressed genes (Fig. 9A). KEGG analysis revealed that GGT1 depletion impinged on several signaling pathways, with the NF-κB signaling pathway scoring on the top (Fig. 9B and C). Consistently, GSEA analysis indicated NF-κB signaling pathway was inactivated in MCF-7 cells with GGT1 depletion (Fig. 9D). We subsequently showed that GGT1 depletion impaired NF-κB activity (Fig. 9E), while GGT1 overexpression enhanced NF-κB activity (Fig. 9F). Further, knockdown of GGT1 reduced expression of p-iκBα and p-NF-κB, whereas forced expression of GGT1 exhibited opposite effects (Fig. 9G and H). Based on the essential role of NF-κB signaling pathway in maintaining stemness of breast cancer cells [35–37], we thereby propose GGT1 activates NF-κB signaling pathway in breast cancer cells.

### NF-κB/GGT1 feedback loop mediates inhibitory effects of ropivacaine on breast CSC-like phenotypes

**Fig. 10:**
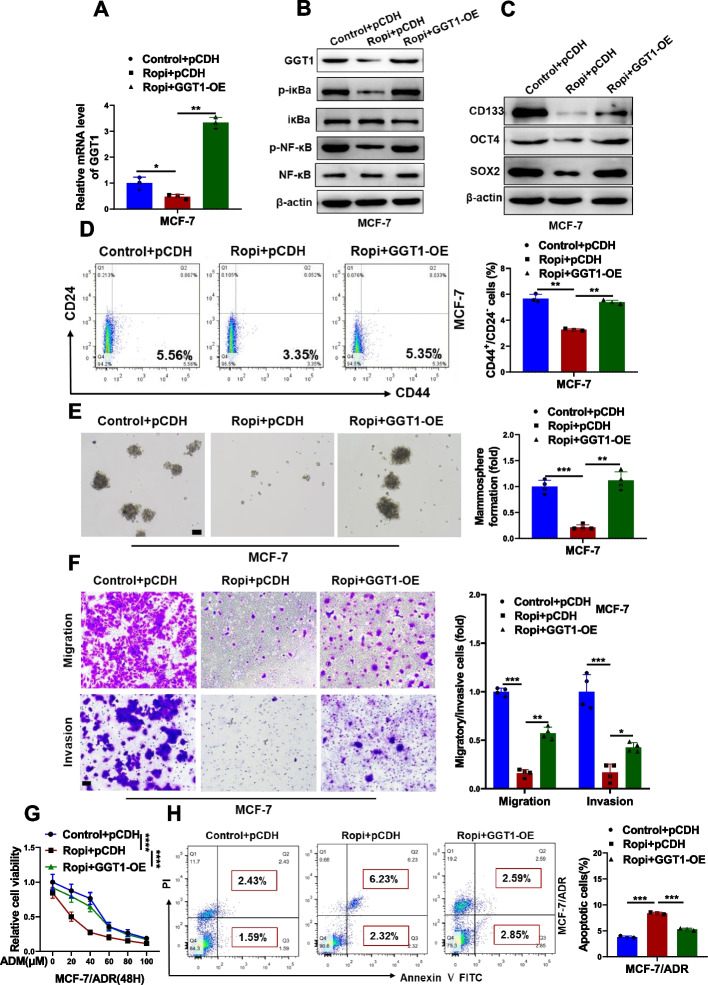
The anti-CSC effects of ropivacaine on MCF-7 cells is dependent on NF-κB/GGT1 feedback loop. MCF-7 cells was transfected with GGT1 overexpression plasmid (GGT1-OE) or empty plasmid (Vector) and treated with ropivacaine (10 μM) or negative control for 48 h. **A** RNA level of GGT1 in the indicated treated-MCF-7 cells was determined by qRT-PCR. **B** Protein levels of GGT1, p-iκBa, iκBa, p-NF-κB and NF-κB in the indicated treated-MCF-7 cells were examined by western blot. **C** Stem cell markers (CD133, OCT4, SOX2) in the indicated treated-MCF-7 cells were analyzed by western blot. **D** CD44^+^/CD24.^−^ subpopulation in the indicated treated-MCF-7 cells was measured by FACS analysis. **E** Mammosphere formation of the indicated treated-MCF-7 cells (scale bar = 100 μm). **F** Migration and invasion of the indicated treated-MCF-7 cells were examined by transwell assays (scale bar = 100 μm). **G** Cell viability of MCF-7/ADR cells transfected with GGT1 overexpression plasmid (GGT1-OE) or empty plasmid (Vector) and treated with ropivacaine (10 μM) or negative control for 48 h as indicated, exposure to the indicated concentration of ADR. **H** Cell apoptosis of the indicated treated-MCF-7/ADR cells exposed to ADR (10 μM) were assessed by FACS analysis. Results are shown are shown as mean ± S.D from at least three independent experiments.. *p < 0.05; **p < 0.01; ***p < 0.001 (Two-way ANOVA test in **G**, others unpaired two-tailed Student’s t test)

To determine whether ropivacaine-repressed stem cell-like traits is mediated by GGT1/NF-κB signaling pathway. MCF-7 and MDA-MB-231 cells were transfected with GGT1 ovexpression plasmid before ropivacaine treatment. Ropivacaine-induced suppression of GGT1, p-iκBα and p-NF-κB was significantly rescued by GGT1 overexpression (Fig. 10A and B, Fig. S4A and B). In line with GGT1/NF-κB signaling pathway, GGT1 overexpression reverted ropivacaine-repressed expression of stem cell markers (Fig. 10C and Fig. S4C), CD44^+^/CD24^−^ CSCs subpopulation (Fig. 10D and Fig. S4D), mamosphere formation (Fig. 10E and Fig. S4E), migration and invasion (Fig. 10F and Fig. S4F). Additionally, forcing expression of GGT1 dramatically restored ropivacaine-induced decreased cell viability (Fig. 10G) and increased cell apoptosis (Fig. 10H) in MCF-7/ADR cells exposed to doxorubicin. Taken together, these findings highlight GGT1/NF-κB feedback loop was involved in mediation of the anti-CSCs effects of ropivacaine.

## Discussion

Development of therapeutic resistance and metastasis is a major challenge with current breast cancer therapy. Breast cancer stem cells (CSCs) is gaining increasing attention for its pivotal role in breast cancer development, progression, and metastasis. In-depth study of the generation, regulatory mechanisms, and identification of CSCs in breast cancer and a better understanding of the strategies that are used by CSCs to resist conventional and targeted therapies, to interact with their niche, to escape immune surveillance, and finally to awaken from dormancy is of key importance to prevent and treat metastatic cancer [38, 39]. Therefore, targeting CSCs holds promise as a therapeutic strategy for breast cancer patients [40].

While the impact of anesthetic management on cancer progression attracted increasing attention in both clinical and basic research, whether anesthetics play a role in regulating CSC-like properties remained rather limited. For example, it was reported that abrogated circular RNA circNOLC1 expression by propofol/STAT3 attenuates breast cancer stem cell function through miR-365a-3p/STAT3 signaling [41]; Local anesthetics was also reported to impair the growth and self-renewal of glioblastoma stem cells by inhibiting ZDHHC15-mediated GP130 palmitoylation [42]. In this study, we presented the first evidence in breast cancer cells that ropivacaine, a widely used local anesthetic with great safety profile, whose pharmacokinetics has been well studied [43, 44], significantly suppresses CSC-like phenotypes. Although this study proposed the theoretical basis for perioperative anesthesia management in patients with breast cancer, whether ropivacaine could be developed as an independent drug for breast cancer treatment remained to be explored.

Several key signaling pathways have been implicated to be highly activated in maintainance of CSCs. Among which, NF-κB signaling pathway is extensively studied to mediate various stem cell properties, such as self-renewal, cell fate decisions, survival, proliferation, and differentiation [4]. Prior research has shown that the long noncoding RNA HOTAIR promotes the stemness of breast cancer cells through activation of the NF-κB signaling pathway [37], β-Catenin and NF-κB co-activation triggered by TLR3 stimulation facilitates stem cell-like phenotypes in breast cancer [45], and AXL induces epithelial-to-mesenchymal transition and regulates the function of breast cancer stem cells via activating NF-κB signaling pathway [46]. In this study, we identified GGT1 as the ropivacaine-regulated gene in breast cancer cells and subsequently demonstrated that GGT1 depletion abrogated CSC-like phenotypes, both in vitro and in vivo*.* Further analysis indicated GGT1 activated NF-κB signaling pathway, which is among the key signaling pathways implicated critically in the maintenance of CSCs [4], forming a positive feedback loop between NF-κB and GGT1. However, the exact mechanism by which GGT1 regulates the NF-κB signaling pathway remains to be determined in the future endeavor.

Our study further revealed the mechanism by which ropivacaine suppresses GGT1 expression. In accordance with GGT1-mediated CSC-like phenotypes, NF-κB was predicted and validated as the upstream transcription factor of GGT1. Fascinatingly, ropivacaine was identified to directly bind to AKT1, although previous studies have reported that ropivacaine inactivated the PI3K/AKT signaling pathway in keratinocytes and cancer cells [7, 13, 47]. In this study, we provide the first evidence that ropivacaine serves as an AKT1 specific inhibitor by abrogating the kinase activity of AKT via competition with ATP for binding to the ATP-binding pocket of AKT1, thus inhibiting the phosphorylation of IκBα, NF-κB, and GGT1 transcription. Thus, our study gains a deeper insight into the molecular mechanism by which ropivacaine impinges on AKT1 activity and represses GGT1 expression.

**Fig. 11:**
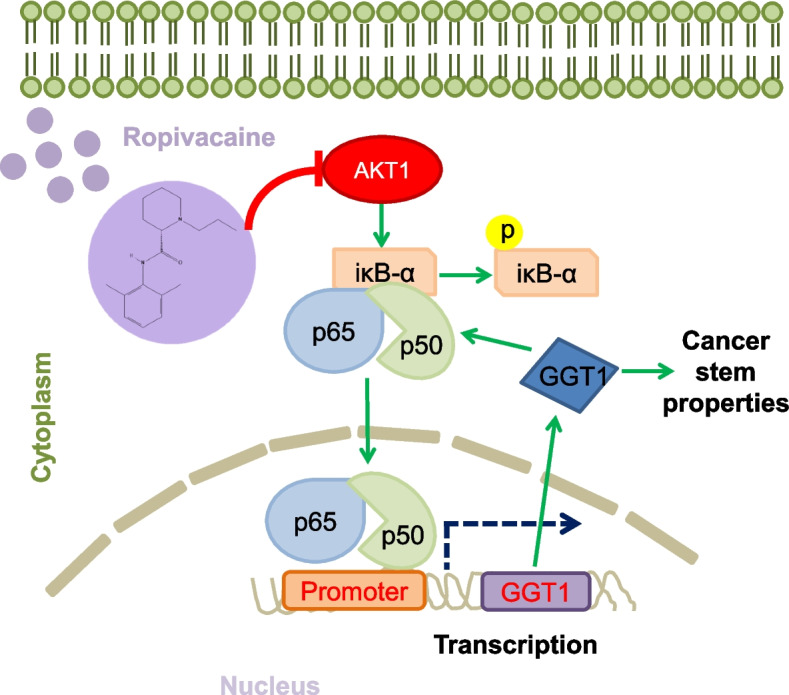
Schematic diagram of ropivacaine inhibiting the CSC-like traits of breast cancer cells. Ropivacaine targets AKT1 protein and inhibits its kinase activity, thereby blocking the NF-κB/GGT1 positive feedback loop and ultimately suppressing the CSC-like traits of breast cancer cells

In conclusion, our work suggests that ropivacaine functions as an AKT1-specific inhibitor, suppressing CSC-like phenotypes in breast cancer cells by inhibiting the NF-κB/GGT1 positive feedback loop (Fig. 11). Therefore, our findings provide valuable insights into the pharmacological effects of ropivacaine on breast cancer cells and offer a novel clinical consideration for the selection of intravenous anesthetics for breast cancer patients during surgery.

### Supplementary Information

**Supplementary Material 1.**

**Supplementary Material 2.**

## Data Availability

All data in our study that support the findings are available from the corresponding authors upon reasonable request.

## Abbreviations

CSCs: Cancer stem cells
GGT1: Gamma-glutamyltransferase 1
AKT1: AKT serine/threonine kinase 1
KEGG: Kyoto Encyclopedia of Genes and Genomes
GSEA: Gene set enrichment analysis
NF-κB: Nuclear factor kappa B
ChIP: Chromatin immunoprecipitation
IHC: Immunohistochemistry
TCGA: The Cancer Genome Atlas
SOX2: SRY-box transcription factor 2
CETSA: Cellular thermal shift assay
