# Supplementary material for: Ropivacaine as a novel AKT1 specific inhibitor regulates the stemness of breast cancer
Source: J Exp Clin Cancer Res. 2024 Mar 25;43:90. doi: 10.1186/s13046-024-03016-9 (PMC10962119; doi:10.1186/s13046-024-03016-9)
Supplement: Supplementary file 1 — Supplementary Material 1. [file 13046_2024_3016_MOESM1_ESM.docx]

**Supplemental Table 1. Antibodies.**

| Antibodies | Assay | Origin | Dilution |
| --- | --- | --- | --- |
| CD24 | FACS | Biolengend 311105 | 1:500 |
| CD44 | FACS | Biolengend 338805 | 1:500 |
| CD44 | IF | Proteintech 15675-1-AP | 1:500 |
| p-NF-κB | WB | CST  3033 | 1:1000 |
| NF-κB | WB/ChIP | CST  8242 | 1:1000/1:100 |
| p-iκBa | WB | CST  2859 | 1:1000 |
| iκBa | WB | CST  4812 | 1:1000 |
| β-actin | WB | Proteintech  66009-1-Ig | 1:1000 |
| CD133 | WB | Proteintech 18470-1-AP | 1:1000 |
| OCT4 | WB | Proteintech 11263-1-AP | 1:1000 |
| SOX2 | WB | Proteintech 11064-1-AP | 1:1000 |
| GGT1 | WB/IF | Abcam ab55138 | 1:1000/1:500 |
| p-AKT1 | WB | Proteintech  66444-1-Ig | 1:1000 |
| AKT1 | WB | Proteintech  60203-2-Ig | 1:1000 |

**Supplemental Table 2. Sequences of siRNAs, shRNAs, and primers for cloning, ChIP assays and qRT-PCR.**

| Gene | Application | Sequence | Species |
| --- | --- | --- | --- |
| siGGT1-1 | Cell transfection | CCTCTTCCTCACCATCTACAA | Human |
| siGGT1-2 | Cell transfection | TGGTTGTCAGGTTCCTTGG | Human |
| shGGT1-1-F | plasmid  construction | ccggCCTCTTCCTCACCATCTACAAggatccTTGTAGATGGTGAGGAAGAGGtttttg | Human |
| shGGT1-1-R | plasmid  construction | aattcaaaaaCCTCTTCCTCACCATCTACAAggatccTTGTAGATGGTGAGGAAGAGG | Human |
| shGGT1-2-F | plasmid  construction | ccggTGGTTGTCAGGTTCCTTGGggatccCCAAGGAACCTGACAACCAtttttg | Human |
| shGGT1-2-R | plasmid  construction | aattcaaaaaTGGTTGTCAGGTTCCTTGGggatccCCAAGGAACCTGACAACCA | Human |
| ShCD44-1-F | plasmid  construction | ccggTGTAACACCTACACCATTATCggatccGATAATGGTGTAGGTGTTACAtttttg | Human |
| ShCD44-1-R | plasmid  construction | aattcaaaaaTGTAACACCTACACCATTATCggatccGATAATGGTGTAGGTGTTACA | Human |
| ShCD44-2-F | plasmid  construction | ccggATGCAATGTGCTACTGATTGT ggatccACAATCAGTAGCACATTGCATtttttg | Human |
| ShCD44-2-R | plasmid  construction | aattcaaaaaATGCAATGTGCTACTGATTGTggatccACAATCAGTAGCACATTGCAT | Human |
| GGT1 promoter-clone-F | plasmid  construction | CTAGCTAGCCCGCTTGCATCTGGACCG | Human |
| GGT1 promoter-clone-R | plasmid  construction | CCGCTCGAGCCAAGCTAAGATTCCCTGCTGT | Human |
| GGT1 promoter -F | CHIP- PCR | AAGGGTGATAAGGAGATGA | Human |
| GGT1 promoter -R | CHIP- PCR | GATTGGGGTGCTAGGTAGA | Human |
| GGT1-F | qRT-PCR | CTGGGGAGATCCGAGGCTAT | Human |
| GGT1-R | qRT-PCR | GATGACGGTCCGCTTGTTTTC | Human |
| β-actin-F | qRT-PCR | GGCACCCAGCACAATGAAG | Human |
| β-actin-R | qRT-PCR | CCGATCCACACGGAGTACTTG | Human |

**Supplementary Table 3.** Correlation of GGT1 expression with clinicopathological features in 70 breast cancer patients

| Expression of LINC00467 | | | |
| --- | --- | --- | --- |
| Variables Low (%) High (%) P value | | | |
| Age |  |  | 0.7175 |
| <50 | 14(48.28%) | 15(51.72%) |  |
| ≥50 | 18(43.90%) | 23(56.10%) |  |
| Triple-negative |  |  | 0.8580 |
| No | 15(46.88%) | 17(53.13%) |  |
| Yes | 17(44.74%) | 21(55.26%) |  |
| TNM staging |  |  | 0.0258* |
| Ⅰ-Ⅱ | 22(57.89%) | 16(42.11%) |  |
| Ⅲ-Ⅳ | 10(31.25%) | 22(68.75%) |  |
| Tumor size (cm) |  |  | 0.2382 |
| <5 | 18(52.94%) | 16(47.06%) |  |
| ≥5 | 14(38.89%) | 22(61.11%) |  |
| Lymph node metastasis |  |  | 0.0266* |
| Positive | 7(28.00%) | 18(72.00%) |  |
| Negative | 25(55.56%) | 20(44.44%) |  |


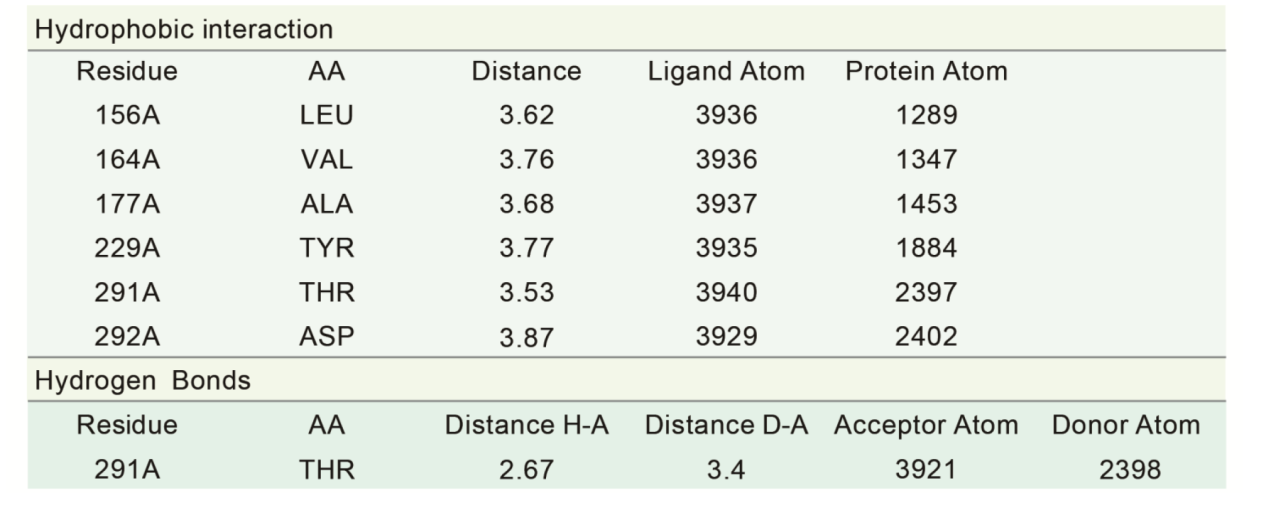


**Supplementary Fig. S1. Interaction details of docking pose between ropivacaine hydrochloride and AKT1 kinase**


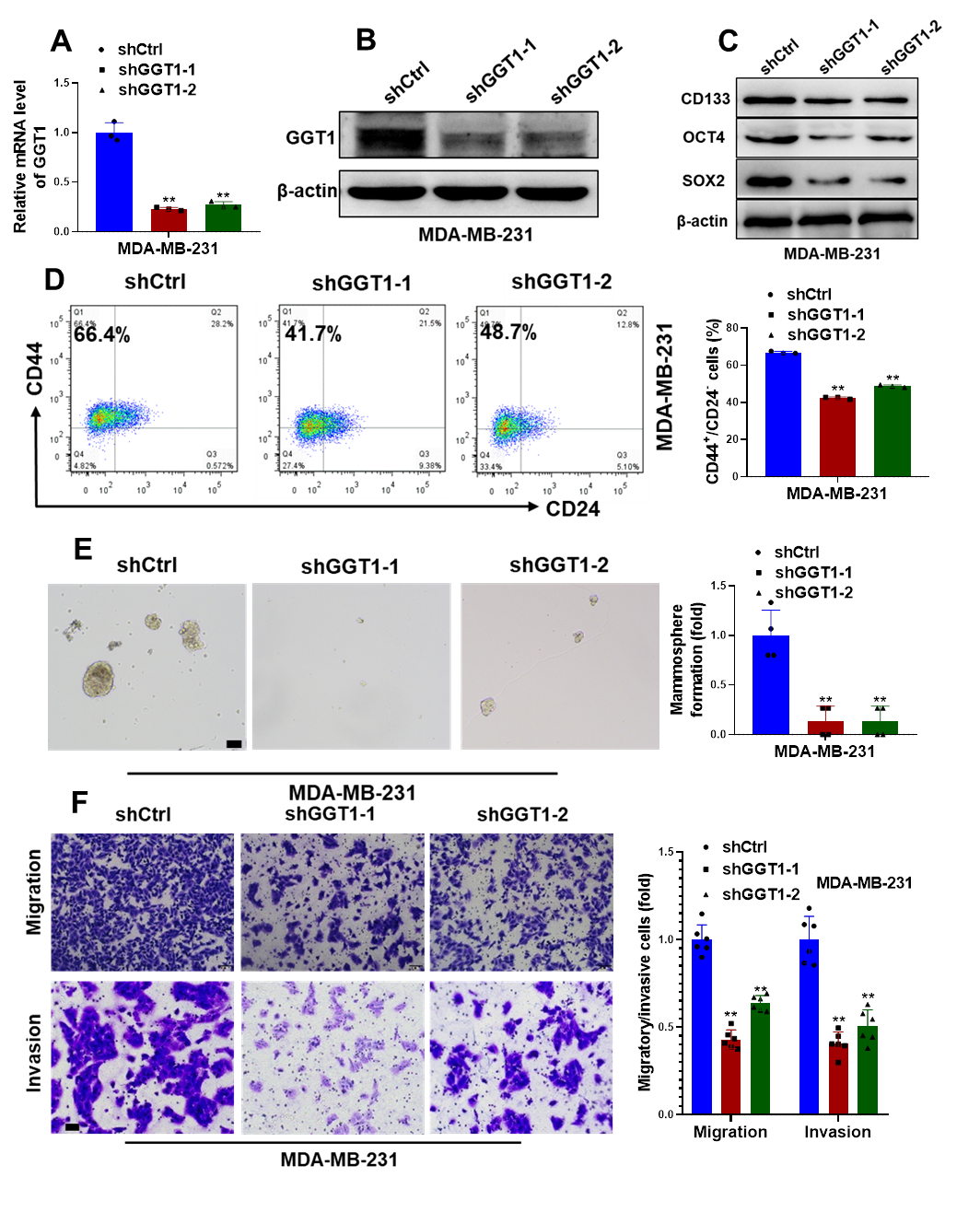


**Supplementary Fig. S2. Effects of GGT1 on CSCs-like phenotypes of MDA-MB-231 cells *in vitro*. (A-B)** RNA **(A)** and protein **(B)** level of GGT1 in MDA-MB-231 cells stably transfected with shRNAs against GGT1 (shGGT1-1 and shGGT1-2) or negative control (shCtrl) were determined by qRT-PCR and western blot, respectively. **(C)** Stem cell markers (CD133, OCT4, SOX2) in GGT1-depleting MDA-MB-231 cells were analyzed by western blot. **(D)** CD44^+^/CD24^−^ subpopulation in GGT1-depleting MDA-MB-231 cells was measured by FACS analysis. **(E)** Mammosphere formation of GGT1-depleting MDA-MB-231 cells (scale bar = 100 μm). **(F)** Migration and invasion of GGT1-depleting MDA-MB-231 cells were examined by transwell assays (scale bar = 100 μm). Results are shown are shown as mean ± S.D from at least three independent experiments. **p* < 0.05; ***p* < 0.01; ****p* < 0.001 (unpaired two-tailed Student’s t test).


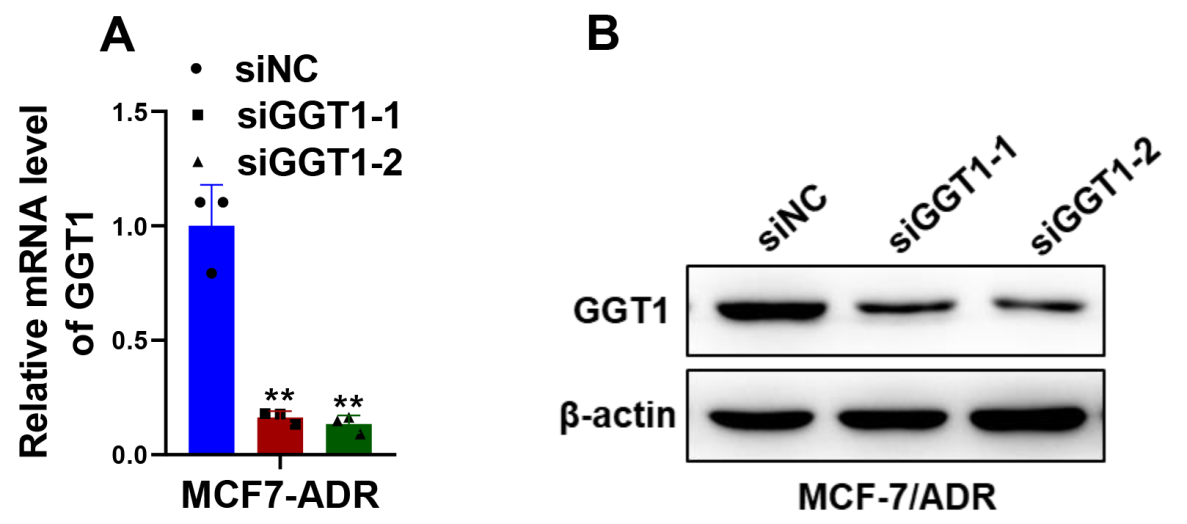


**Supplementary Fig. S3. Verification of GGT1 knochdown efficency in MCF-7/ADR cells. (A-B)** RNA **(A)** and protein **(B)** level of GGT1 in MCF-7/ADR cells transfected with siRNAs against GGT1 (siGGT1-1 and siGGT1-2) or negative control (siNC) were determined by qRT-PCR and western blot, respectively. Results are shown are shown as mean ± S.D from at least three independent experiments. **p* < 0.05; ***p* < 0.01; ****p* < 0.001 (unpaired two-tailed Student’s t test).


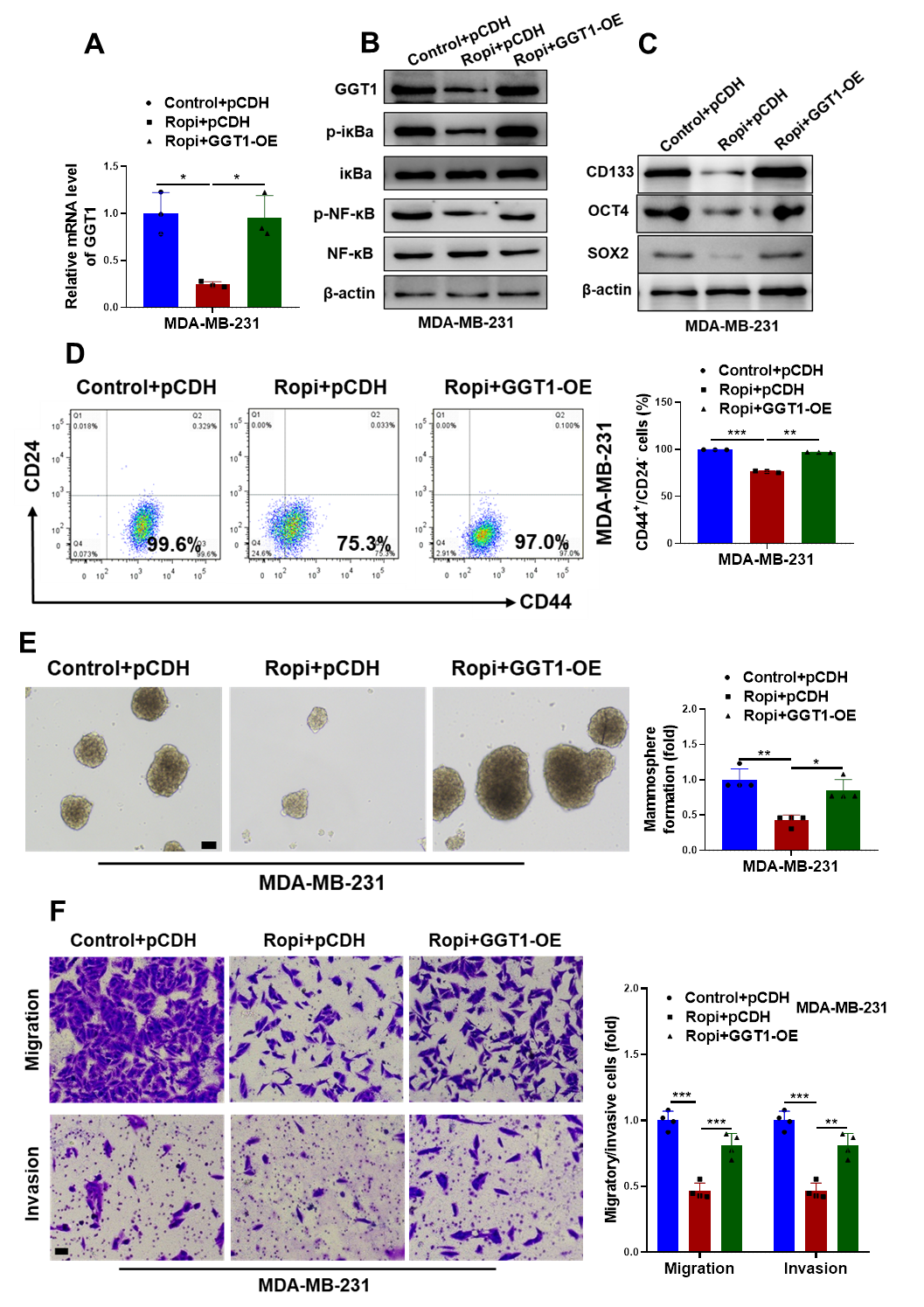


**Supplementary Fig. S4. The anti-CSCs effects of ropivacaine on MDA-MB-231 cells is dependent on NF-κB/GGT1 feedback loop.** MDA-MB-231 cells was transfected with GGT1 overexpression plasmid (GGT1-OE) or empty plasmid (Vector) and treated with ropivacaine (10 μM) or negative control for 48 h. **(A)** RNA level of GGT1 in the indicated treated-MDA-MB-231 cells was determined by qRT-PCR. **(B)** Protein levels of GGT1, p-iκBa, iκBa, p-NF-κB and NF-κB in the indicated treated-MDA-MB-231 cells were examined by western blot. **(C)** Stem cell markers (CD133, OCT4, SOX2) in the indicated treated-MDA-MB-231 cells were analyzed by western blot. **(D)** CD44^+^/CD24^−^ subpopulation in the indicated treated-MDA-MB-231 cells was measured by FACS analysis. **(E)** Mammosphere formation of the indicated treated-MDA-MB-231 cells (scale bar = 100 μm). **(F)** Migration and invasion of the indicated treated-MDA-MB-231 cells were examined by transwell assays (scale bar = 100 μm). Results are shown are shown as mean ± S.D from at least three independent experiments. **p* < 0.05; ***p* < 0.01; ****p* < 0.001 (unpaired two-tailed Student’s t test).
