# Supplementary material for: Ropivacaine as a novel AKT1 specific inhibitor regulates the stemness of breast cancer
Source: J Exp Clin Cancer Res. 2024 Mar 25;43:90. doi: 10.1186/s13046-024-03016-9 (PMC10962119; doi:10.1186/s13046-024-03016-9)

Fig.1E

CD133(lane 6~9)

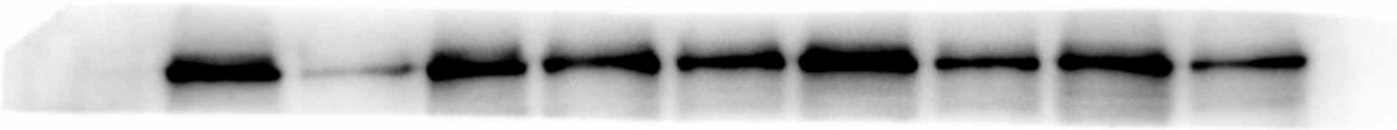

OCT4(lane 6~9)

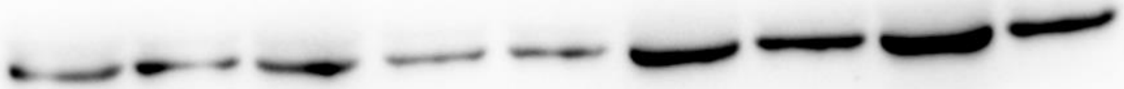

SOX2(lane 6~9)

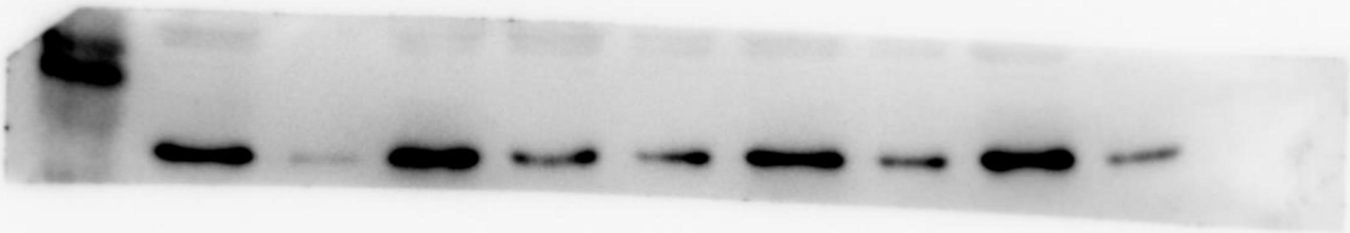

β-actin(lane 6~9)

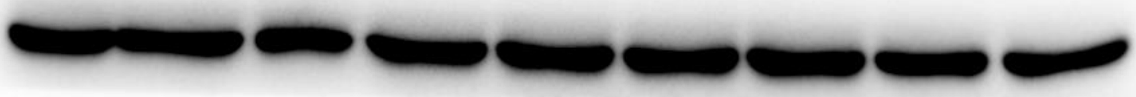

Fig.3G

GGT1(lane 1~2)

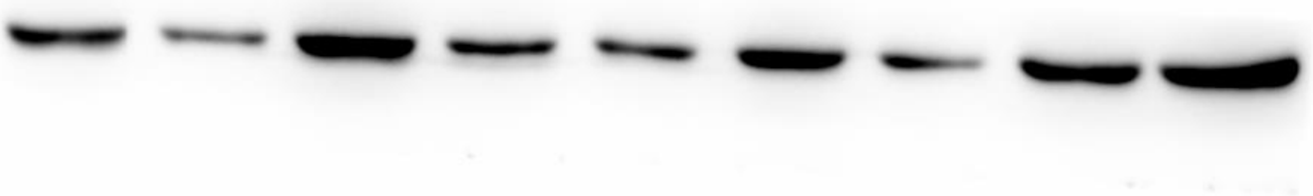

β-actin(lane 1~2)

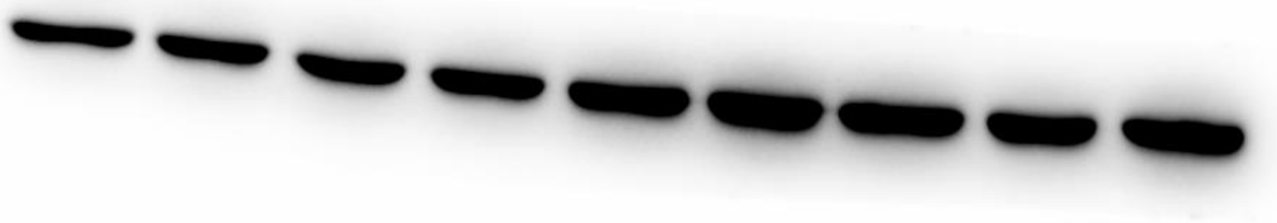

GGT1(lane 1~2)

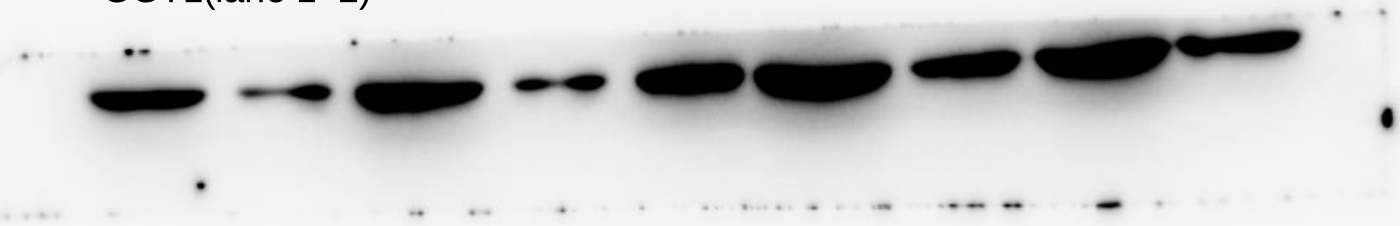

β-actin(lane 1~2)

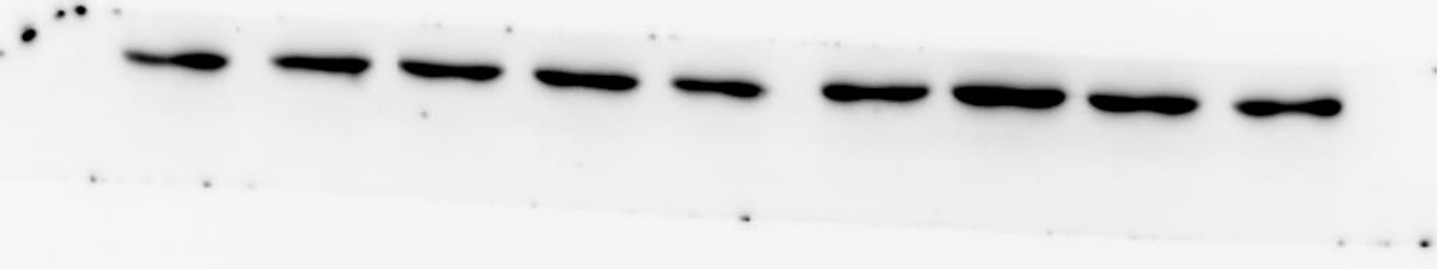

Fig.4A

P65 (lane 5~8)

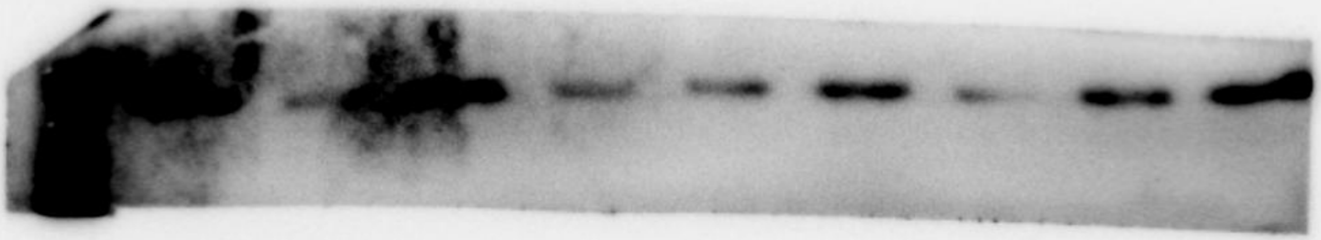

GGT1(lane 5~8)

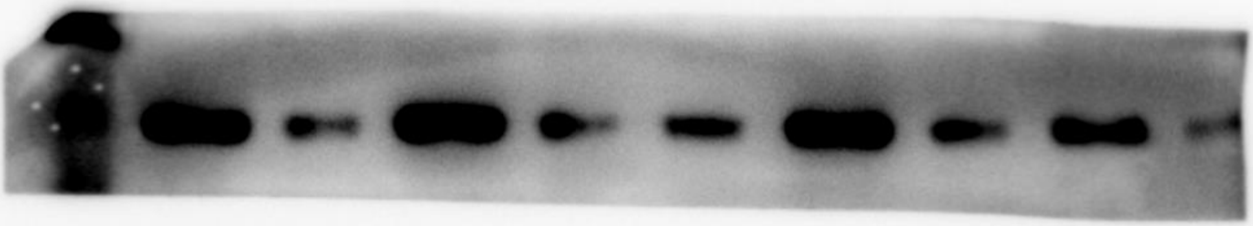

β-actin(lane 5~8)

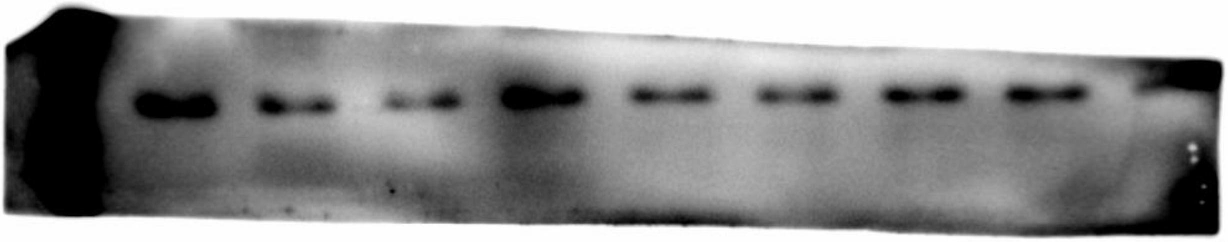

Fig.4C

P65 (lane 6~9)

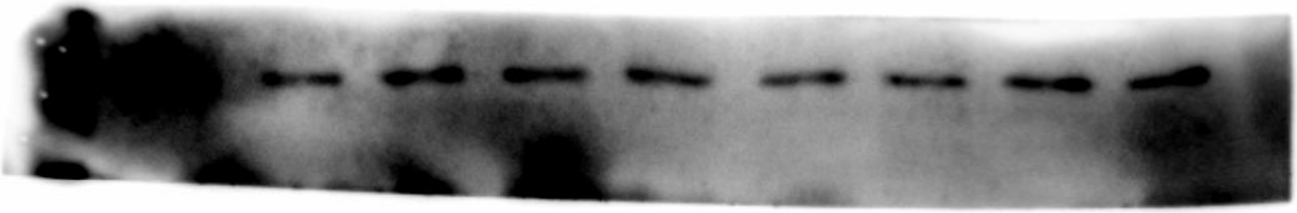

GGT1(lane 6~9)

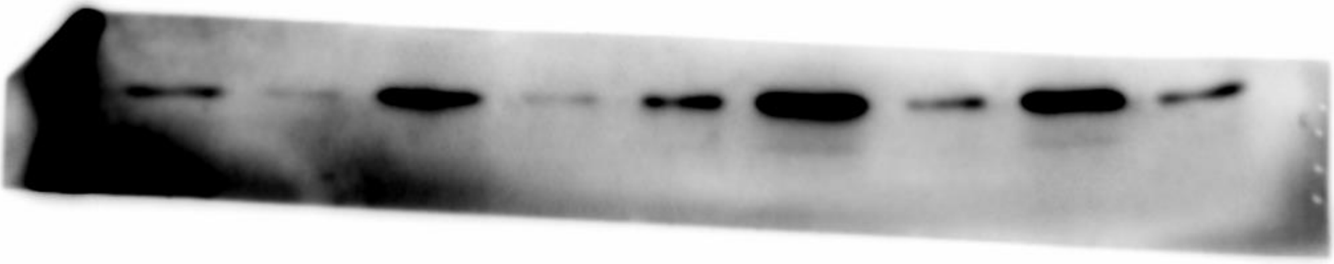

β-actin(lane 6~9)

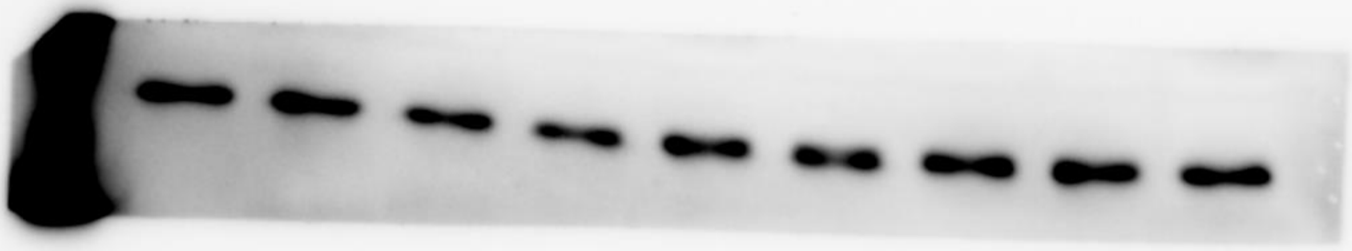

Fig.5B

AKT1 (lane 2~9)

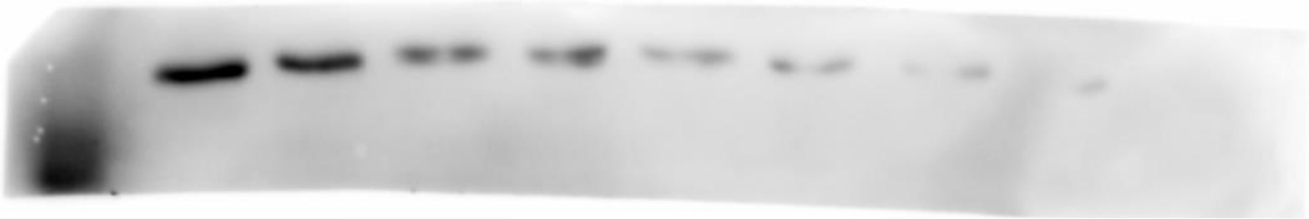

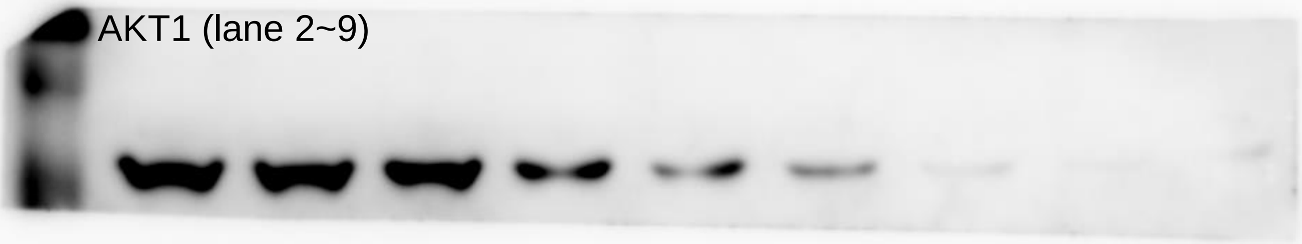

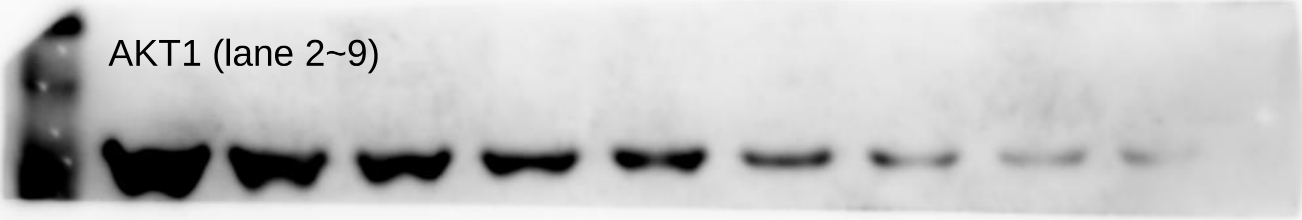

Fig.5C

AKT1 (lane 2~9)

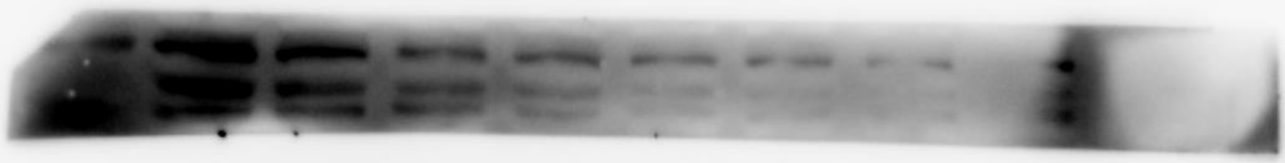

AKT1 (lane 2~9)

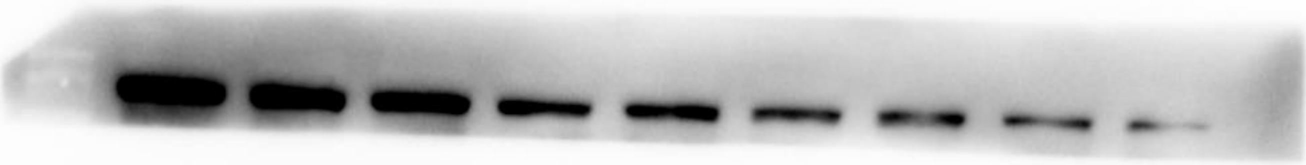

AKT1 (lane 2~9)

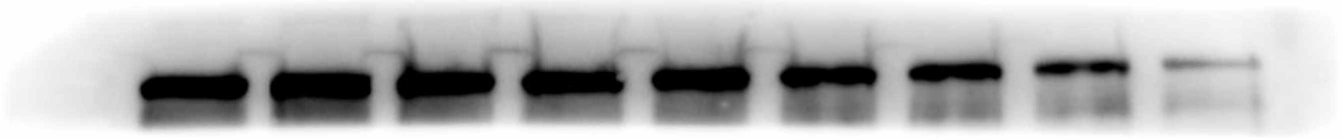

Fig.5E

AKT1 (lane 4~6)

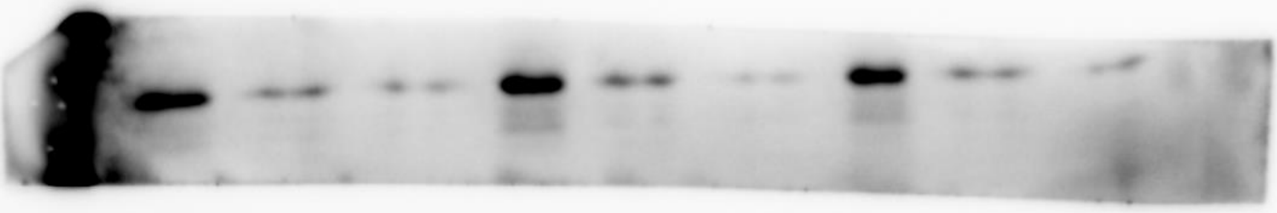

Fig.5F

p-AKT1 (lane 1~3, 7~9)

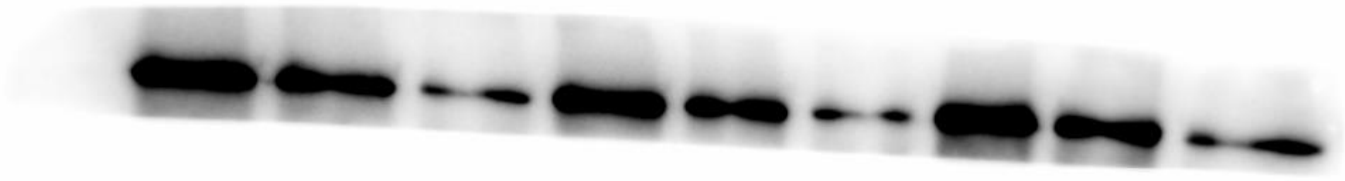

t-AKT1 (lane 4~9)

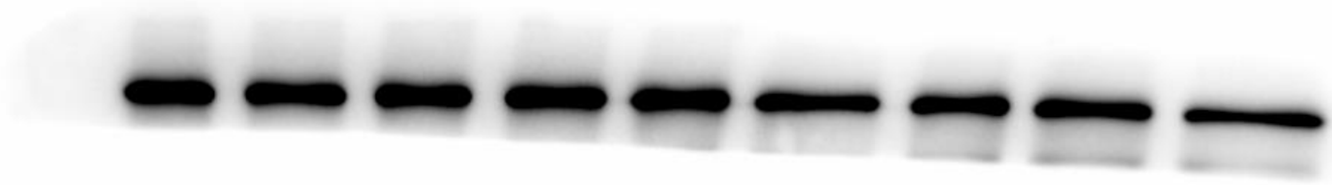

p-iκBa (lane 1~6)

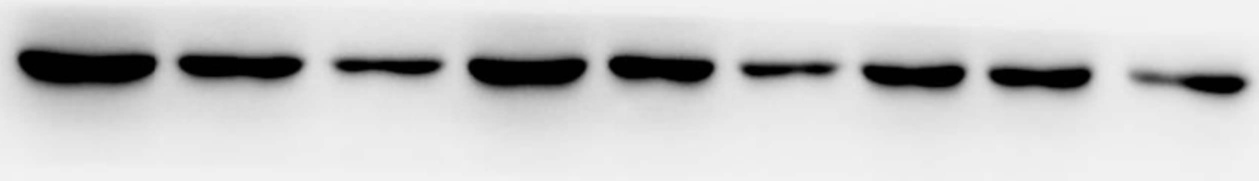

iκBa (lane 1~3, 7~9)

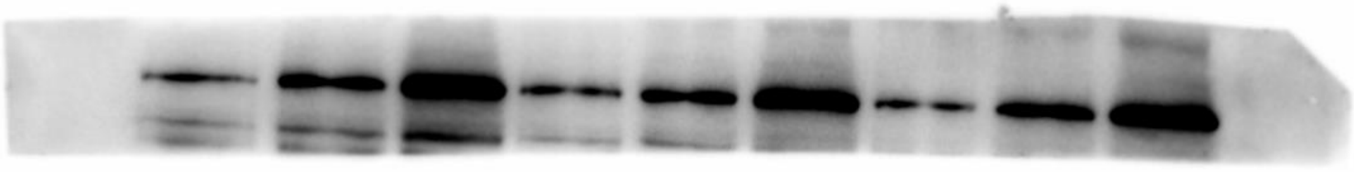

p-NF-κB (lane 4~9)

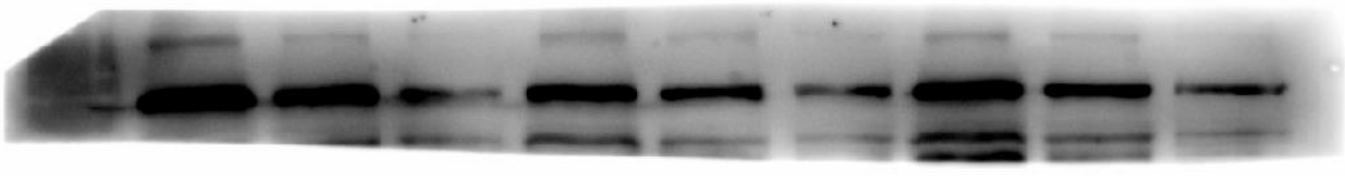

Fig.5F

NF-κB (lane 4~9)

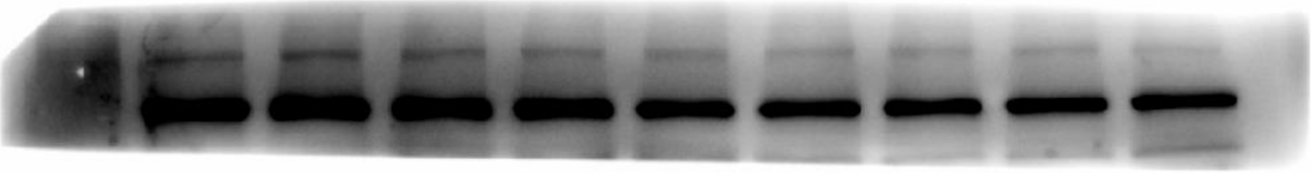

β-actin(lane 4~9)

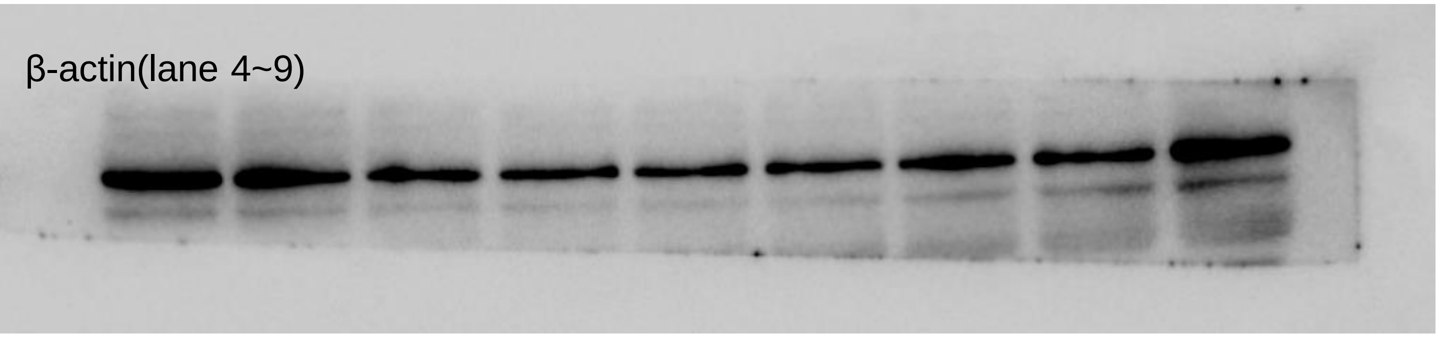

Fig.6C

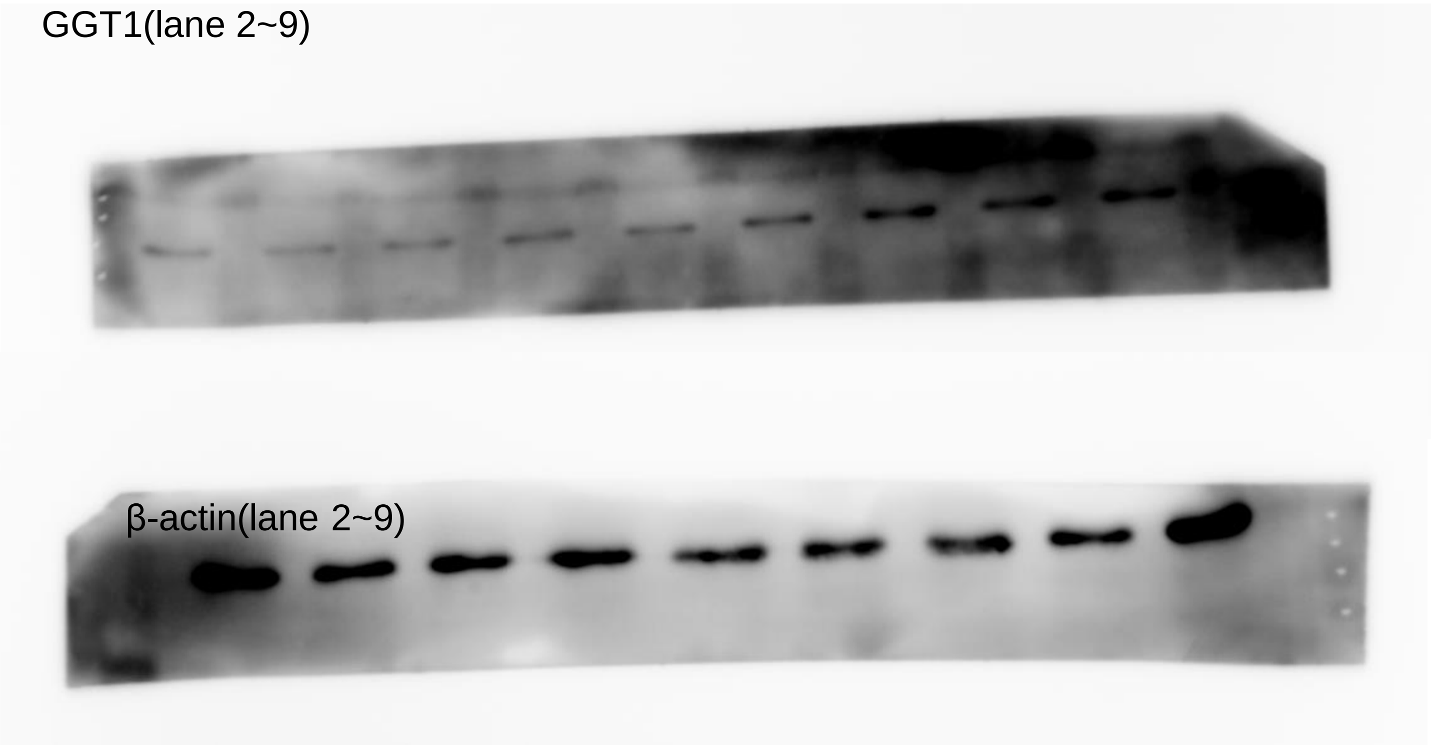

Fig.6D

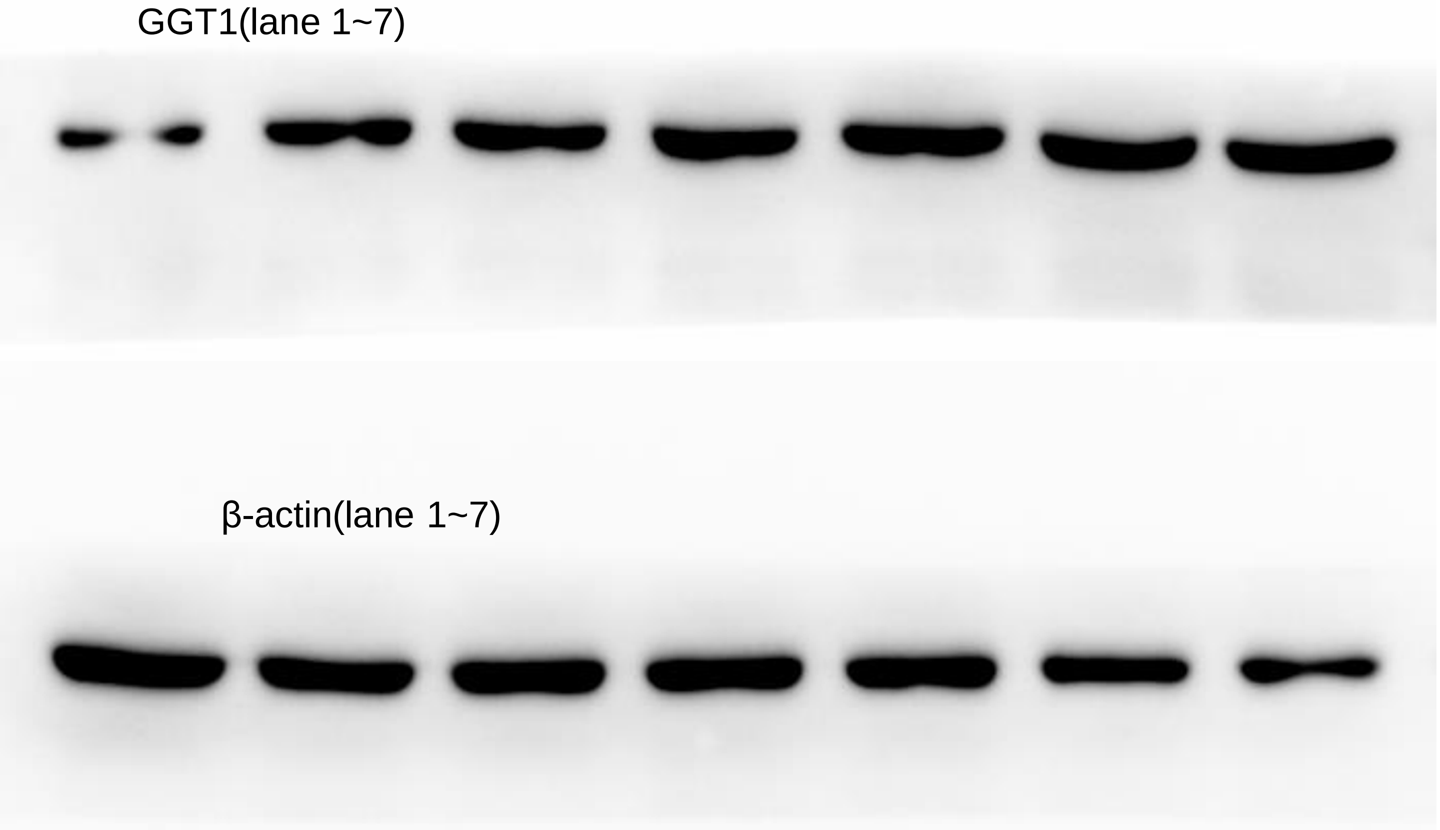

Fig.6E

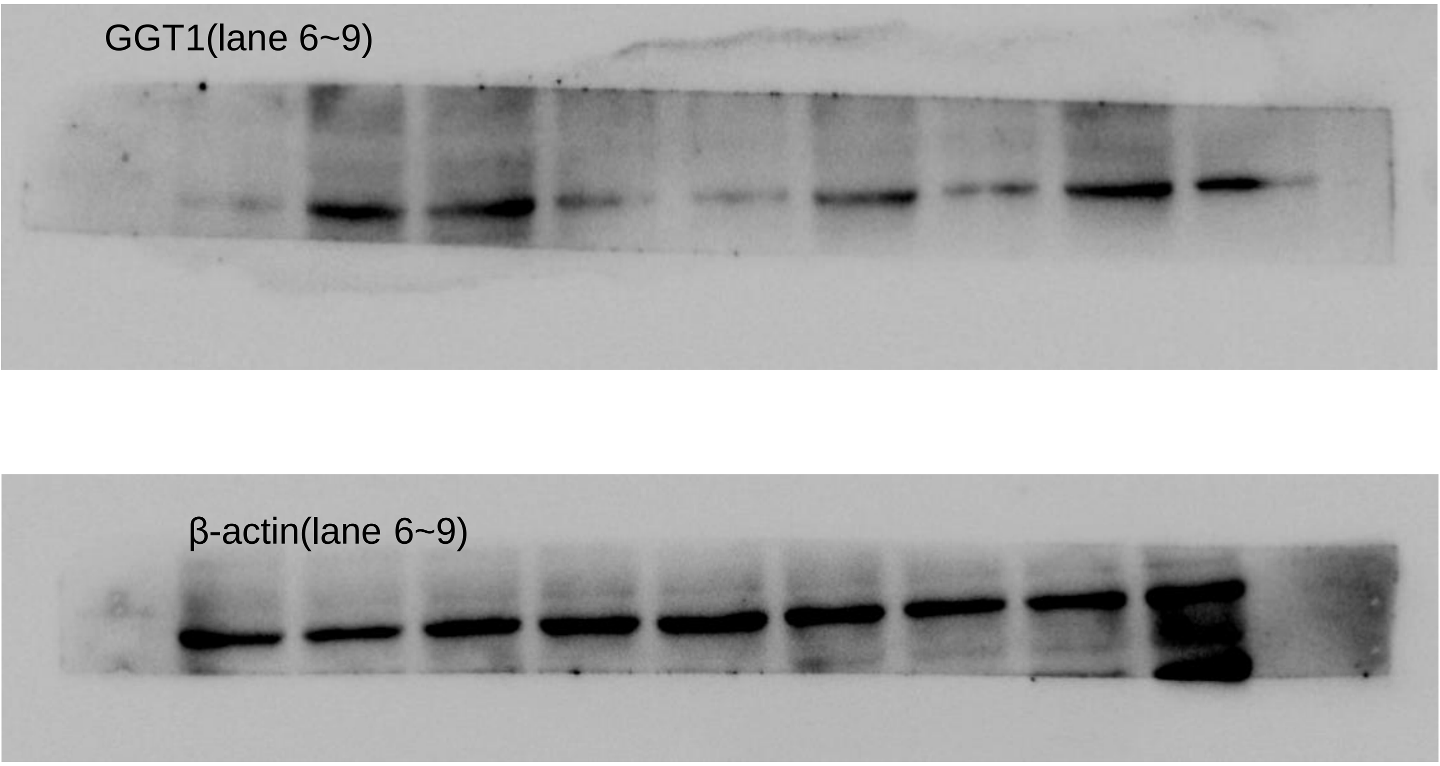

Fig.7B

GGT1(lane 3~5)

Fig.S2B

GGT1(lane 6~8)

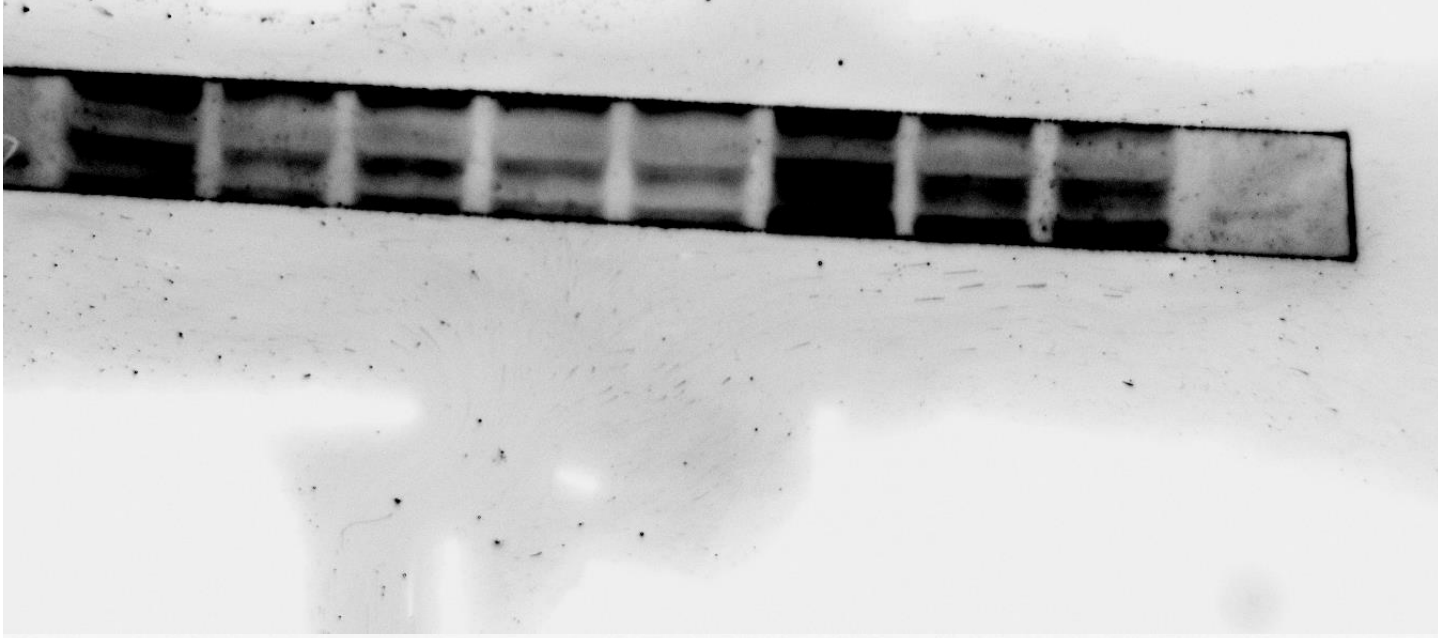

Fig.7B

β-actin(lane 3~5)

Fig.S2B

β-actin(lane 6~8)

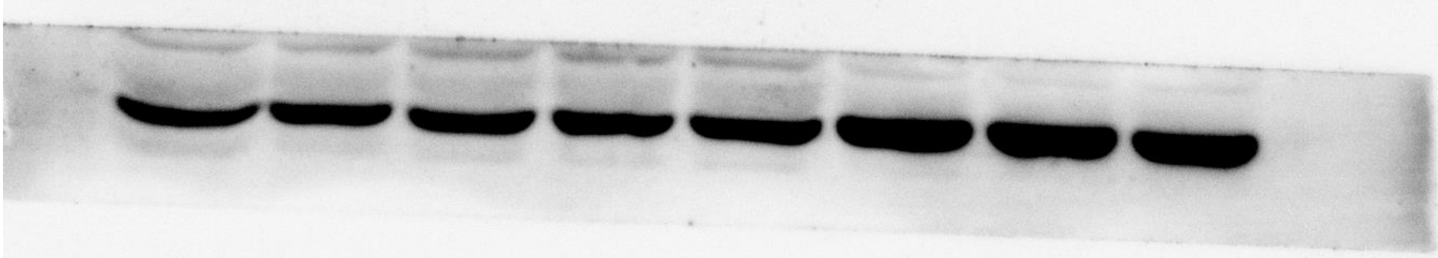

Fig.7C

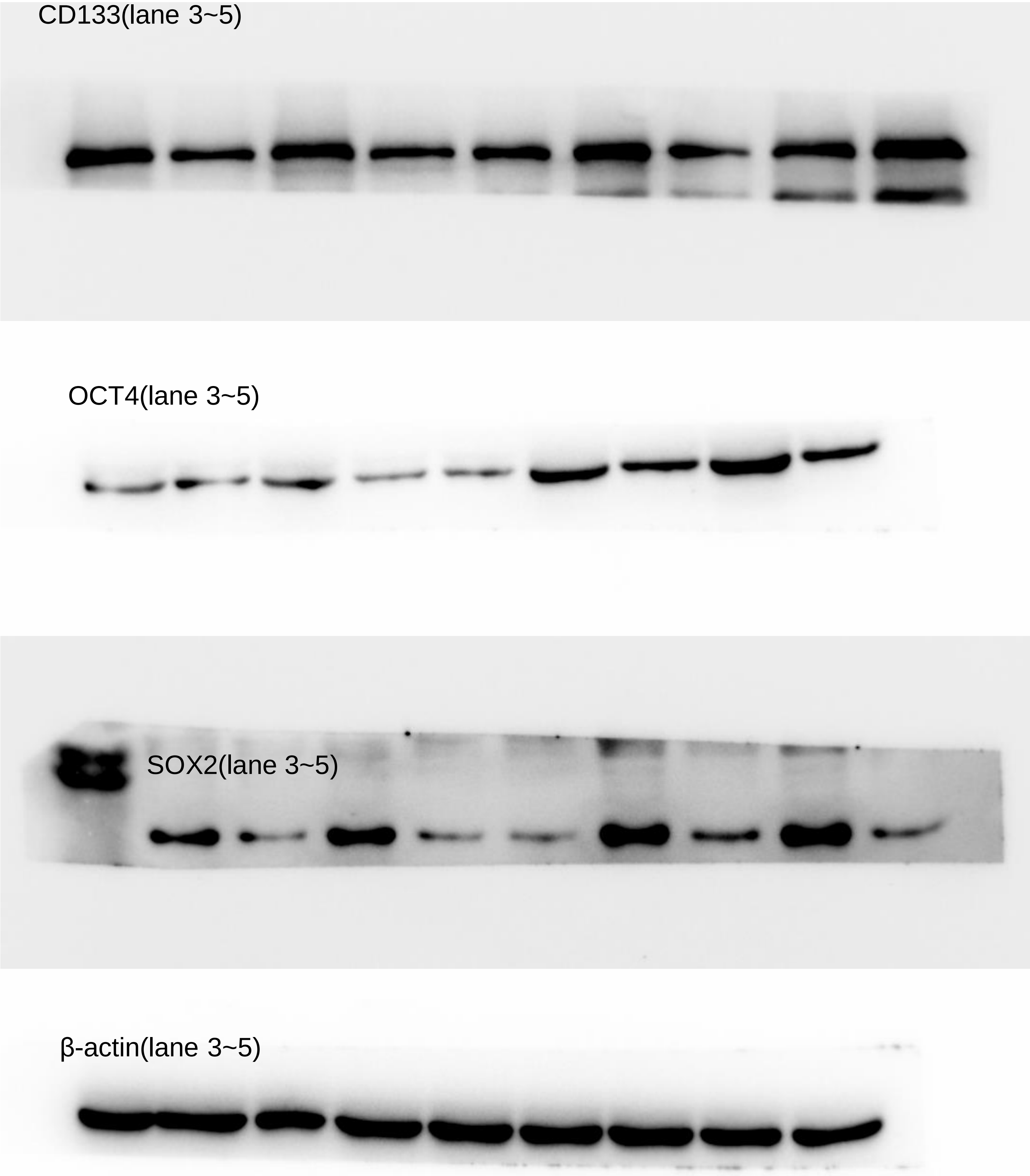

Fig.S2C

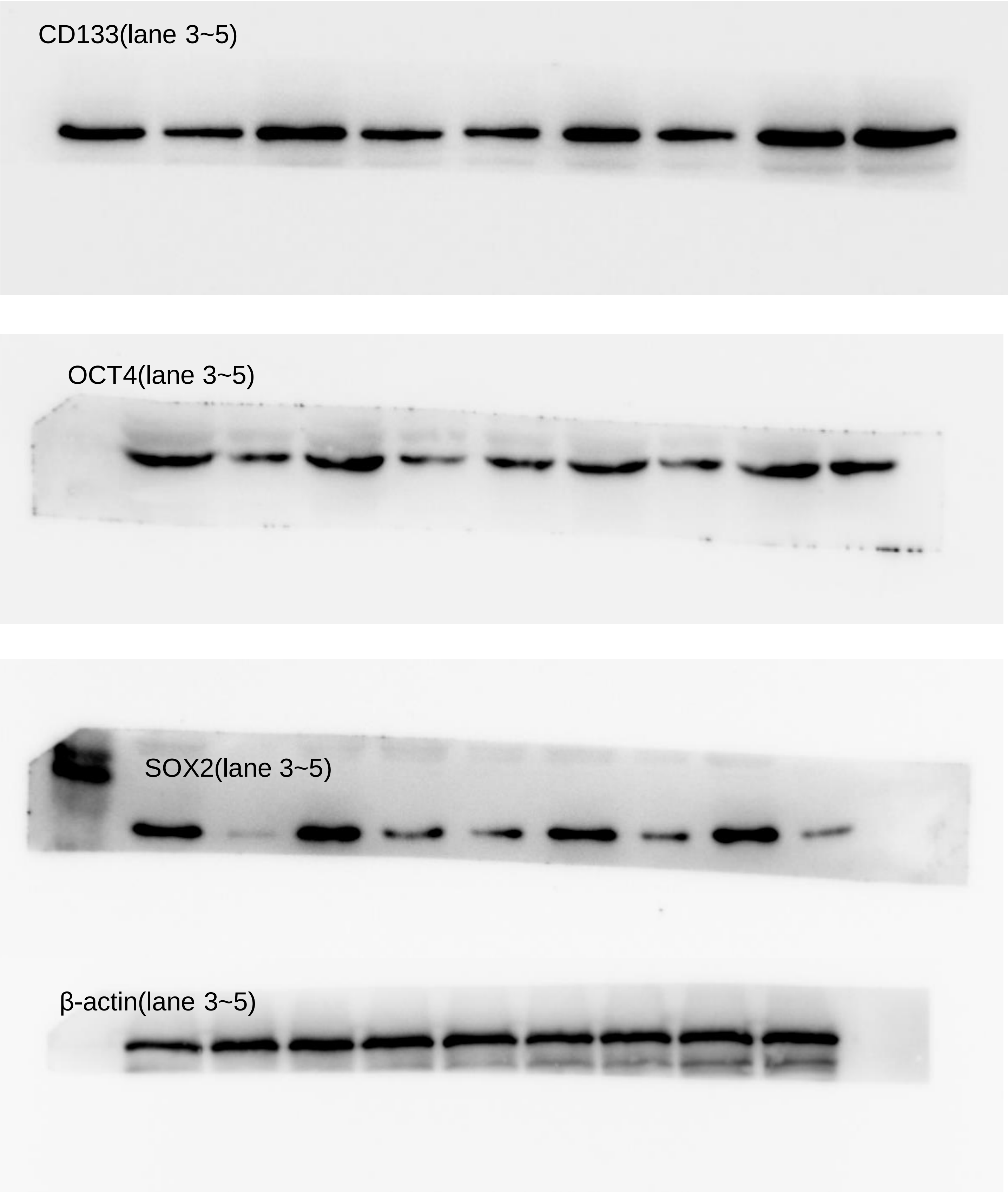

Fig.9G

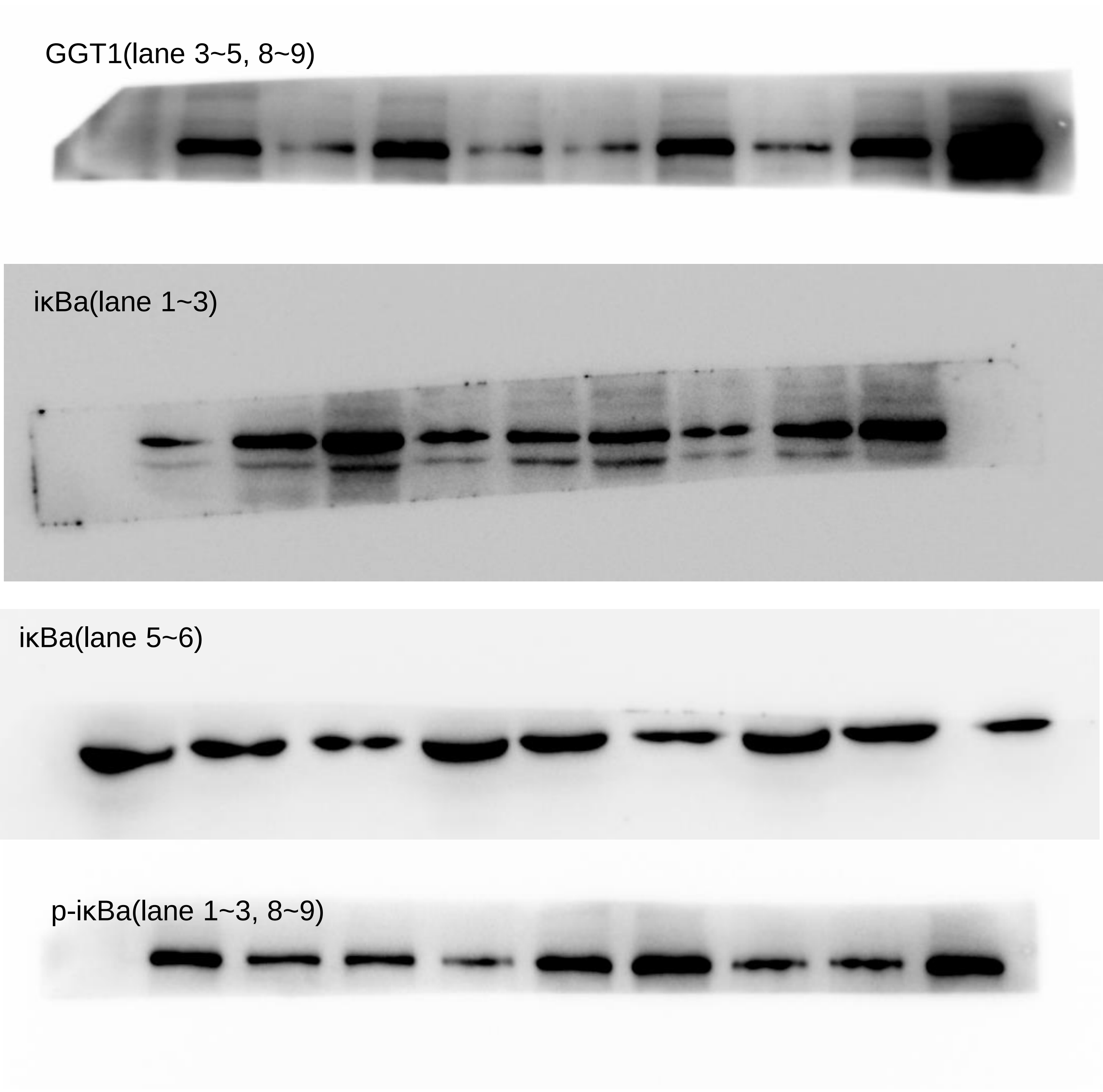

Fig.9G

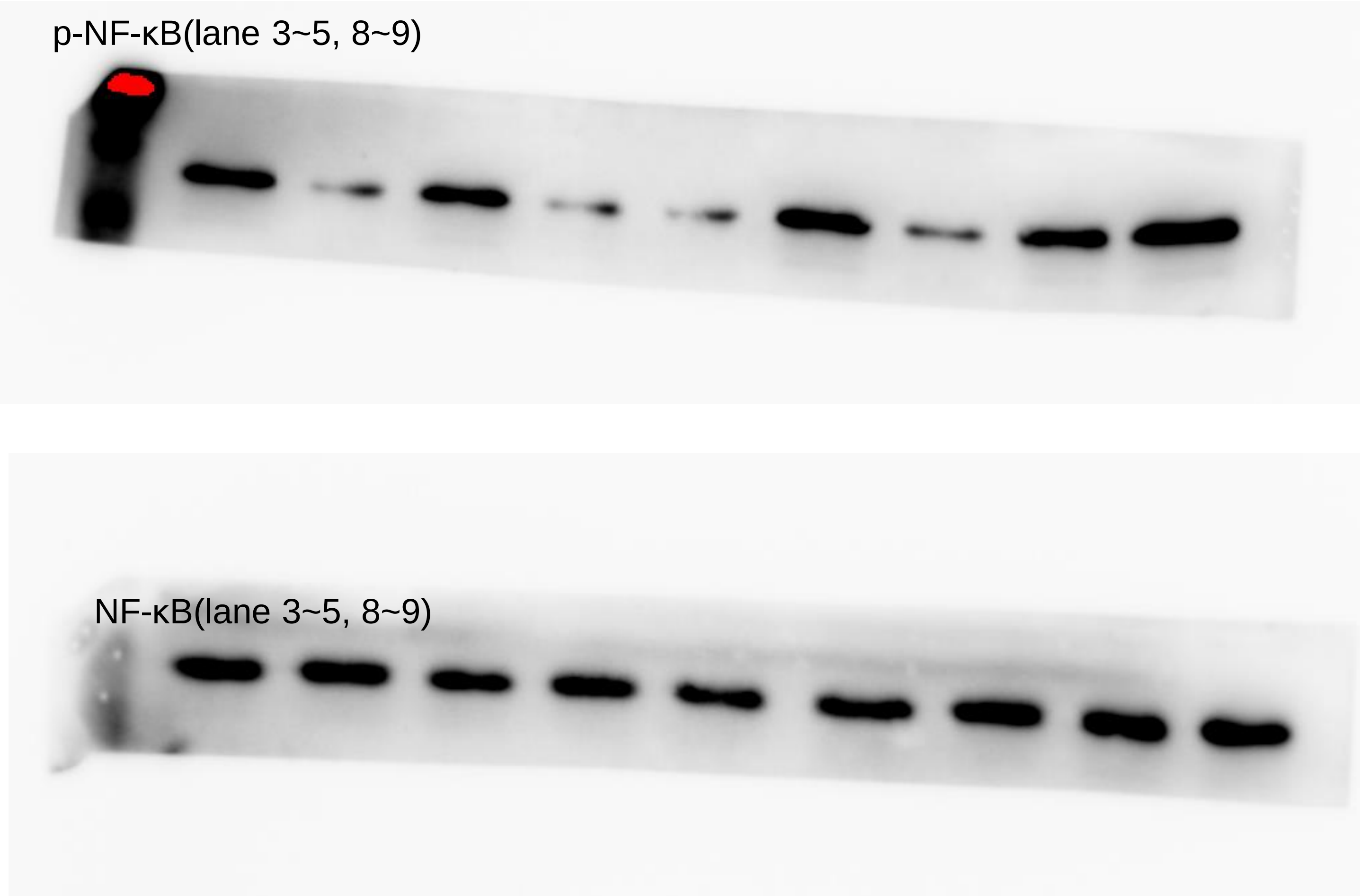

Fig.9H

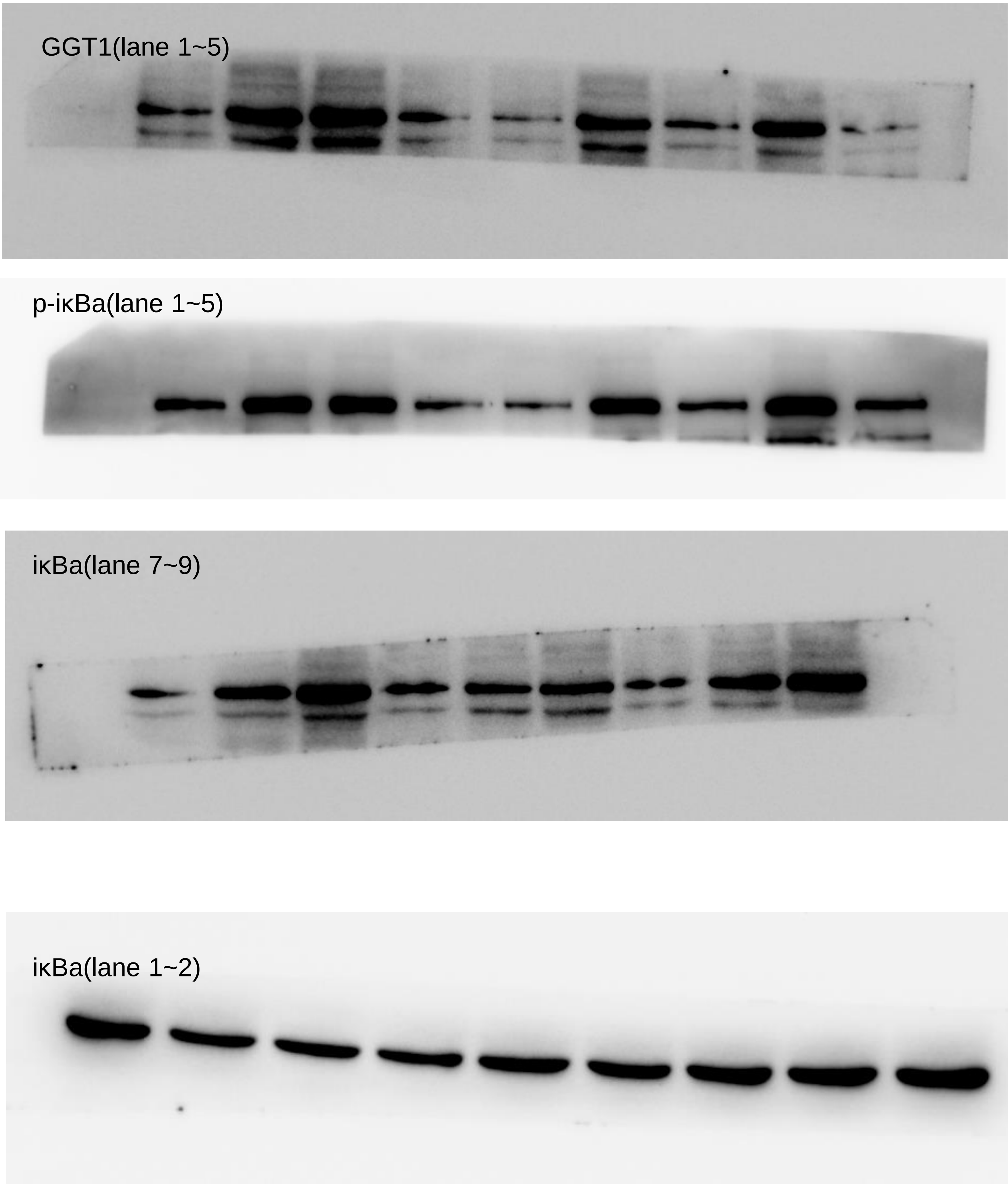

Fig.9H

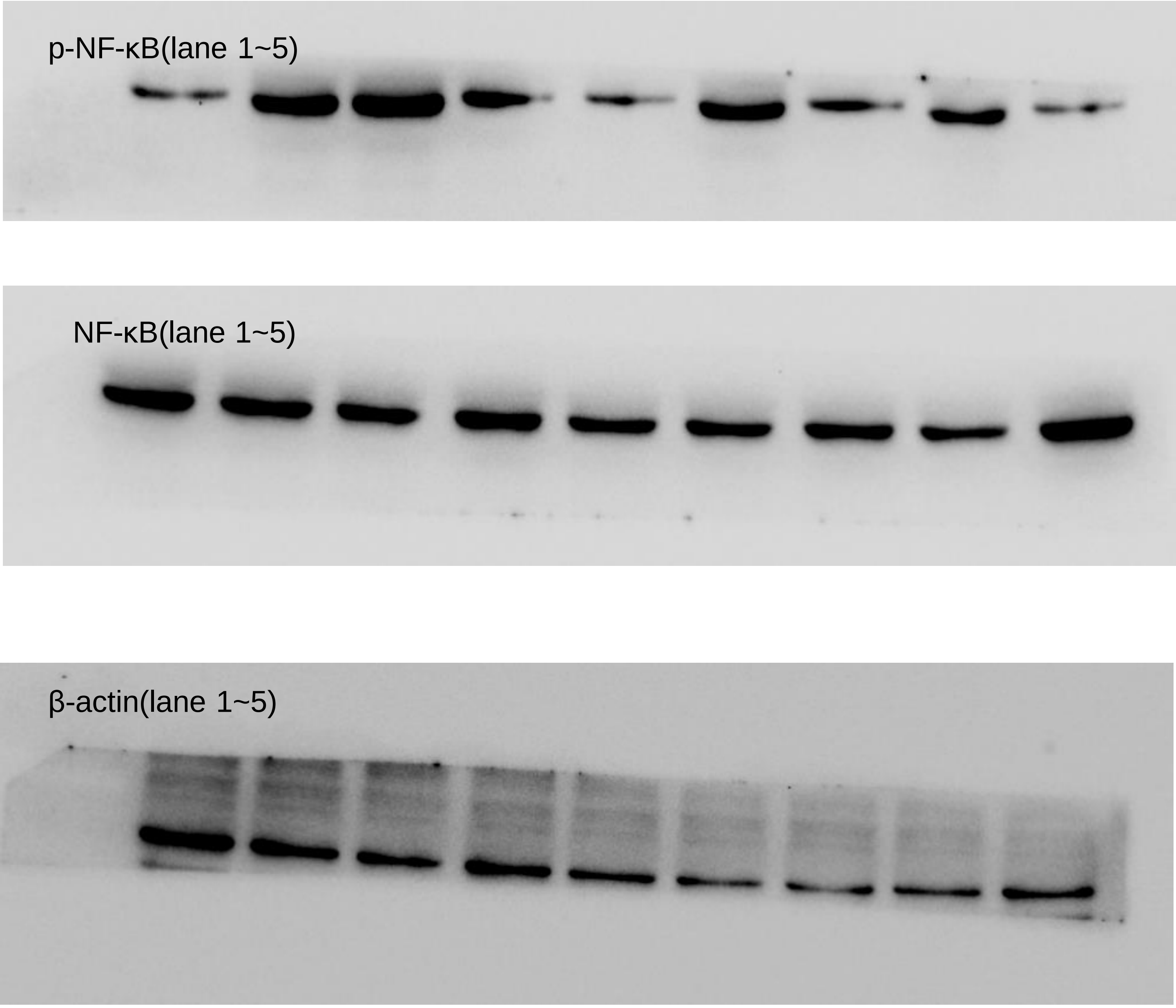

Fig.S3B

GGT1(lane 3~5)

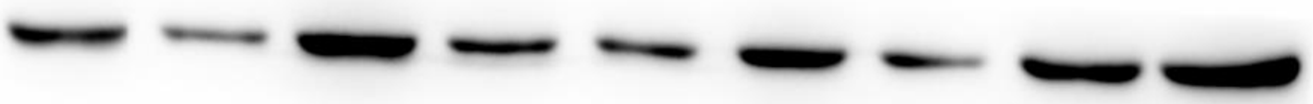

β-actin(lane 3~5)

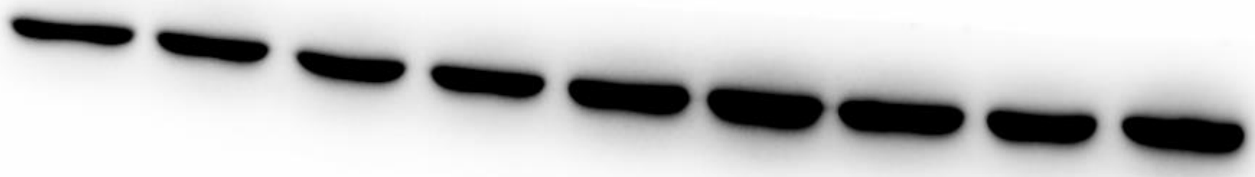

Fig.10B

GGT1(lane 6~8)

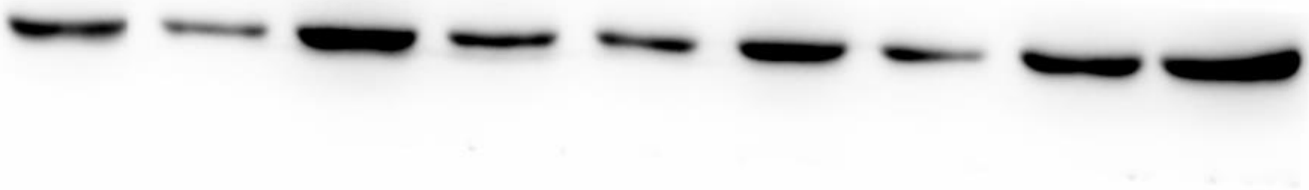

p-iκBa(lane 6~8)

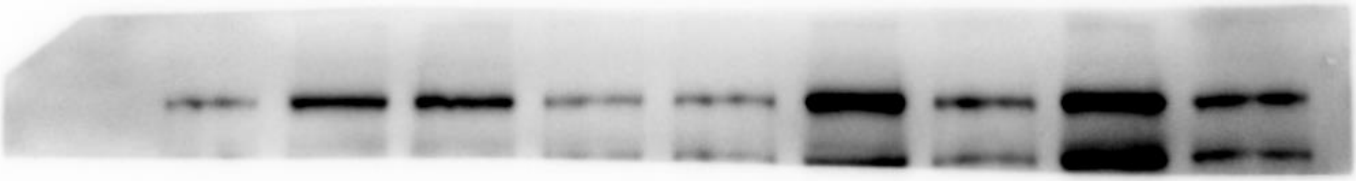

iκBa(lane 6~8)

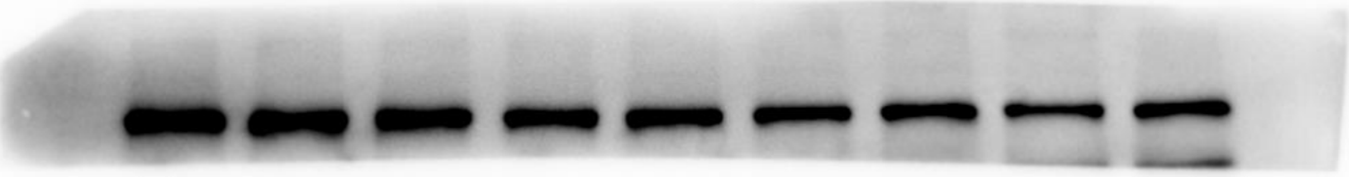

Fig.10B

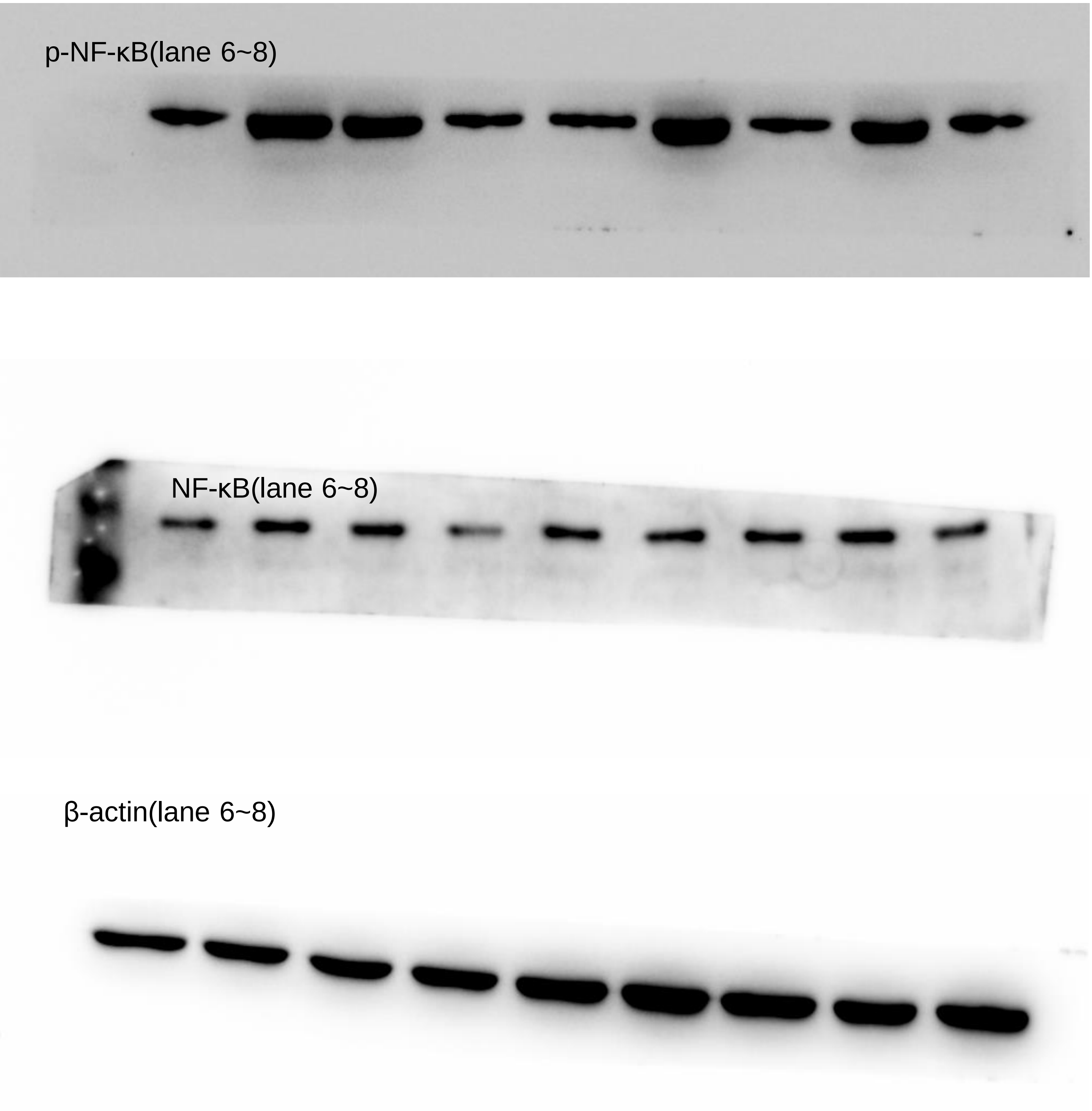

Fig.10C

CD133(lane 3~5)

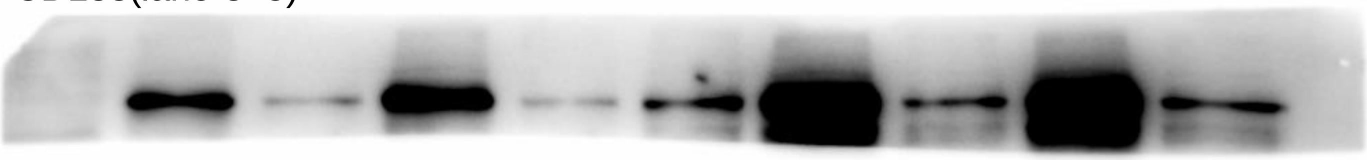

OCT4(lane 6~8)

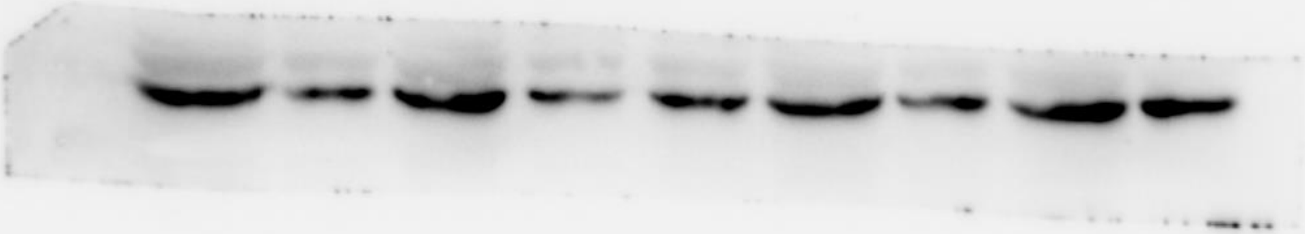

SOX2(lane 6~8)

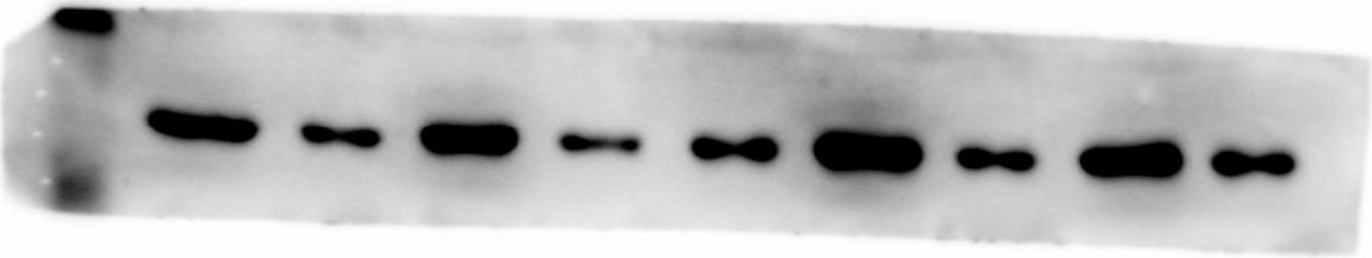

β-actin(lane 6~8)

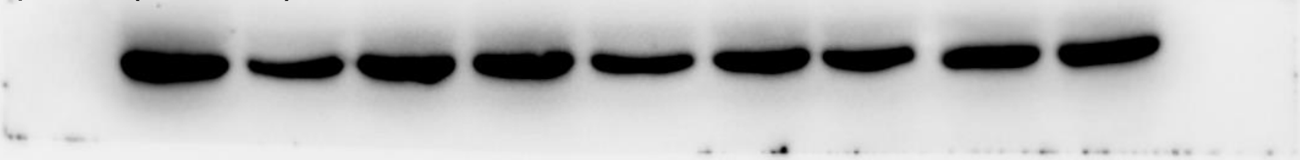

Fig.S4B

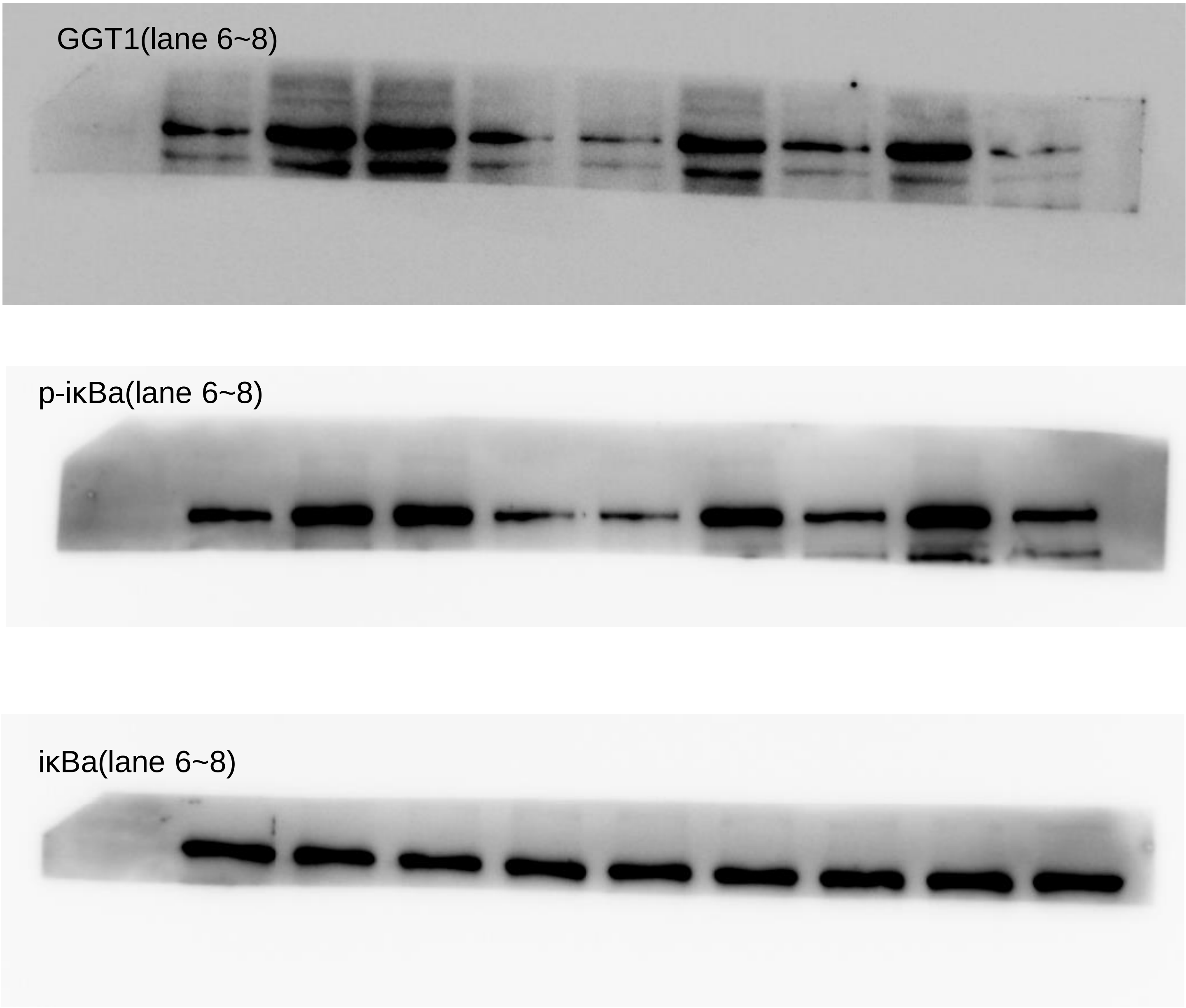

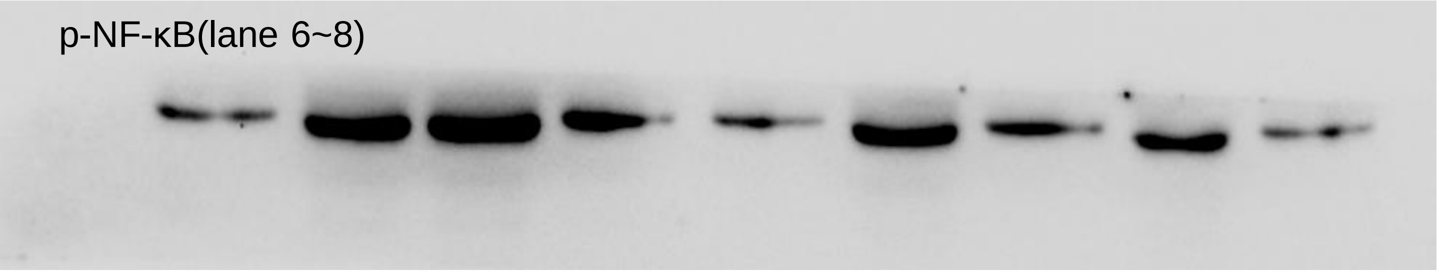
p-NF-κB(lane 6~8)

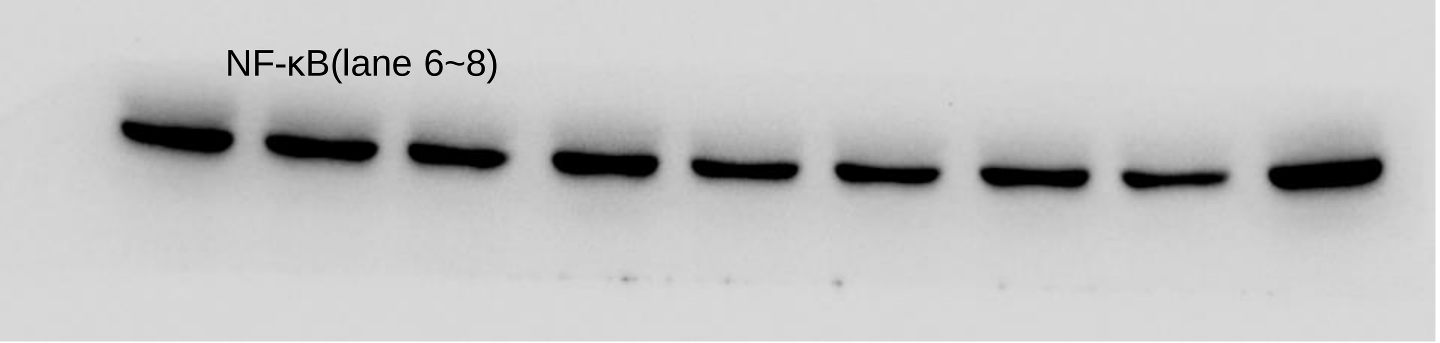
NF-κB(lane 6~8)

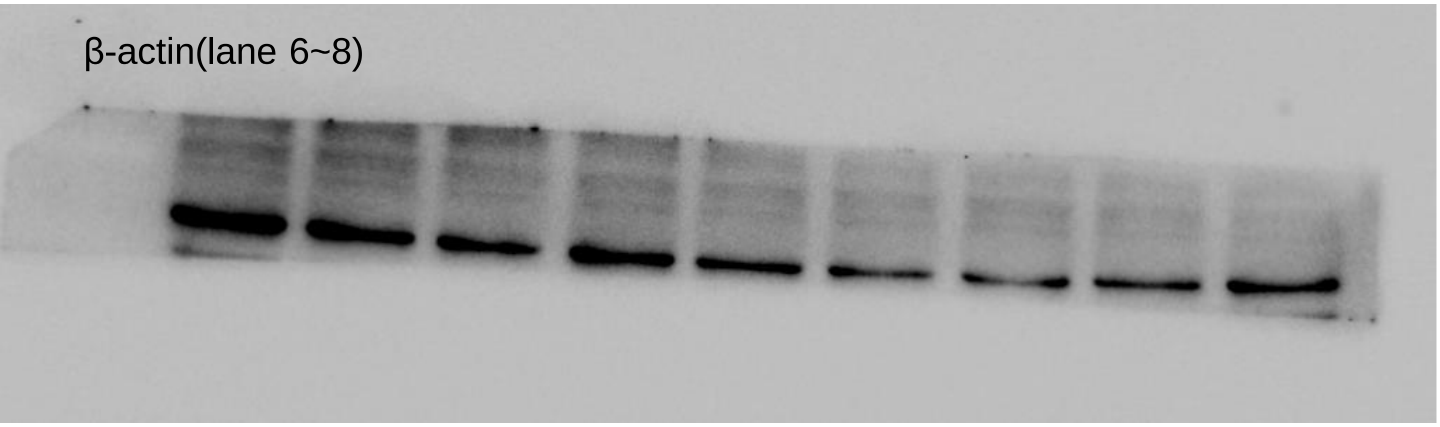
β-actin(lane 6~8)

Fig.S4C

CD133(lane 6~8)

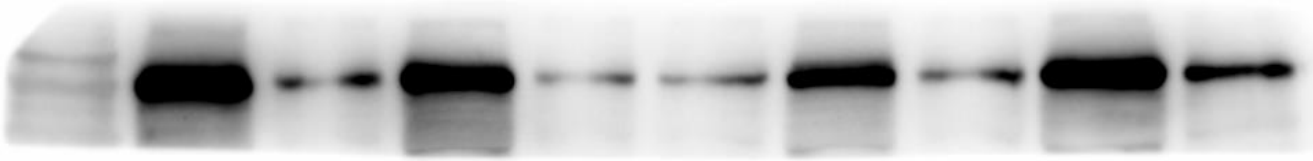

OCT4(lane 6~8)

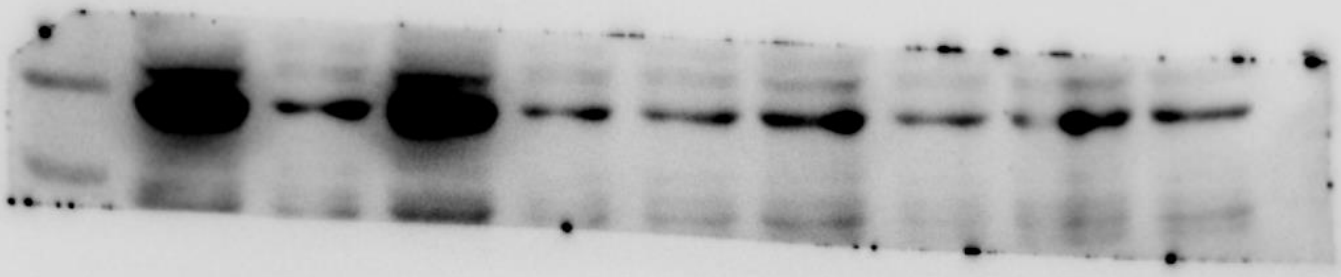

SOX2(lane 6~8)

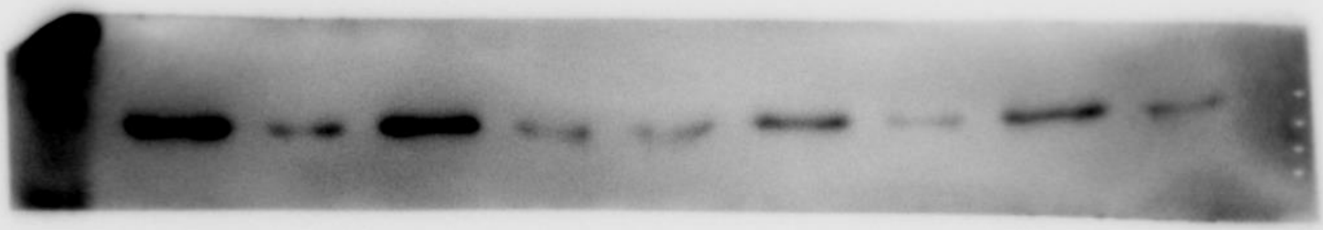

β-actin(lane 6~8)

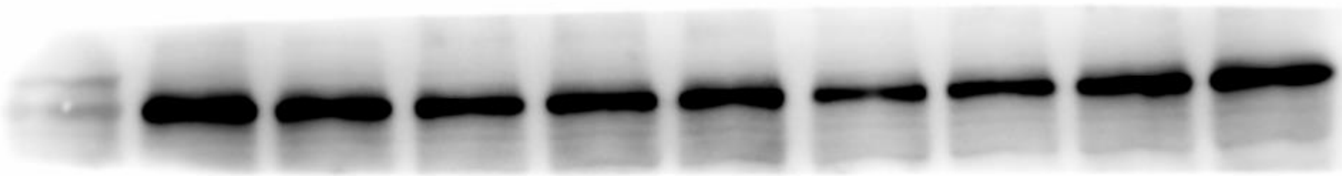

Fig.R1C

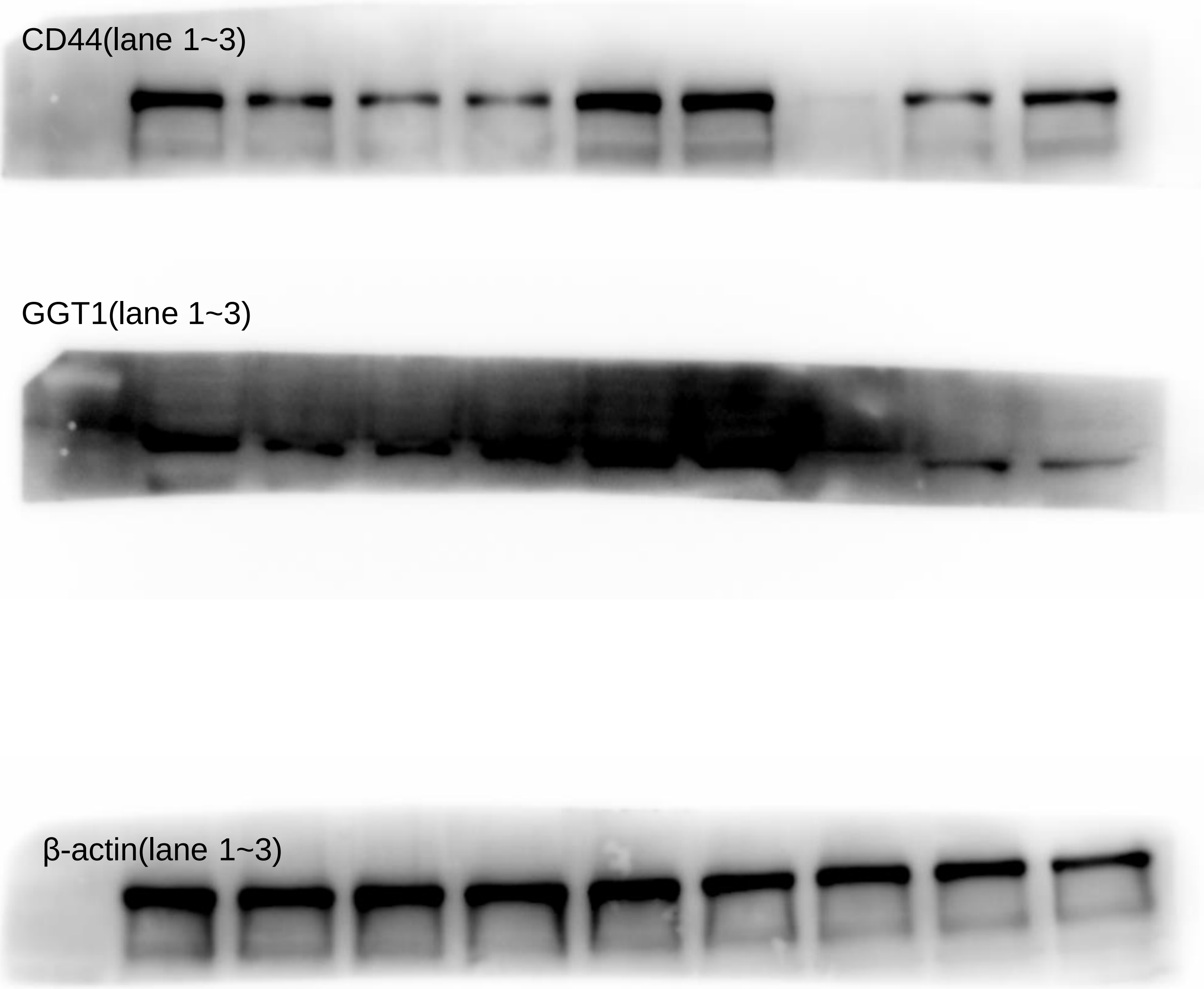

Supplement: Supplementary file 2 — Supplementary Material 2. [file 13046_2024_3016_MOESM2_ESM.zip › Supplementary Material 2.pdf]
