# Supplementary material for: Ropivacaine as a novel AKT1 specific inhibitor regulates the stemness of breast cancer
Source: J Exp Clin Cancer Res. 2024 Mar 25;43:90. doi: 10.1186/s13046-024-03016-9 (PMC10962119; doi:10.1186/s13046-024-03016-9)

Fig.1A

MCF-7-BLANK

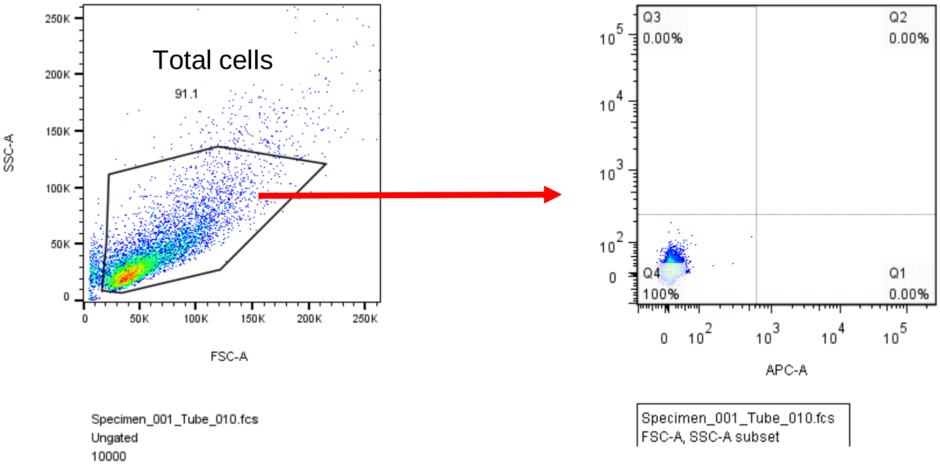

MCF-7-CD24

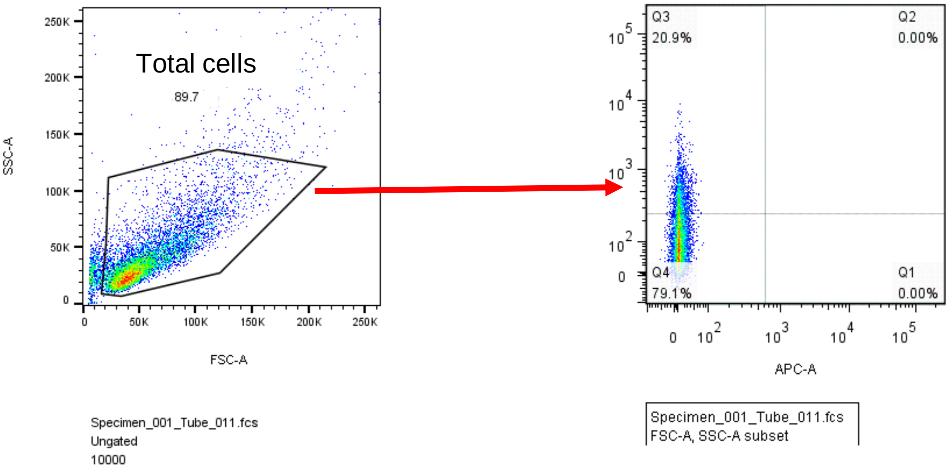

MCF-7-CD44

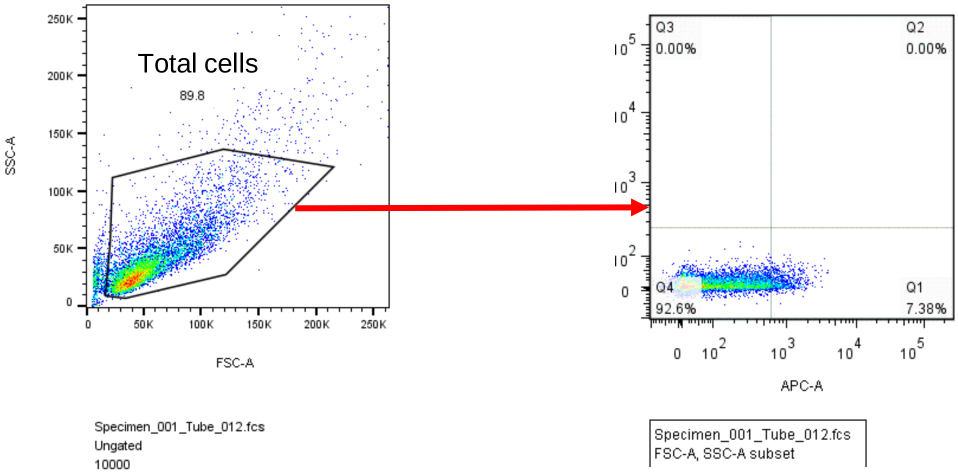

Fig.1A

MDA-MB-231-BLANK

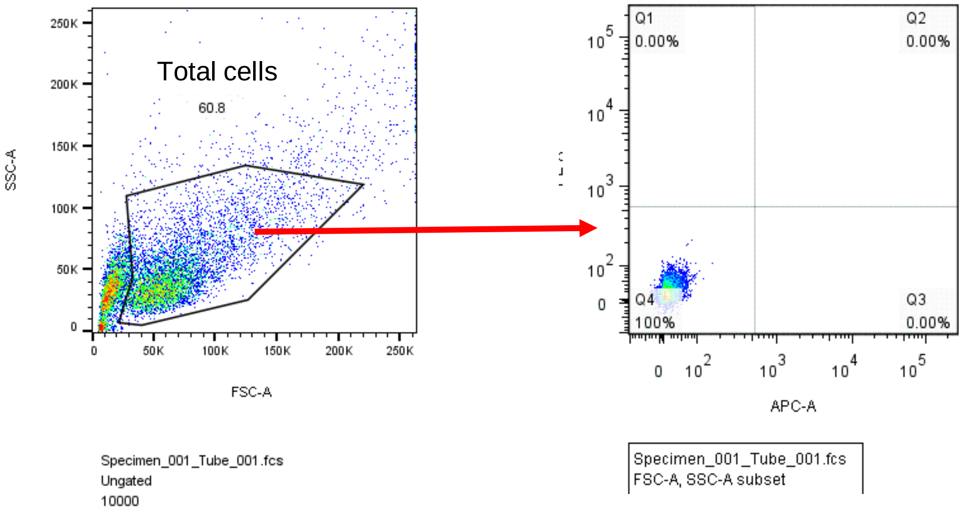

MDA-MB-231-CD24

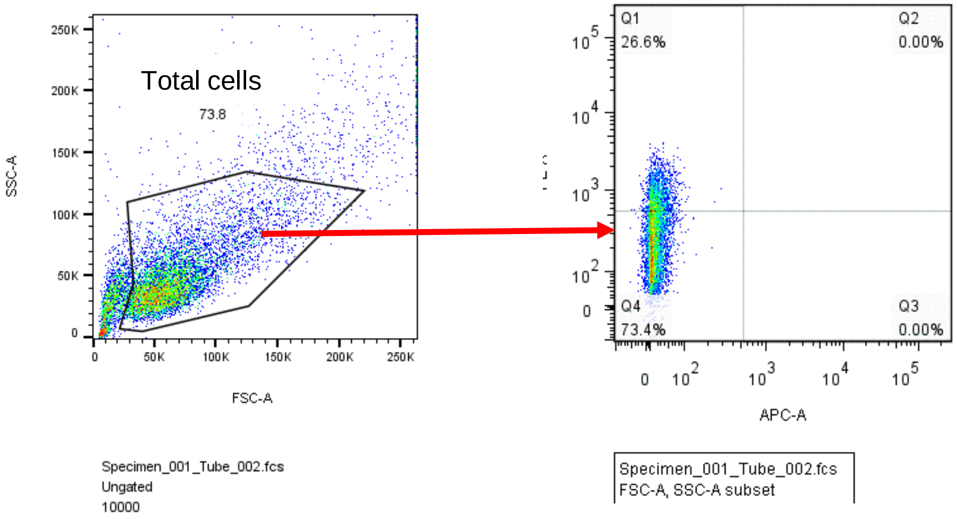

MDA-MB-231-CD44

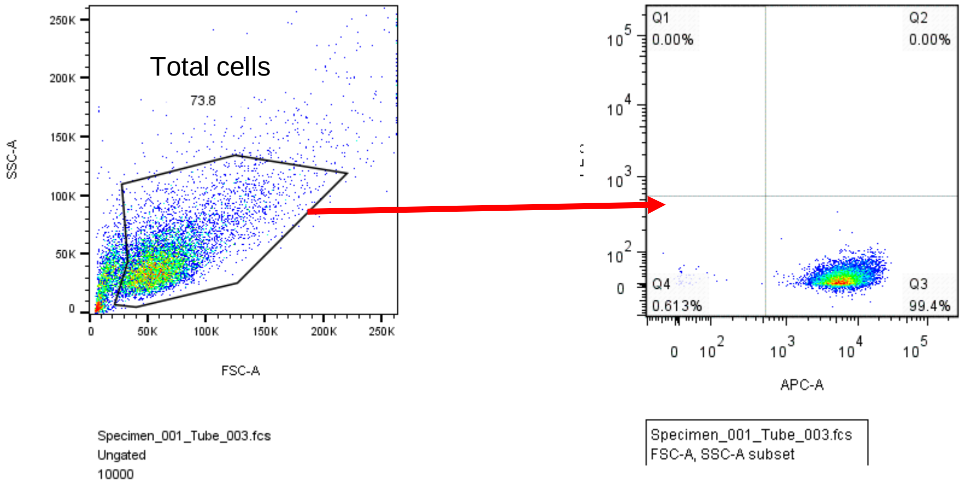

Fig.1G

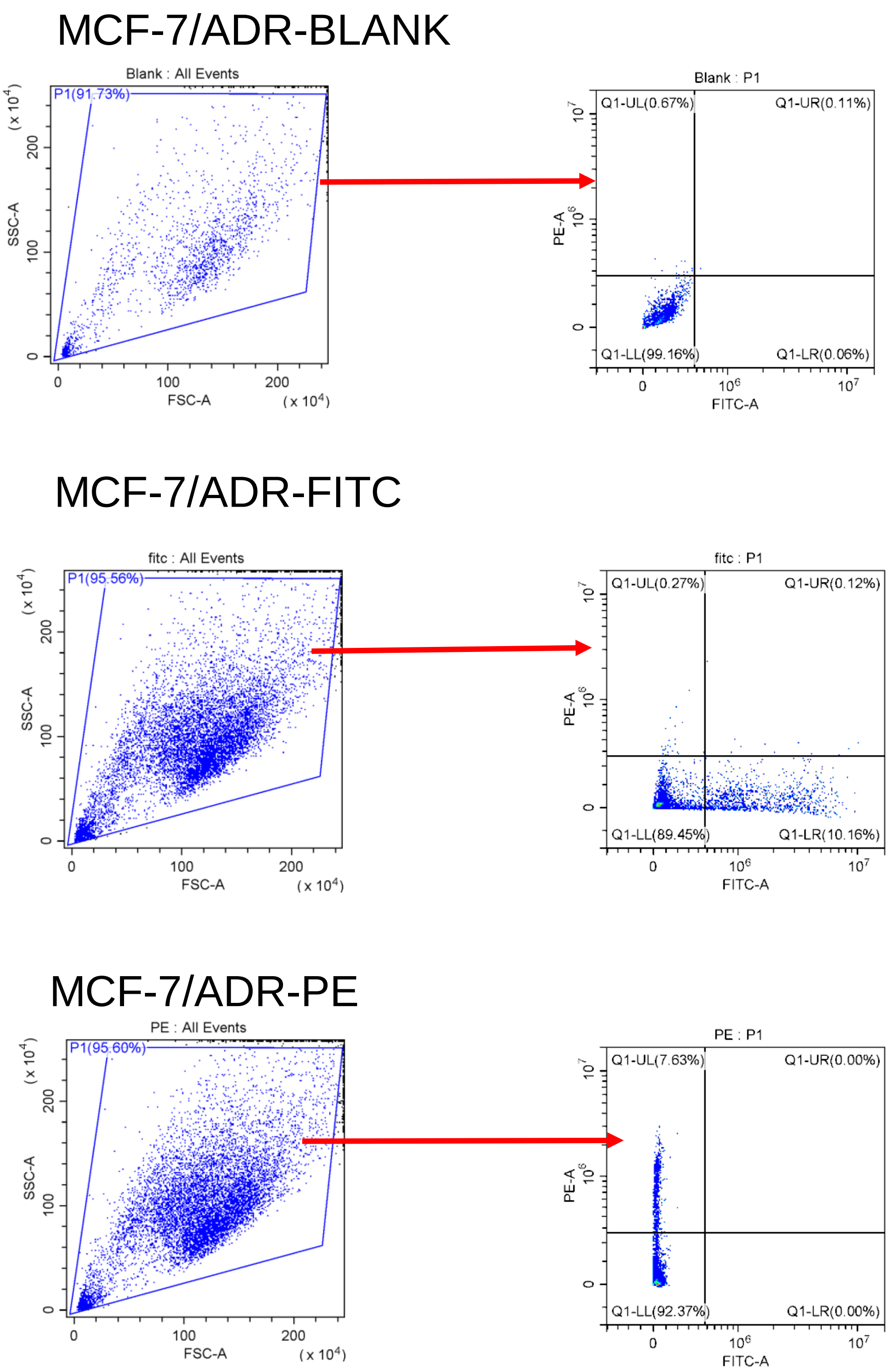

MCF-7/ADR-BLANK
Blank : All Events
P1(91.73%)
SSC-A
FSC-A
Blank : P1
Q1-UL(0.67%)
Q1-UR(0.11%)
Q1-LL(99.16%)
Q1-LR(0.06%)
PE-A
FITC-A
MCF-7/ADR-FITC
fitc : All Events
P1(95.56%)
fitc : P1
Q1-UL(0.27%)
Q1-UR(0.12%)
Q1-LL(89.45%)
Q1-LR(10.16%)
MCF-7/ADR-PE
PE : All Events
P1(95.60%)
PE : P1
Q1-UL(7.63%)
Q1-UR(0.00%)
Q1-LL(92.37%)
Q1-LR(0.00%)

Fig.7D

MCF-7-BLANK

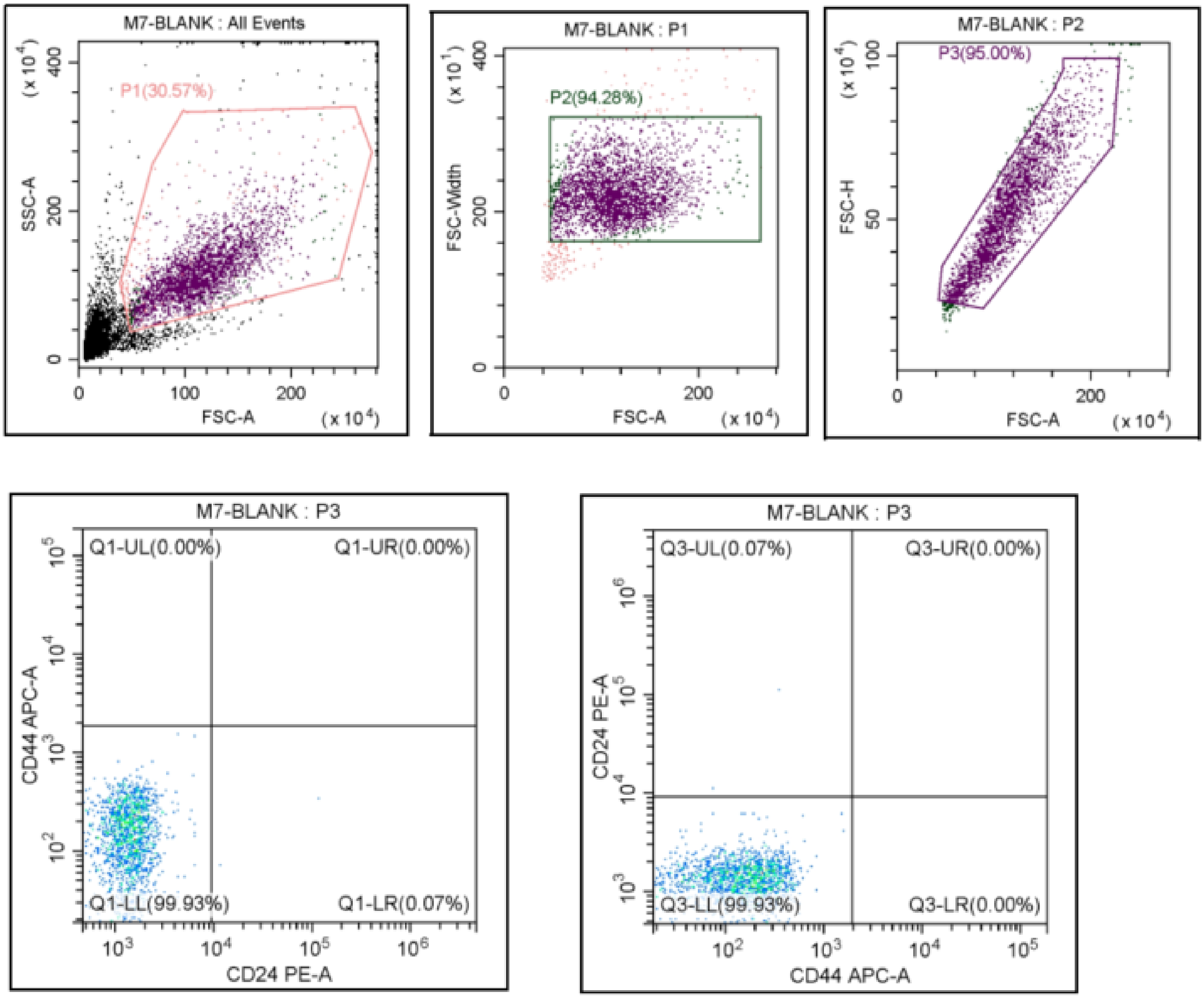

Fig.7D

MCF-7-CD24

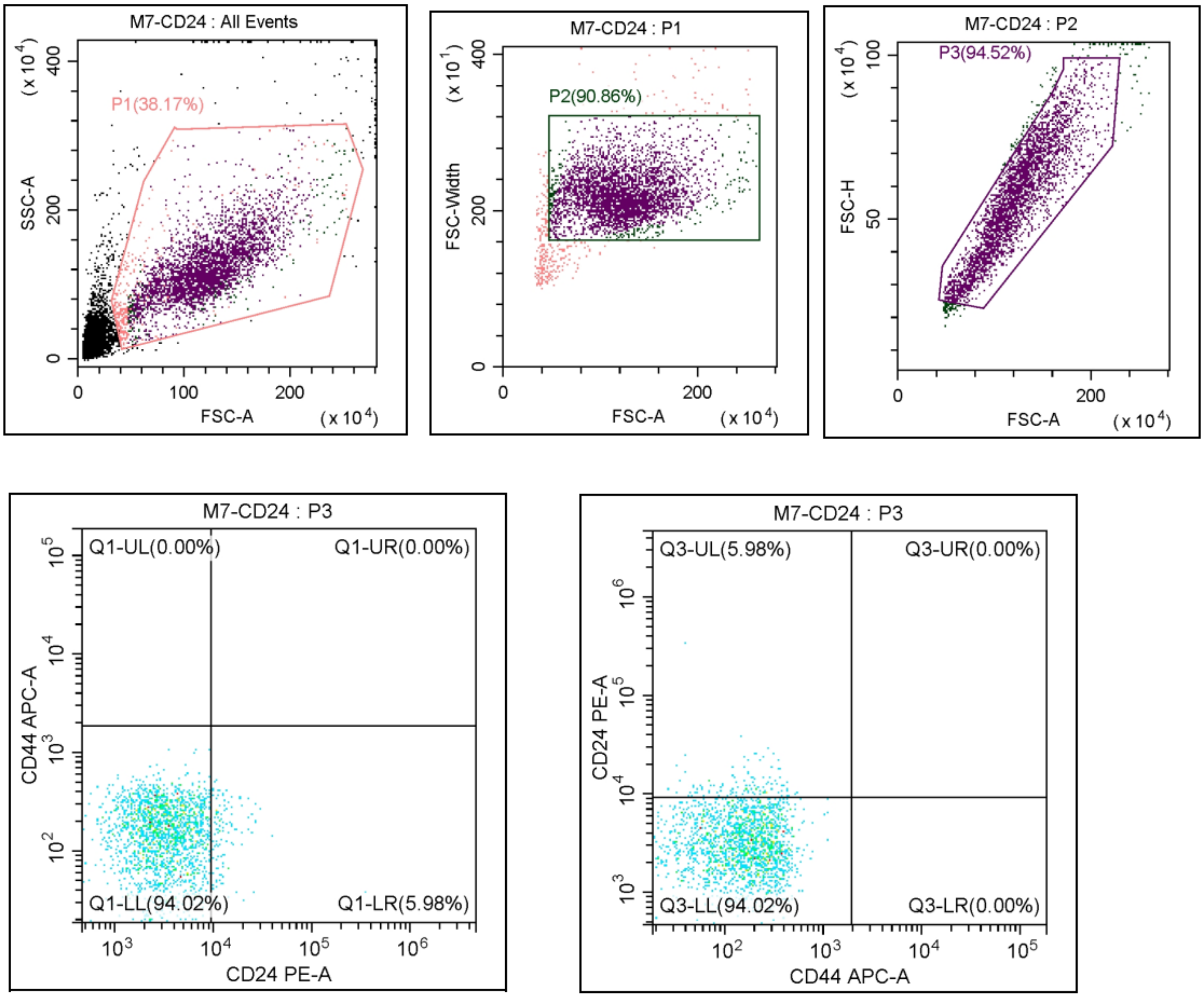

Fig.7D

MCF-7-CD44

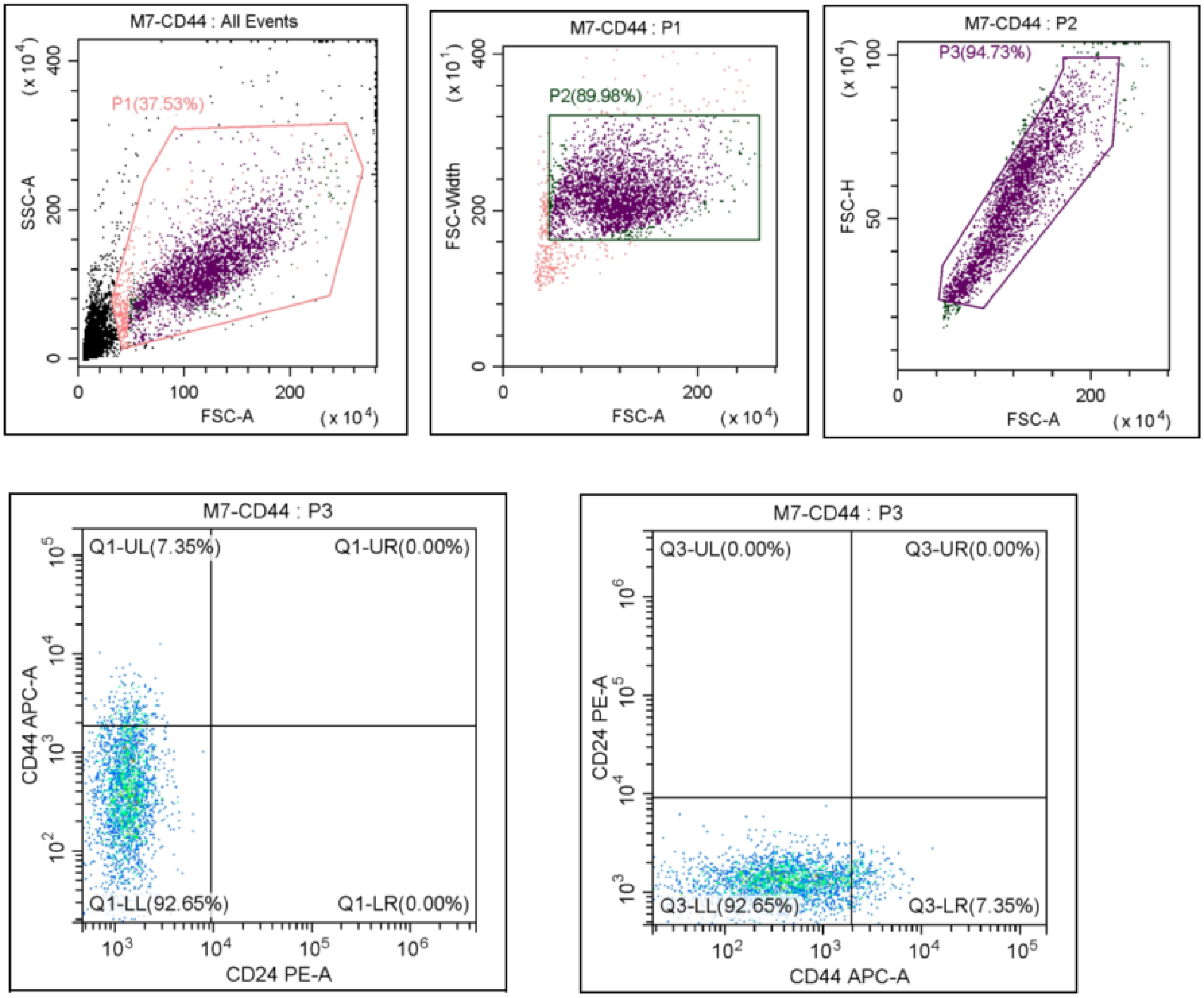

Fig.7H

MCF-7/ADR-BLANK

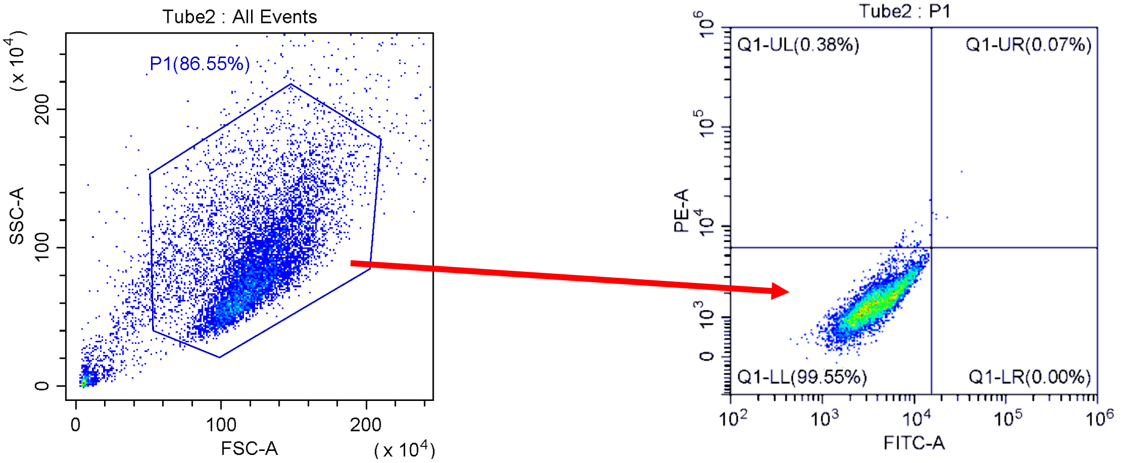

MCF-7/ADR-PE

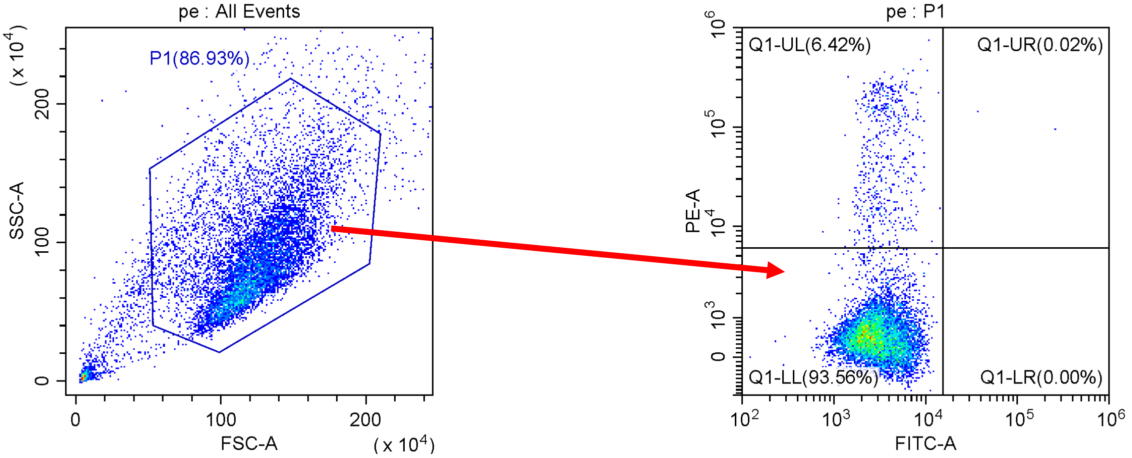

MCF-7/ADR-FITC

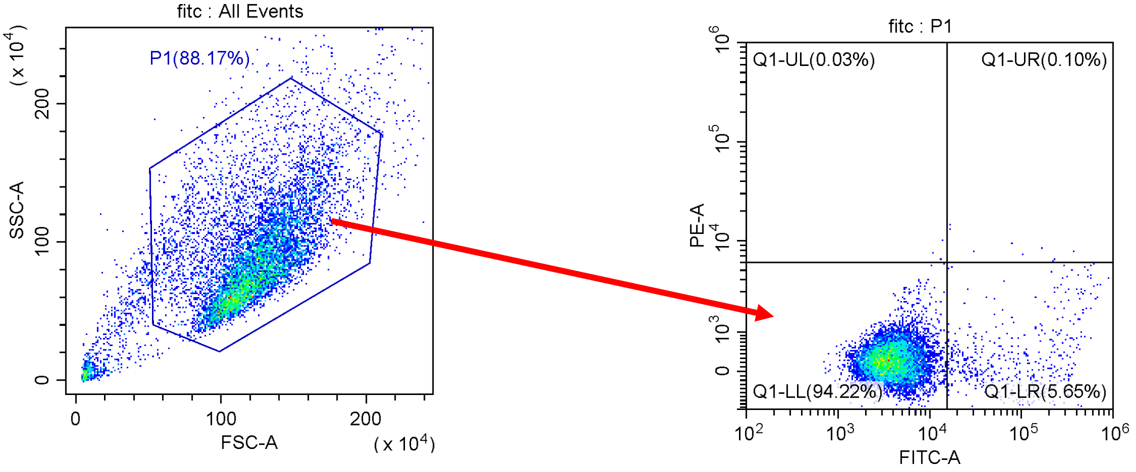

Fig.S2D

MDA-MB-231-BLANK

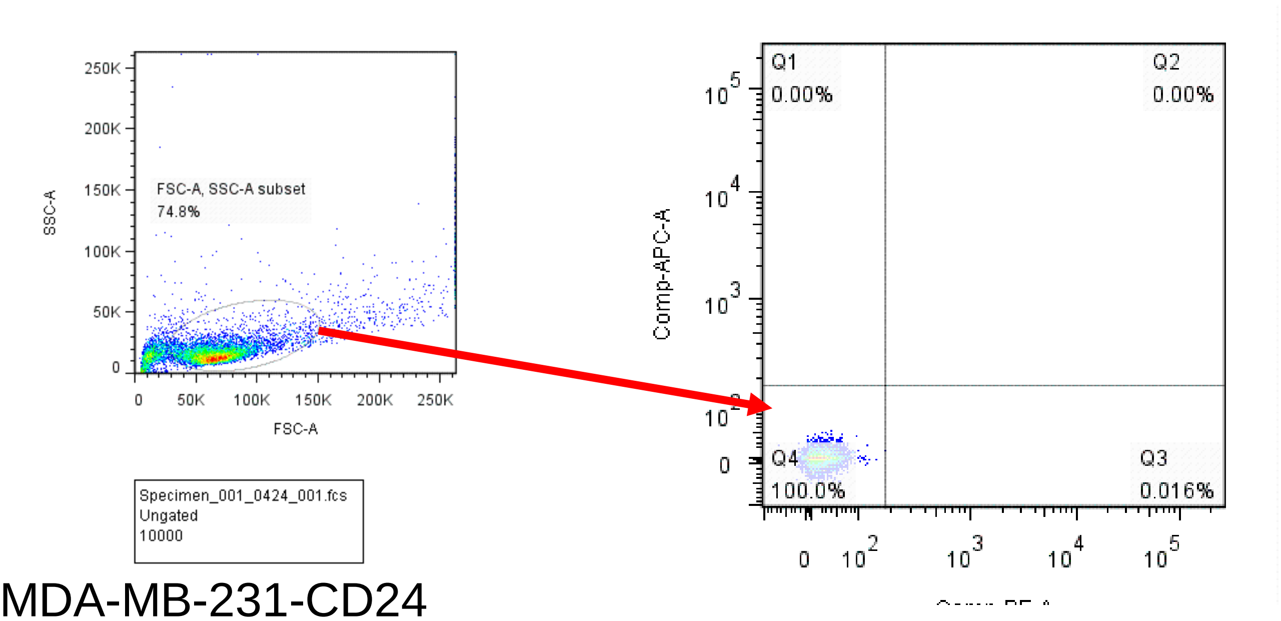

MDA-MB-231-CD24

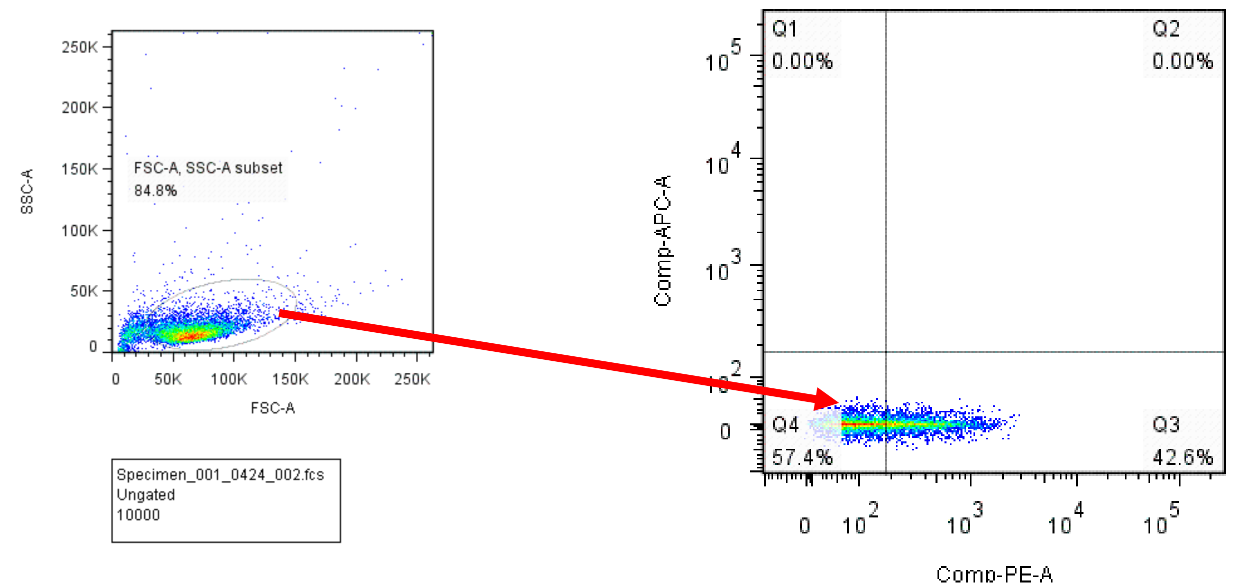

MDA-MB-231-CD44

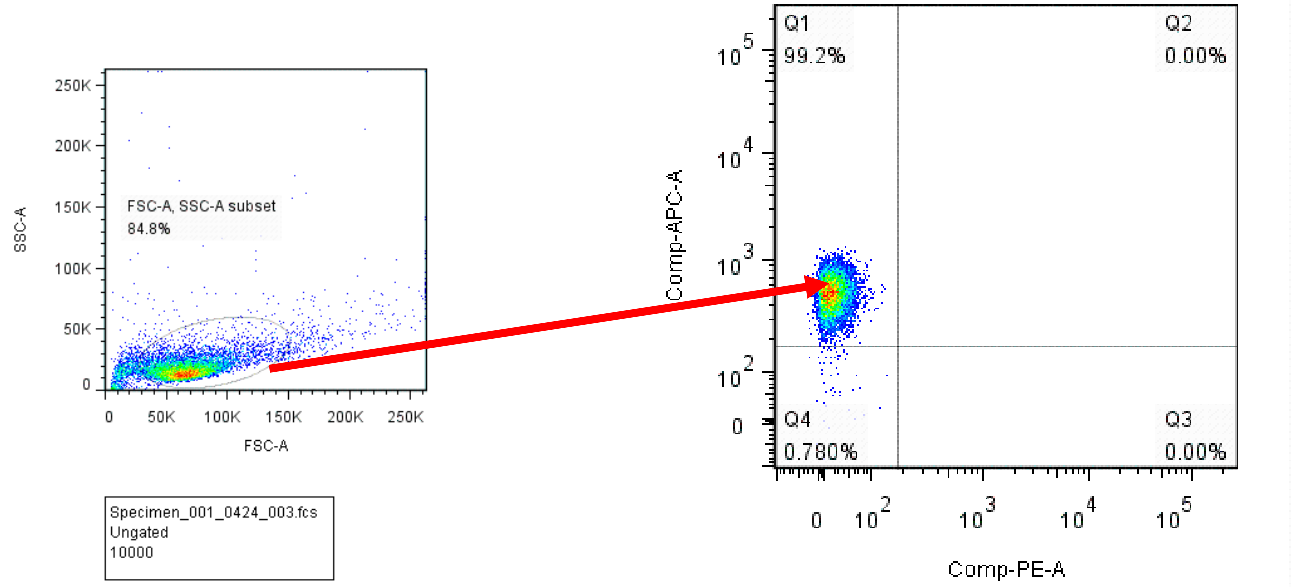

Fig.10D

MCF-7-BLANK

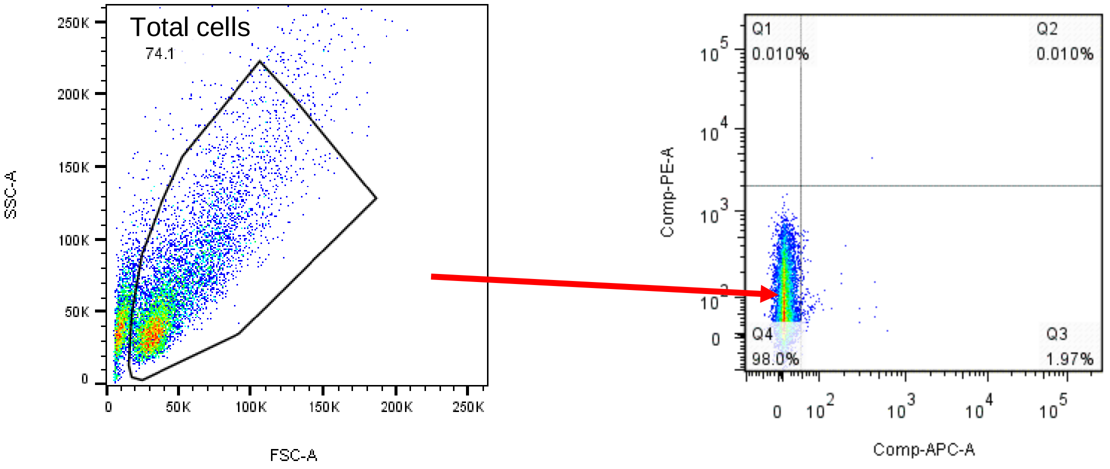

MCF-7-CD44

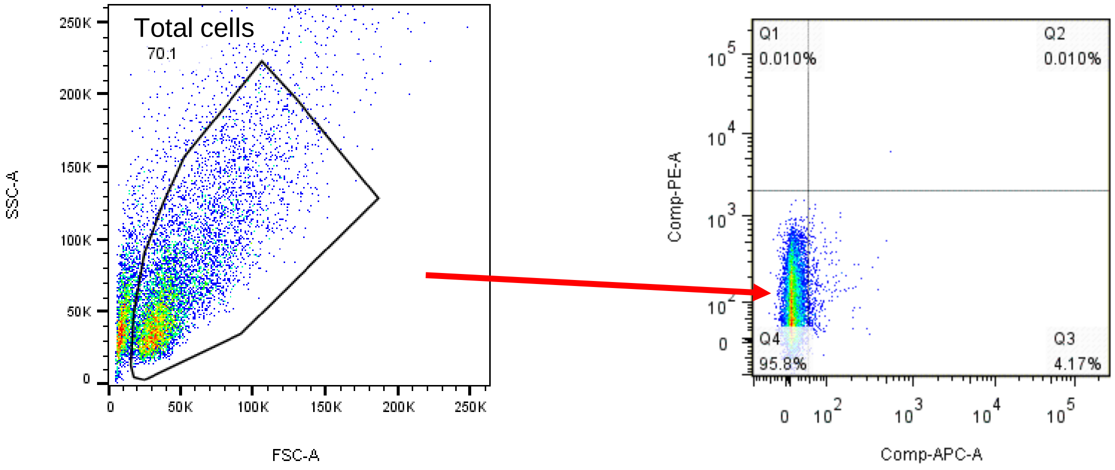

MCF-7-CD24

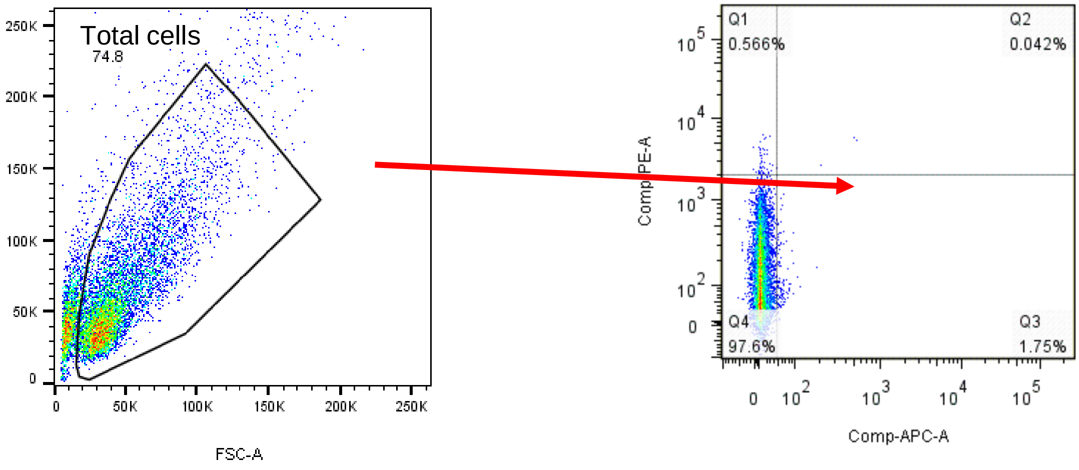

Fig.10H

MCF-7/ADR-BLANK

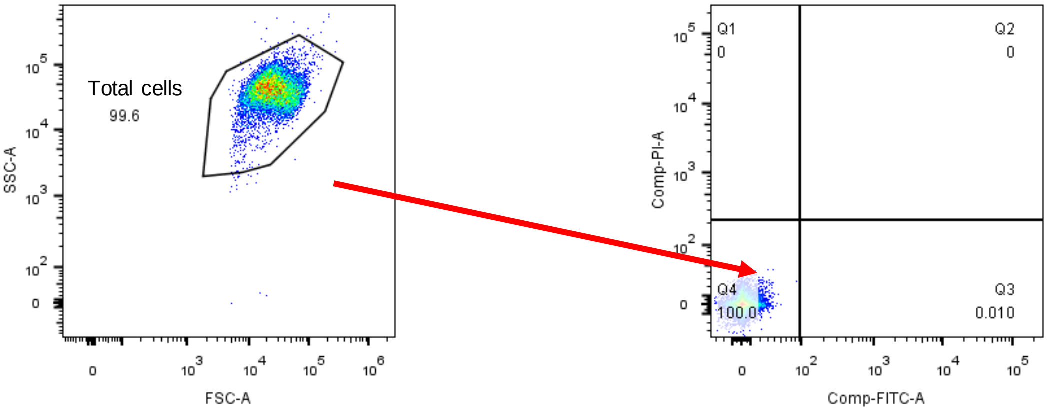

MCF-7/ADR-PE

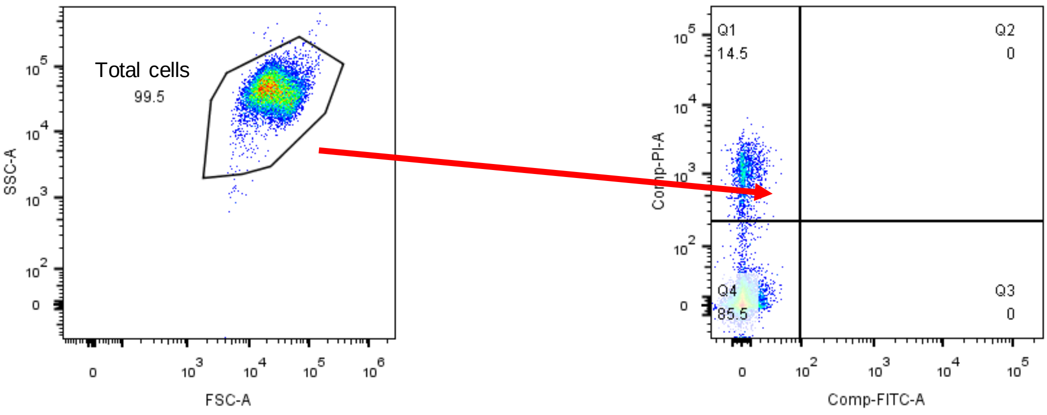

MCF-7/ADR-FITC

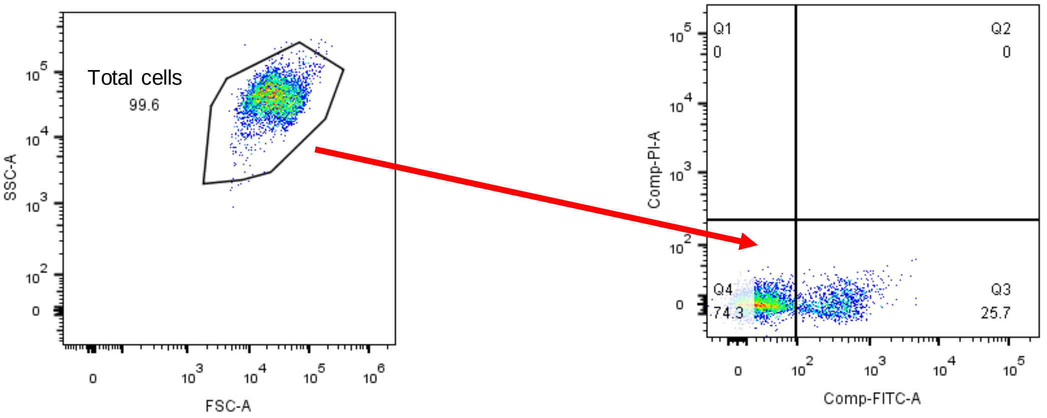

Fig.S4D

MDA-MB-231-BLANK

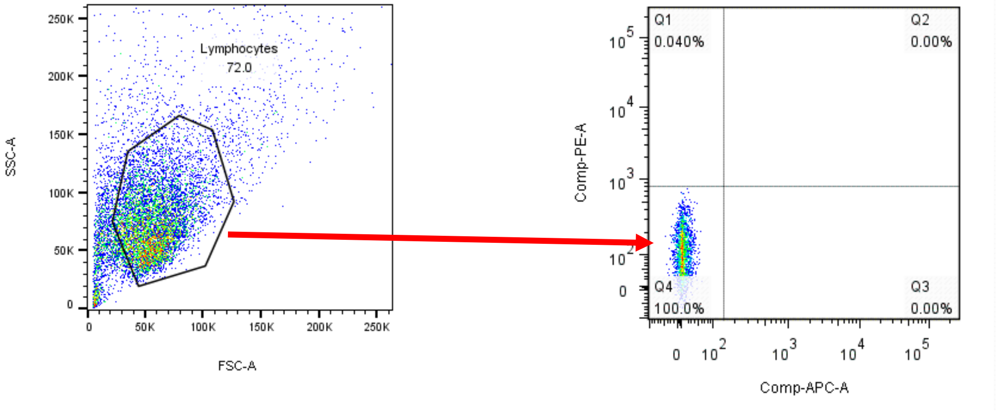

MDA-MB-231-CD24

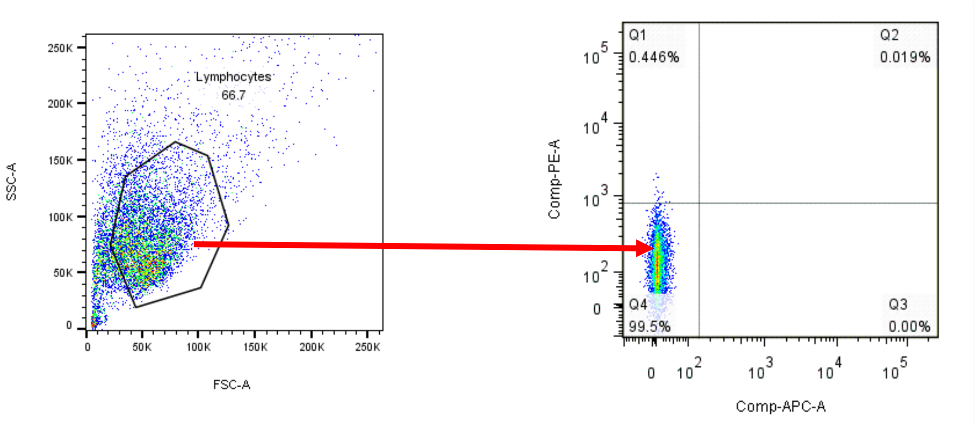

MDA-MB-231-CD44

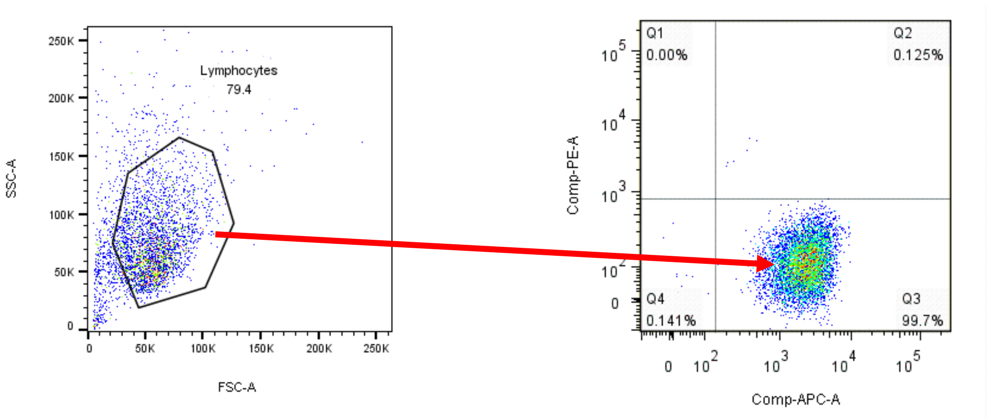

Supplement: Supplementary file 2 — Supplementary Material 2. [file 13046_2024_3016_MOESM2_ESM.zip › Supplementary Material 3.pdf]
